# Molecular and Immunomodulatory Mechanisms of Statins in Inflammation and Cancer Therapeutics with Emphasis on the NF-κB, NLRP3 Inflammasome, and Cytokine Regulatory Axes

**DOI:** 10.3390/ijms26178429

**Published:** 2025-08-29

**Authors:** Sara Khan, Bintul Huda, Farida Bhurka, Rajashree Patnaik, Yajnavalka Banerjee

**Affiliations:** Department of Basic Medical Sciences, College of Medicine, Mohammed Bin Rashid University of Medicine and Health Sciences, Dubai Health, Al Razi St, Umm Hurair 2, Dubai Healthcare City, Dubai 505055, United Arab Emiratesbintul.huda@students.mbru.ac.ae (B.H.);

**Keywords:** statins, chronic inflammation, diabetes mellitus, cancer, osteoarthritis (OA), rheumatoid arthritis (RA), chronic obstructive pulmonary disease (COPD)

## Abstract

Statins, primarily prescribed for their lipid-lowering effects, have garnered significant attention for their potent anti-inflammatory effects. This review explores the underlying molecular pathways and clinical relevance of statins’ anti-inflammatory actions, extending beyond cardiovascular disease management to chronic inflammatory conditions and oncological applications. The lipid-lowering effect of statins stems from their ability to suppress HMG-CoA reductase, a crucial enzyme in cholesterol synthesis; however, their pleiotropic effects include modulation of critical inflammatory pathways such as the inhibition of NF-κB signalling, a reduction in pro-inflammatory cytokine production, and enhancement of endothelial function. We delve into the molecular pathways influenced by statins, including their effects on inflammatory mediators like C-reactive protein (CRP), interleukins (IL-6, IL-1β), and tumour necrosis factor-alpha (TNF-α). Clinical evidence supporting the efficacy of statins in managing chronic inflammatory diseases, such as rheumatoid arthritis, chronic obstructive pulmonary disease, diabetes, and osteoarthritis, is critically reviewed. Additionally, we investigate the emerging role of statins in oncology, examining their impact on inflammation-driven carcinogenesis, tumour microenvironment modulation, and cancer progression. Despite their broad therapeutic potential, the safety profile of statins, particularly concerning adverse effects such as myopathy, hepatotoxicity, and potential diabetes risk, is discussed. Controversies surrounding the extent of their anti-inflammatory benefits and the variability in patient responses are also addressed. This review consolidates the current literature, elucidating the biochemical mechanisms underlying the anti-inflammatory properties of statins and evaluating their clinical applications and associated controversies. Future research directions are identified, including the development of novel statin analogues with enhanced anti-inflammatory effects and the investigation of new therapeutic indications in inflammatory diseases and cancer. By providing an in-depth analysis, this review underscores the expanding therapeutic scope of statins and advocates for their integration into broader clinical strategies for the management of inflammation and cancer.

## 1. Introduction

Statins, constituting a pharmacological class of competitive inhibitors targeting 3-hydroxy-3-methylglutaryl–coenzyme A (HMG-CoA) reductase—the catalytic arbiter of the rate-limiting step in the mevalonate pathway of endogenous cholesterol biosynthesis—have emerged as a principal therapeutic axis in the prophylaxis and longitudinal management of atherosclerotic cardiovascular disease. By orchestrating a sustained decrement in circulating low-density lipoprotein cholesterol (LDL-C) concentrations, these agents attenuate lipid-driven endothelial injury, modulate plaque biology, and substantially diminish the incidence of major adverse cardiovascular events across both primary- and secondary-prevention cohorts. Key clinical trials, including the Scandinavian Simvastatin Survival Study (4S) [1], the Heart Protection Study (HPS) [2], the Cholesterol and Recurrent Events (CARE) trial [3], the Long-Term Intervention with Pravastatin in Ischemic Disease (LIPID) trial [4], the Pravastatin or Atorvastatin Evaluation and Infection Therapy (PROVE-IT TIMI 22) trial [5], the Justification for the Use of Statins in Prevention: an Intervention Trial Evaluating Rosuvastatin (JUPITER) [6], and the West of Scotland Coronary Prevention Study (WOSCOPS) [7] have demonstrated these effects, establishing statins as a foundational therapy in cardiovascular disease management. However, beyond their well-established lipid-lowering properties, statins have garnered significant attention for their potent anti-inflammatory effects, which extend their therapeutic potential far beyond the realm of cardiovascular disease. For example, the JUPITER trial demonstrated that rosuvastatin significantly reduced high-sensitivity C-reactive protein (hs-CRP) levels [6], a marker of inflammation, while the Controlled Rosuvastatin Multinational Trial in Heart Failure (CORONA) trial observed a reduction in inflammatory biomarkers in heart failure patients treated with rosuvastatin [8]. Similary in the Anglo-Scandinavian Cardiac Outcomes Trial–Lipid Lowering Arm (ASCOT-LLA) trial, a reduction in inflammatory biomarkers such as IL-6 and TNF-α in patients treated with atorvastatin was observed, highlighting its anti-inflammatory benefits alongside its lipid-lowering effects [9].

**Figure 1:**
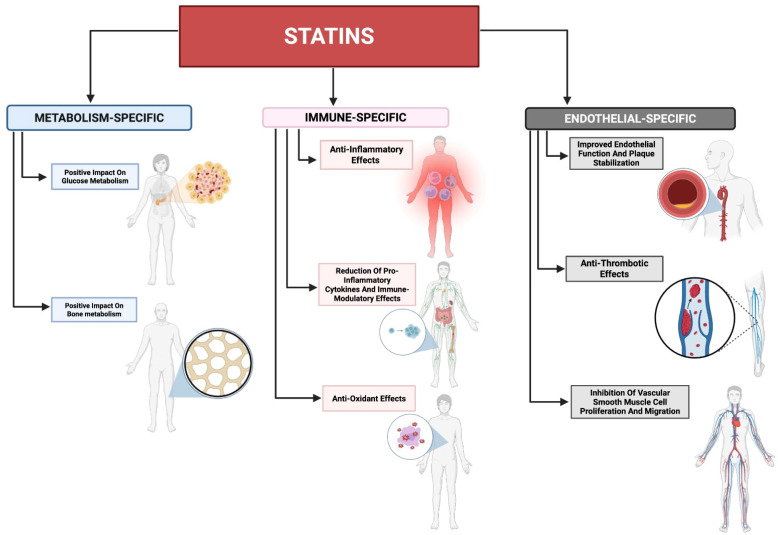
Pleiotropic effects of statins. A summary of statins’ pleiotropic effects beyond their primary lipid-lowering properties that extend into metabolic processes (metabolism-specific), the immune system (immune-specific), and the lymphovascular system (endothelial-specific). These pleiotropic effects of statins can allow statins to be utilized as a therapeutic regimen in multiple conditions.

The pleiotropic effects of statins (Figure 1)—those unrelated to their capacity to induce sustained reductions in atherogenic lipoprotein concentrations—have been the subject of extensive research. These pharmacodynamic effects encompass the perturbation of pivotal pro-inflammatory signalling cascades, most notably the suppression of nuclear factor κ-light-chain-enhancer of activated B cells (NF-κB)-dependent transcriptional activity [10], concomitant with an attenuation of downstream pro-inflammatory cytokine biosynthesis [11], along with improved endothelial function [12]. These effects are of particular interest given the central role of inflammation in the pathogenesis of numerous chronic diseases and malignancies [13].

Inflammation serves as a crucial biological response to harmful stimuli such as pathogens, cellular damage, and irritants [14,15,16]. This process involves the release of numerous inflammatory mediators, including cytokines and chemokines, which coordinate the activation and migration of immune cells to the affected site. Whereas acute inflammation constitutes a temporally restricted, evolutionarily conserved reparative and immunoprotective response, its chronic persistence engenders a maladaptive immunopathological milieu that underpins the etiopathogenesis of a broad spectrum of morbidities, including—but not limited to—rheumatoid arthritis (RA), osteoarthritis (OA), chronic obstructive pulmonary disease (COPD), and diverse malignancies. The latter being a fascinating niche to investigate as chronic inflammation is a well-recognized driver of carcinogenesis, promoting tumour initiation, progression, and metastasis [17,18,19]. Statins, by modulating inflammatory pathways, may advantageously affect the tumour microenvironment (TME) and inhibit cancer progression.

Epidemiological evidence derived from large-scale, population-based cohorts has suggested an inverse association between statin exposure and the incidence of select malignancies, notably breast, prostate, and colorectal carcinomas [20]. Complementary preclinical investigations have elucidated pleiotropic oncostatic mechanisms attributable to statins, encompassing the attenuation of neoplastic cell proliferation, the induction of programmed cell death, and the suppression of tumour progression across diverse experimental models [21]. Furthermore, clinical trials are currently in progress to assess the effectiveness of statins as adjuvant therapy in cancer treatment (refer below for details), exploring their potential to enhance the effects of conventional chemotherapy and radiotherapy. Statins are also being explored for their potential benefits in specific diseases where inflammation plays a pivotal role, such as psoriasis, multiple sclerosis, and inflammatory bowel disease (IBD). Therefore, the anti-inflammatory effects of statins offer a promising potential for therapeutic intervention in these conditions.

Despite the broad therapeutic potential of statins, their safety profile warrants careful consideration. Common adverse effects of statin therapy include myopathy, hepatotoxicity, and an increased risk of diabetes [22,23]. These side effects, although generally rare, necessitate a thorough risk-benefit analysis when prescribing statins, particularly for long-term use in chronic inflammatory conditions or cancer. Controversies also exist regarding the extent of the anti-inflammatory benefits of statins and the variability in patient responses. Genetic factors, comorbidities, and concurrent medications can influence the efficacy and safety of statin therapy [24], underscoring the need for personalized treatment approaches.

This paper reviews the body of evidence surrounding statins’ anti-inflammatory effects, focusing on the biochemical mechanisms at play as well as their practical clinical implications and ongoing controversies. It highlights important avenues for future research, including the investigation of novel therapeutic applications of statins in inflammatory diseases and cancer. By thoroughly analyzing current data, the paper highlights the widening role of statins in medicine and calls for their incorporation into treatment strategies targeting inflammation, especially within oncology.

## 2. Statins at a Glance

**Figure 2:**
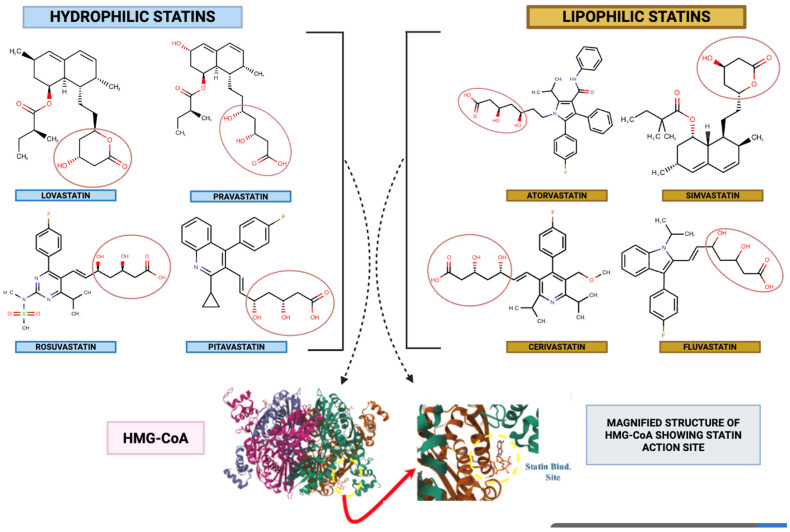
Classification of statins. An illustration of the commonly utilized statins classified based on hydrophilicity and lipophilicity, along with their molecular structures. The lipophilic nature of statins allows them to easily penetrate various membranes. On the other hand, hydrophilicity allows them ease of transport in the bloodstream, thus affecting not only their therapeutic potential but also contributing to their possible side effect potential. Furthermore, the figure details the functional group of statins (3,5-dihydroxyheptanoic acid) that is structurally and functionally similar to the active site of HMG-CoA (PDB code 1HW8), allowing for competitive inhibition.

Statins may be dichotomised into hydrophilic and lipophilic subclasses (vide Figure 2), a physicochemical stratification that exerts profound influence upon their pharmacokinetic disposition, tissue penetrance, and attendant adverse-event spectra. The salient agents within each subclass are enumerated in Table 1, which concomitantly catalogues pivotal, large-scale, randomized controlled trials that have served as the evidentiary fulcrum for contemporary lipid-lowering paradigms. These landmark investigations have yielded methodologically rigorous and statistically robust proof-of-concept for the salutary impact of statin therapy upon both primary and secondary prophylaxis of atherosclerotic cardiovascular disease (ASCVD). The aggregate corpus of such trial-derived data constitutes the foundational substrate upon which current American College of Cardiology/American Heart Association (ACC/AHA) clinical practice guidelines are predicated—guidelines that promulgate the initiation of statin pharmacotherapy in individuals manifesting elevated ASCVD risk indices, irrespective of antecedent baseline low-density lipoprotein cholesterol (LDL-C) concentrations, with therapeutic intensity being meticulously titrated in proportion to the calculated global cardiovascular risk burden [25,26].

Hydrophilic statins, exemplified by pravastatin and rosuvastatin, possess a high aqueous solubility profile, resulting in preferential hepatocellular uptake via active, carrier-mediated transport mechanisms, particularly organic anion transporting polypeptides (OATPs). In contrast, lipophilic congeners—such as atorvastatin, simvastatin, and lovastatin—exhibit pronounced lipid solubility, thereby facilitating passive transmembrane diffusion and enabling a broader intracellular distribution across extrahepatic tissues. The biotransformation of statins is predominantly hepatic, engaging a constellation of cytochrome P450 isoenzymes. Among these, CYP3A4 constitutes the principal metabolic axis for atorvastatin, simvastatin, and lovastatin, whereas CYP2C9 predominates in the oxidative metabolism of agents such as fluvastatin and rosuvastatin. This isoform-specific metabolic routing exerts profound influence over their pharmacokinetic disposition, susceptibility to pharmacokinetic–pharmacodynamic interactions, and the qualitative spectrum of adverse event profiles [27,28,29].

Therapeutically, statins are ubiquitously deployed in the pharmacological management of hyperlipidemia, operating through competitive antagonism at the catalytic domain of HMG-CoA reductase, wherein they displace the natural substrate, 3-hydroxy-3-methylglutaryl–coenzyme A. This blockade disrupts the mevalonate biosynthetic cascade (Figure 2), leading to a reduction in endogenous cholesterol synthesis and subsequent upregulation of hepatic LDL receptors, thereby enhancing plasma LDL clearance. The clinical utility of this pharmacodynamic mechanism is underscored by pivotal outcome trials—ASCOT-LLA for atorvastatin [9], the Scandinavian Simvastatin Survival Study (4S) for simvastatin [1], and JUPITER for rosuvastatin [6]—each demonstrating substantial reductions in cardiovascular morbidity and mortality. Cumulatively, these data affirm the dual therapeutic paradigm of statins—potent lipid-lowering efficacy coupled with pleiotropic anti-inflammatory actions—positioning them as indispensable agents within contemporary cardiovascular risk reduction strategies and potentially as modulators of disease processes extending beyond atherosclerosis.

### 2.1. Cholesterol-Lowering Mechanism of Statins

**Figure 3:**
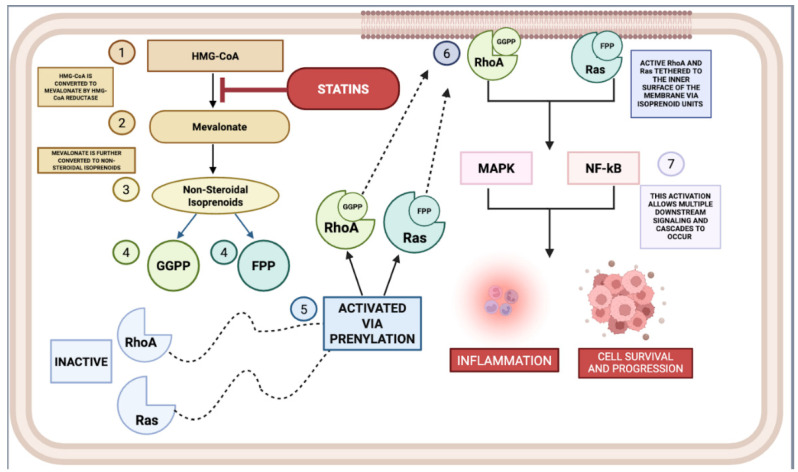
Statins inhibit mevalonate pathway and RhoA and Ras activation. This diagram depicts the mevalonate pathway, detailing how the intermediates, GGPP and FPP, are utilized for activation of small G-proteins RhoA and Ras. HMG-CoA (1) is reduced to mevalonate (2), which leads to downstream conversion into non-steroidal isoprenoids (3), GGPP and FPP (4). RhoA and Ras become activated by these small G-proteins (4) via prenylation, specifically geranylgeranylation via GGPP and farnesylation via FPP. (5) These allow these molecules to be anchored to the cell’s inner membrane (6). The subsequent activation of several downstream signalling and outcomes, such as inflammation and cell progression (7), have been implicated in diseases such as diabetes, cancer, and more. Thus, by inhibiting the enzyme HMG-CoA, statins can inhibit the downstream cascade, allowing the attenuation of further signalling.

Statins exert their hypocholesterolemic activity via a complex, multistage pharmacodynamic cascade, the fulcrum of which is the high-affinity, reversible inhibition of 3-hydroxy-3-methylglutaryl–coenzyme A (HMG-CoA) reductase. This microsomal, NADPH-dependent oxidoreductase represents the committed, rate-limiting enzymatic locus within the mevalonate biosynthetic pathway, catalyzing the stereospecific reduction in HMG-CoA to mevalonate—a pivotal early-stage metabolite that governs the flux and overall throughput of endogenous cholesterol synthesis (Figure 3). By occupying the catalytic site in a manner structurally mimetic of the natural substrate, statins arrest this critical step, thereby attenuating intracellular cholesterol pools, triggering compensatory upregulation of hepatic low-density lipoprotein (LDL) receptor expression, and enhancing receptor-mediated endocytosis of circulating LDL particles. The cholesterol-lowering efficacy of statins thus emerges from a synergistic interplay of primary enzymatic blockade with downstream regulatory adaptations, culminating in a pronounced reduction in plasma LDL concentrations and a concomitant improvement in overall lipid profile.

#### 2.1.1. Inhibition of HMG-CoA Reductase

Owing to their structural congruence with the endogenous substrate HMG-CoA (Figure 3), statins engage in high-affinity, competitive occupancy of the catalytic domain of HMG-CoA reductase. This substrate mimicry sterically hinders enzymatic turnover, thereby abrogating the reductive conversion of HMG-CoA to mevalonate [30]. The pharmacological significance of this blockade lies in the fact that mevalonate constitutes an obligatory biosynthetic precursor not only for cholesterol but also for an array of isoprenoid derivatives that are essential to diverse cellular functions [31]. Suppression of mevalonate biosynthesis thus precipitates a coordinated series of downstream metabolic perturbations, culminating in a sustained diminution of systemic cholesterol levels.

##### Decreased Cholesterol Synthesis in Hepatocytes

Through the targeted inhibition of HMG-CoA reductase, the de novo biosynthetic output of cholesterol within hepatocytes is markedly attenuated. Cholesterol, a pivotal structural constituent of cellular bilayers and an indispensable substrate for the biosynthesis of bile acids, steroid hormones, and vitamin D, thus becomes limited at its primary site of production. The hepatocentric suppression of cholesterol synthesis induced by statins precipitates a pronounced intracellular depletion, which in turn elicits a suite of homeostatic counter-regulatory responses, most notably the transcriptional upregulation of LDL receptors and the concomitant diminution of isoprenoid intermediates.

#### 2.1.2. Upregulation of LDL Receptors

Following the suppression of endogenous cholesterol biosynthesis, hepatocytes initiate a compensatory transcriptional programme characterized by marked upregulation of LDL receptor expression at the plasma membrane. This adaptive shift augments the capacity for receptor-mediated sequestration of circulating LDL particles, the predominant cholesterol transport vehicles within the systemic circulation. Ligand–receptor engagement facilitates clathrin-dependent endocytosis, delivering LDL cargo into endosomal–lysosomal compartments, where hydrolytic degradation liberates free cholesterol for intracellular utilization. This process simultaneously attenuates the extracellular LDL-C burden, and the receptor upregulation-driven acceleration of LDL-C clearance constitutes a pivotal mechanistic axis underpinning the lipid-lowering efficacy of statins.

#### 2.1.3. Reduction in VLDL Secretion

Very-low-density lipoprotein (VLDL), a triglyceride- and cholesterol-enriched lipoprotein fraction synthesized within the hepatic parenchyma, undergoes progressive intravascular remodelling to yield LDL as its metabolic endpoint. Statin-mediated inhibition of cholesterol biosynthesis, coupled with attenuation of triglyceride availability, constrains the substrate pool required for VLDL assembly within hepatocytes. This restriction in precursor lipid supply translates into a diminished rate of VLDL secretion into the circulation, thereby limiting the downstream intravascular generation of LDL particles and contributing to the overall reduction in atherogenic lipoprotein burden.

#### 2.1.4. Inhibition of Isoprenoid Synthesis

Inhibition of HMG-CoA reductase by statins concomitantly suppresses the biosynthetic generation of critical isoprenoid intermediates, notably farnesyl pyrophosphate (FPP) and geranylgeranyl pyrophosphate (GGPP) (Figure 3). These polyisoprenoid derivatives serve as indispensable lipid moieties for the post-translational prenylation of a diverse repertoire of proteins integral to intracellular cholesterol trafficking and signal transduction. Among these, the covalent attachment of isoprenoid chains to small GTP-binding proteins such as Ras and Rho is essential for their membrane localisation and functional activation, thereby governing key regulatory axes in cellular proliferation, differentiation, and survival. Through attenuation of isoprenoid synthesis, statins exert secondary modulatory effects on these signalling cascades, with consequent ramifications for lipid homeostasis and broader aspects of cellular physiology.

#### 2.1.5. Impact on Sterol Regulatory Element-Binding Proteins (SREBPs)

The diminution of intracellular cholesterol concentration exerts a regulatory influence on the activity of sterol regulatory element-binding proteins (SREBPs), a family of membrane-bound transcription factors orchestrating the expression of genes implicated in cholesterol and fatty acid biosynthetic pathways [32]. In the basal state, SREBPs remain sequestered within the endoplasmic reticulum (ER) membrane as inactive precursors. A decline in cholesterol availability initiates their proteolytic activation, enabling the release and nuclear translocation of the mature transcriptionally competent forms, which subsequently upregulate the expression of LDL receptor genes and enzymatic mediators of cholesterol synthesis [33,34]. Under conditions of HMG-CoA reductase inhibition by statins, however, the SREBP-mediated transcriptional response is functionally biassed towards robust enhancement of LDL receptor expression, thereby potentiating plasma LDL-C clearance rather than restoring endogenous cholesterol synthesis [35,36,37].

#### 2.1.6. Reduction in Circulating LDL-C and Other Lipoproteins

The integrated outcome of attenuated cholesterol biosynthesis, augmented LDL receptor upregulation, diminished hepatic VLDL output, and suppression of isoprenoid generation culminates in a pronounced reduction in circulating LDL-C concentrations. In addition, statin therapy is associated with modest decrements in plasma triglyceride levels and slight elevations in high-density lipoprotein (HDL) cholesterol [38]. Collectively, these shifts engender a favourable remodelling of the lipid profile, thereby mitigating atherogenic burden and conferring a measurable reduction in the incidence of atherosclerotic cardiovascular events.

In conclusion, through the comprehensive inhibition of HMG-CoA reductase and the subsequent cascade of metabolic changes, statins effectively lower plasma cholesterol levels, primarily by reducing LDL-C. This mechanism not only involves direct reduction in cholesterol synthesis but also enhances cholesterol clearance from the bloodstream, decreases VLDL secretion, and influences other lipid-related pathways.

#### 2.1.7. Reflection from Clinical Trials

A comprehensive analysis of clinical trials involving statins provides critical insights into their cholesterol-lowering effects and cardiovascular benefits (Table 1). These trials encompass various statins, each with unique pharmacological properties, but collectively, they underscore the importance of LDL-C reduction in managing cardiovascular risk. Below, we detail the findings from key clinical trials for each statin, including their impact on cholesterol levels and cardiovascular outcomes.

Atorvastatin has undergone extensive evaluation within multiple large-scale, randomized, controlled clinical investigations. The Anglo-Scandinavian Cardiac Outcomes Trial—Lipid-Lowering Arm (ASCOT-LLA), enrolling 10,305 hypertensive subjects with normocholesterolemia or cholesterol concentrations below the population mean [9], demonstrated that atorvastatin administration yielded an approximate 35% decrement in LDL-C concentrations, concomitantly effecting a 36% relative risk reduction in major cardiovascular events. Notably, this therapeutic benefit was consistently observed across diverse stratified subpopulations, including those with comparatively low baseline cholesterol, thereby indicating that the cardioprotective effects of atorvastatin extend beyond its conventional lipid-lowering indications. Complementing these findings, the Collaborative Atorvastatin Diabetes Study (CARDS) specifically investigated individuals with type 2 diabetes mellitus who did not exhibit substantially elevated LDL-C levels [39]. In this cohort, atorvastatin therapy was associated with a 37% diminution in the incidence of major cardiovascular events, reinforcing the paradigm that statin-mediated risk reduction in diabetic populations is operative irrespective of initial LDL-C status.

Simvastatin has been rigorously interrogated in multiple landmark clinical investigations, among which the Scandinavian Simvastatin Survival Study (4S) remains one of the most seminal [1]. This randomized trial enrolled 4444 subjects with established coronary heart disease and hypercholesterolemia, demonstrating that simvastatin therapy elicited a ~35% reduction in LDL-C concentrations and a 30% decrease in all-cause mortality—the latter being principally attributable to a 42% diminution in coronary heart disease-related deaths. These data provided compelling, high-grade evidence substantiating the cholesterol-lowering and survival-prolonging properties of statins within the framework of secondary prevention. Building on these foundational results, the Heart Protection Study (HPS) recruited in excess of 20,000 participants identified as being at elevated risk for cardiovascular events, including individuals with diabetes mellitus, peripheral arterial disease, and prior cerebrovascular pathology [40]. The HPS demonstrated a 24% reduction in major vascular events with simvastatin therapy, irrespective of baseline cholesterol concentrations—a pivotal observation that substantiated the broader clinical utility of statins in high-risk populations, even in the absence of overt LDL-C elevation.

Lovastatin, among the earliest statin agents to be developed and clinically deployed, underwent pivotal evaluation in the Air Force/Texas Coronary Atherosclerosis Prevention Study (AFCAPS/TexCAPS) [41]. This landmark randomized, primary prevention trial enrolled 6605 individuals without prior clinical manifestations of cardiovascular disease and demonstrated that lovastatin therapy conferred a 37% relative risk reduction in the incidence of a first major acute coronary event. This outcome was mechanistically associated with an approximate 25% reduction in LDL-C concentrations. The findings from AFCAPS/TexCAPS underscored the prophylactic efficacy of lovastatin in individuals exhibiting normocholesterolemia or only moderate hypercholesterolaemia, thereby establishing that, even in the absence of clinically apparent cardiovascular pathology, statin administration can deliver substantial cardioprotective benefit.

Fluvastatin was subjected to rigorous evaluation within the framework of the Lescol Intervention Prevention Study (LIPS), a multicentre, randomized investigation enrolling patients who had recently been subjected to percutaneous coronary intervention (PCI) [42]. In this trial, fluvastatin administration effected a 20–25% decrement in LDL-C concentrations and was associated with a statistically robust 22% relative risk attenuation in major adverse cardiac events (MACE). The clinical relevance of these findings is particularly pronounced in the post-PCI setting, wherein intensive lipid-lowering pharmacotherapy serves as a critical determinant in curtailing the likelihood of recurrent ischaemic episodes in a population with heightened vascular vulnerability. The performance of fluvastatin in this context consolidates its role as a cornerstone of secondary prevention, particularly in individuals undergoing recent coronary revascularisation procedures.

Pravastatin has been rigorously appraised across multiple landmark, randomized, controlled investigations, notably the West of Scotland Coronary Prevention Study (WOSCOPS) [7], the Cholesterol and Recurrent Events (CARE) trial [3], and the Long-Term Intervention with Pravastatin in Ischemic Disease (LIPID) study [4]. In WOSCOPS, a cohort of 6595 male participants with elevated LDL-C concentrations and no antecedent myocardial infarction exhibited a ~26% decrement in LDL-C accompanied by a 31% relative risk attenuation in incident coronary events under pravastatin therapy. The CARE trial, enrolling patients with prior myocardial infarction and moderate baseline cholesterol levels, recorded a 24% reduction in the recurrence of coronary events with pravastatin intervention. The LIPID study, encompassing 9014 subjects with a history of myocardial infarction or unstable angina, corroborated these outcomes, documenting a 24% relative risk reduction in MACE over a 16-year longitudinal follow-up period. Collectively, these datasets substantiate the efficacy of pravastatin in both primary and secondary prevention paradigms, conferring cardioprotection across a broad continuum of baseline cardiovascular risk profiles.

Rosuvastatin, distinguished by its pronounced potency in reducing LDL-C concentrations, underwent pivotal evaluation in the Justification for the Use of Statins in Prevention: an Intervention Trial Evaluating Rosuvastatin (JUPITER) [6]. This large-scale, randomized investigation enrolled 17,802 individuals without overt hyperlipidemia but exhibiting elevated high-sensitivity C-reactive protein (hs-CRP) concentrations. Rosuvastatin therapy yielded a 50% diminution in LDL-C and a 37% reduction in hs-CRP, accompanied by a 44% relative risk attenuation in major cardiovascular events, encompassing myocardial infarction, cerebrovascular accident, and coronary revascularisation. The JUPITER trial was paradigm-shifting in its implications, as it underscored the utility of statin therapy in a cohort lacking conventional dyslipidemic indications, thereby broadening the conceptual framework of statins to encompass anti-inflammatory as well as cardioprotective mechanisms in primary prevention.

Pitavastatin, a newer statin, has shown promise in several trials, though it has not been as extensively studied as some of the other statins. It has been noted for its strong LDL-C lowering effect with a relatively low risk of increasing blood glucose levels, making it a potential choice for patients at risk of diabetes [43,44]. The PEARL and JAPAN-ACS trials have demonstrated its efficacy in reducing LDL-C and improving endothelial function, though more extensive trials are needed to fully establish its long-term cardiovascular benefits [45,46].

Cerivastatin, although exhibiting pronounced potency in lipid-lowering efficacy, was ultimately withdrawn from clinical use following accumulating reports of severe myotoxicity, most notably rhabdomyolysis [28,47]. The clinical development trajectory and post-marketing pharmacovigilance data revealed substantial LDL-C reductions; however, these benefits were counterbalanced by an unacceptably adverse safety profile. The cerivastatin experience serves as a critical exemplar of the necessity for rigorous safety surveillance in statin pharmacotherapy and underscores the delicate equilibrium between therapeutic efficacy and the potential for serious, treatment-limiting toxicities.

In summation, the aggregated corpus of evidence derived from an array of large-scale, randomized, controlled evaluations encompassing multiple statin pharmacophores substantiates their capacity to effectuate substantive reductions in LDL-C concentrations and concomitantly attenuate the incidence of adverse cardiovascular endpoints. Collectively, these trials delineate the extensive therapeutic latitude of statin pharmacotherapy across heterogeneous patient strata, encompassing individuals with and without antecedent cardiovascular pathology, spanning a continuum of baseline lipid phenotypes, and incorporating diverse comorbid constellations. A unifying observation emergent from these datasets is the reproducible decrement in LDL-C burden and the attendant diminution in cardiovascular event rates, findings that have irrevocably entrenched statins as foundational agents within the armamentarium of preventive cardiology. While inter-statin heterogeneity persists in physicochemical attributes, pharmacokinetic disposition, and safety–tolerability spectra, the overarching net clinical benefit in mitigating cardiovascular morbidity and mortality remains unequivocal. This formidable evidentiary framework continues to underpin the pervasive clinical deployment of statins, informed by nuanced, individualized risk stratification and tailored to patient-specific therapeutic exigencies.

## 3. Anti-Inflammatory Mechanisms of Statins

The multifaceted immunoregulatory attributes of statins have been subjected to exhaustive interrogation, with an extensive corpus of in vitro and in vivo experimentation delineating their modulatory repercussions across disparate pro-inflammatory signalling architectures. These distal sequelae are precipitated via abrogation of flux through the mevalonate biosynthetic conduit, wherein attrition of isoprenoid derivatives engenders perturbation of lipid-anchorage-dependent spatial disposition and the operational competency of low-molecular-weight GTP-binding modulators such as Rho, Rac, and Ras (Figure 3) [48,49,50]. In the interest of curtailing prolixity, Table 2 enumerates exemplar pre-clinical interrogations appraising the anti-inflammatory capacities of statins within both animal and cell-based milieus, whereas Table 3 subsequently encapsulates translational deployments of these anti-inflammatory capacities in controlled clinical scenarios targeting non-cardiovascular morbidities. The purpose of this segment is to expound the subordinate and collateral mechanistic ramifications of HMG-CoA reductase antagonism, thereby furnishing both corroborative and dialectical perspectives regarding the extension of statin pharmacology into domains exceeding its orthodox cardiovascular prophylactic remit.

### 3.1. Effect on NLRP3 Inflammasome

**Figure 4:**
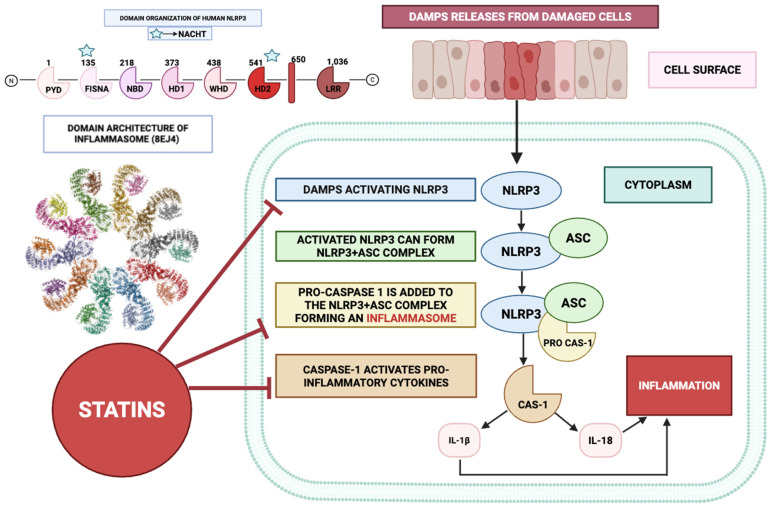
Statins inhibit the NLRP3 inflammasome to attenuate pro-inflammatory cytokine release. This figure highlights the structure of the NLRP3 inflammasome (PDB code 8EJ4), with its associated NACHT domain, which acts as a molecular switch. Stress signals such as DAMPs activate the complex DAMP/NLRP3/CAS-1 axis. Statins inhibit this cascade at multiple points, allowing the attenuation of this pathway towards an anti-inflammatory state.

Figure 4 illustrates the anti-inflammatory mechanisms of statins, particularly focusing on their role in inhibiting the NLRP3 (NOD-, LRR- and pyrin domain-containing protein 3) inflammasome pathway that is crucial for activating pro-inflammatory cytokines. The NLRP3 inflammasome is a multiprotein assembly comprising the pattern-recognition receptor NLRP3, the adaptor molecule ASC (apoptosis-associated speck-like protein containing a caspase recruitment domain), and the effector protease caspase-1. In the context of inflammatory insult, cellular injury provokes the liberation of damage-associated molecular patterns (DAMPs), which engage and prime the NLRP3 scaffold. Its activation may be instigated by a broad spectrum of upstream triggers, encompassing microbial pathogens, crystalline particulates, perturbations in metabolic homeostasis, and hyperglycemic states. Following activation, the NLRP3 sensor undergoes conformational rearrangements that permit high-affinity engagement with the adaptor ASC. This interaction nucleates the formation of a supramolecular signalling platform, enabling the proximity-induced autocatalytic conversion of pro-caspase-1 into its enzymatically active form [124,125,126].

Subsequently, the catalytically active form of caspase-1 proteolytically cleaves the zymogen precursors of interleukin-1β (IL-1β) and interleukin-18 (IL-18), thereby converting them from their latent pro-cytokine states into biologically active, mature forms. These cytokines are potent mediators of inflammation, playing key roles in the pathogenesis of various inflammatory diseases, including atherosclerosis, RA, and metabolic syndrome [127,128]. In particular, IL-1β is a critical mediator of fever and local inflammation, whereas IL-18 stimulates the production of interferon-gamma (IFN-γ) and promotes the differentiation of T-helper cells. Figure 4 shows how the inflammasome complex, upon activation, leads to the release of these cytokines, culminating in an inflammatory response.

Figure 4 illustrates that statins inhibit the activation of the NLRP3 inflammasome at multiple levels, thus preventing the downstream inflammatory cascade. This inhibition can occur through several mechanisms. By suppressing the mevalonate biosynthetic pathway, statins curtail the generation of isoprenoid derivatives—farnesyl pyrophosphate (FPP) and geranylgeranyl pyrophosphate (GGPP)—which serve as essential lipid moieties for the post-translational prenylation of small GTP-binding proteins, including members of the Ras, Rho, and Rac families. By reducing the availability of these intermediates, statins effectively disrupt the proper functioning of these GTPase proteins, thereby inhibiting the assembly and activation of the NLRP3 inflammasome.

The nuclear factor-kappa B (NF-κB) signalling axis serves as a pivotal transcriptional regulator in inflammation, orchestrating the expression of genes for pro-inflammatory mediators such as IL-1β and IL-18. Evidence indicates that statins can attenuate NF-κB activity by impeding the nuclear translocation of its heterodimeric complex, thereby limiting downstream pro-inflammatory gene expression [129,130]. This inhibition reduces the transcription of IL-1β and IL-18, thereby limiting the substrates available for caspase-1 activation [131,132].

Oxidative stress serves as a strong trigger for NLRP3 activation. Statins possess antioxidant properties that can reduce the production of reactive oxygen species (ROS). By lowering oxidative stress, statins prevent ROS-mediated stimulation of the NLRP3 inflammasome, thereby decreasing the secretion of IL-1β and IL-18 [131].

### 3.2. Effect on the NF-κB Pathway

**Figure 5:**
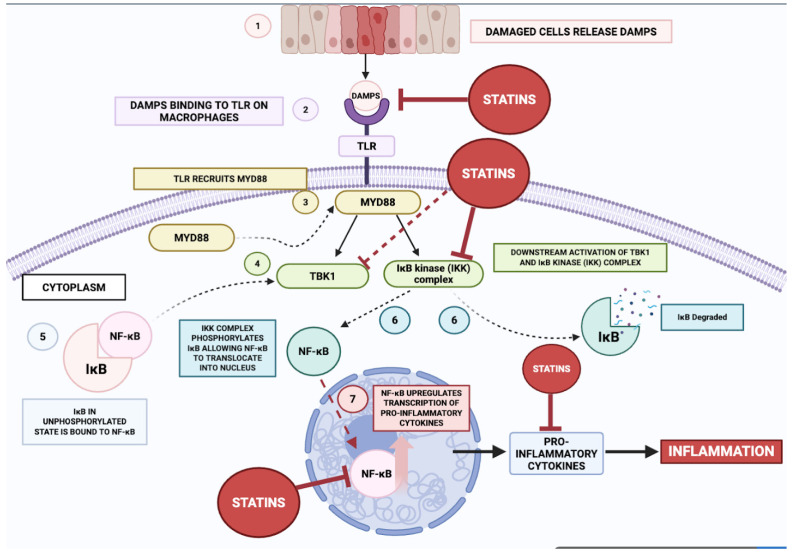
Statins attenuate DAMP-stimulated NF-κB activation and subsequent inflammation. This figure highlights how DAMPs (1) (released from damaged cells due to pathogens, metabolic stress, and other pathological states) activate the NF-κB signalling cascade (7) by binding to TLRs (2) on macrophages, leading to the transcription of pro-inflammatory cytokines (3) (4) (5) (6). Statins exert its inhibitory effects at multiple points in this cascade, thus exerting potent multi-target, anti-inflammatory effects that can be leveraged by the clinical community for therapeutic potentials in patients.

Figure 5 depicts the sequence of molecular events through which damage-associated molecular patterns (DAMPs) initiate NF-κB activation, followed by the anti-inflammatory effects exerted by statins. The process commences when DAMPs, released from injured or stressed cells, are detected by Toll-like receptors (TLRs) expressed by macrophages. The binding of DAMPs to TLRs triggers a signalling cascade involving several key proteins, ultimately resulting in the activation of the NF-κB pathway [131,133,134,135]. Following the detection of danger-associated molecular patterns (DAMPs), Toll-like receptors (TLRs) engage the adaptor molecule myeloid differentiation primary response 88 (MyD88). This adaptor functions as a central signalling nexus, propagating downstream cascades that culminate in the activation of Tank-binding kinase 1 (TBK1) and the IκB kinase (IKK) complex. The IKK complex phosphorylates IκB, an inhibitory protein that binds to NF-κB dimers in the cytoplasm, preventing their translocation to the nucleus. Phosphorylation of IκB leads to its ubiquitination and subsequent degradation, releasing NF-κB [135,136].

Upon liberation from cytoplasmic sequestration, NF-κB translocates to the nuclear compartment, where it engages cognate κB consensus motifs within genomic DNA, thereby instigating transcriptional programmes encoding pro-inflammatory cytokines including TNF-α, IL-6, and IL-1β. These effector molecules constitute pivotal immunopathological mediators, orchestrating the initiation and propagation of inflammatory cascades implicated in the aetiopathogenesis of diverse disorders, notably cardiovascular disease and atherogenesis. The biosynthesis and extracellular liberation of these cytokines facilitate chemotactic recruitment and activation of additional leucocytic populations, thereby amplifying the inflammatory milieu and exacerbating parenchymal injury and pathological progression.

Statins attenuate such pro-inflammatory signalling through multipoint interference within the NF-κB activation axis. Primarily, they impede the catalytic competency of the IKK complex, forestalling the phosphorylation-induced proteasomal catabolism of IκB [129,137]. This preservation of the cytoplasmic NF-κB–IκB complex precludes nuclear ingress of NF-κB, thereby abrogating transcriptional induction of pro-inflammatory gene networks. By interdicting this rate-limiting event, statins effectuate a net suppression of pro-inflammatory cytokine biosynthesis.

Additionally, statins disrupt MyD88-dependent signal transduction [138] via diminution of isoprenoid intermediates—FPP and GGPP—whose availability is requisite for the post-translational prenylation and functional competence of small GTPases operating within this pathway. This biochemical attrition compromises the downstream transductional fidelity of TLR-initiated signalling, resulting in attenuated activation of TBK1 and IKK and, consequently, diminished NF-κB activation.

Beyond these canonical effects, statins mitigate the liberation of DAMPs from structurally compromised cells through enhancement of membrane stability and cytoprotection against cell death. Attenuation of extracellular DAMP release curtails TLR engagement and subsequent pro-inflammatory signalling, thereby diminishing the priming stimuli for innate immune activation.

Cumulatively, these mechanisms culminate in downmodulation of pro-inflammatory cytokines, adhesion molecules, and ancillary inflammatory mediators, leading to reduced immune cell ingress and activation at inflamed loci. This decrement in immunopathological burden translates to mitigated tissue injury and improved clinical endpoints in pathologies such as atherosclerosis and cardiovascular disease. Inhibitory modulation of NF-κB signalling by statins thus constitutes a principal molecular axis through which these agents confer anti-inflammatory and vasculoprotective efficacy.

### 3.3. Comparative Integration of NF-κB and NLRP3 Signalling Under Statin Modulation

The NF-κB pathway and the NLRP3 inflammasome are closely interconnected yet functionally distinct components of the innate inflammatory response. NF-κB plays an upstream role in transcribing the precursor forms of IL-1β and IL-18, while NLRP3 mediates their proteolytic maturation via caspase-1 activation. This two-step inflammatory cascade ensures tight control over the release of potent pro-inflammatory cytokines, preventing excessive tissue damage while maintaining effective immune responses [139].

Statins exert inhibitory effects on both pathways through shared upstream mechanisms, including blockade of isoprenoid synthesis and reduced ROS generation. By inhibiting HMG-CoA reductase, statins prevent the synthesis of FPP and GGPP, crucial isoprenoids required for the posttranslational modification of small GTPase proteins such as Ras, Rho, and Rac1. These proteins are essential for membrane localization and activation of downstream signalling cascades that regulate both NF-κB activation and NLRP3 inflammasome assembly [140].

The depletion of GGPP by statins disrupts Rac1-mediated NADPH oxidase activity, leading to reduced ROS generation. This is particularly significant because ROS serve as both direct activators of NLRP3 through thioredoxin-interacting protein (TXNIP) dissociation and indirect promoters of NF-κB activation through IκB degradation. Furthermore, statins activate the pregnane X receptor (PXR), which directly inhibits NF-κB binding to NLRP3 gene promoter regions, providing an additional mechanism for dual pathway suppression [129].

However, the relative dominance of NF-κB versus NLRP3 signalling may vary by disease context. In chronic vascular inflammation, such as atherosclerosis, NF-κB appears to be more prominent in driving sustained inflammatory gene expression, including adhesion molecules (VCAM-1, ICAM-1), chemokines, and cytokine precursors. Statins effectively reduce these inflammatory markers through multiple NF-κB inhibitory mechanisms, including stabilization of IκB-α, prevention of p65 nuclear translocation, and activation of anti-inflammatory transcription factors like KLF4 [141].

Conversely, NLRP3 plays a greater role in metabolic or acute inflammasome-driven tissue injury, particularly in diabetes mellitus and its complications. In diabetic conditions, hyperglycemia acts as a damage-associated molecular pattern (DAMP) that directly activates NLRP3, leading to IL-1β maturation and subsequent insulin resistance and β-cell dysfunction. Studies demonstrate that NLRP3 expression levels in peripheral blood mononuclear cells and plasma IL-1β concentrations are significantly elevated in diabetic patients and correlate with carotid intima-media thickness, a marker of atherosclerotic progression [142].

Understanding the differential sensitivity of these axes to various statin types and doses remains an area for future translational research. Lipophilic statins (simvastatin, atorvastatin, fluvastatin) demonstrate superior anti-inflammatory effects compared to hydrophilic statins (pravastatin), likely due to enhanced cellular penetration and more effective isoprenoid depletion. High-dose atorvastatin therapy has shown particularly robust effects on both NF-κB and NLRP3 pathways, with significant reductions in IL-6, NLRP3, and STAT3 levels, alongside increased AMPK activation, which promotes autophagy and inflammasome clearance [143,144].

The dual inhibition of these pathways by statins may therefore explain their broad anti-inflammatory utility across atherosclerosis, diabetes, and certain cancers. In coronary microembolization studies, rosuvastatin demonstrated cardioprotective effects through NLRP3 inflammasome inhibition, reducing pyroptotic cell death and preserving mitochondrial function [145]. Similarly, in diabetic atherosclerosis models, statin therapy reduced intrapancreatic macrophage infiltration and cytokine production, correlating with decreased acinar-to-ductal metaplasia formation, a precursor to pancreatic cancer development [146].

The mechanistic convergence of NF-κB and NLRP3 inhibition by statins also provides a molecular rationale for their pleiotropic effects beyond cardiovascular protection, including neuroprotection, cancer prevention, and metabolic disease management. However, the precise balance between these pathways and their relative contributions to different disease states require further investigation to optimize statin selection and dosing strategies for specific inflammatory conditions [146,147].

### 3.4. Effect on the MAPK Pathway

**Figure 6:**
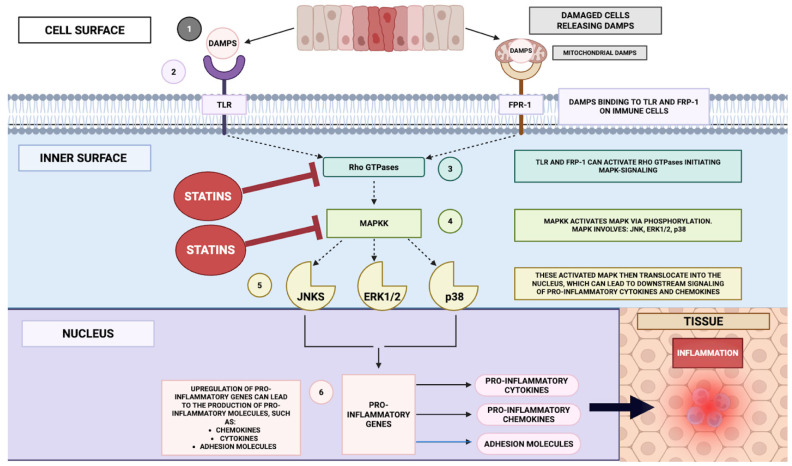
Statins modulate DAMP-induced MAPK signalling to attenuate inflammation. This figure illustrates how DAMPs (1), through binding TLRs (2), can also activate key MAPK families (ERK1/2, JNK, p38) (5) in immune cells, leading to upregulation of pro-inflammatory cytokines and chemokines. Statins exert anti-inflammatory effects primarily by inhibiting the mevalonate pathway, which reduces GTPase prenylation (3) and subsequently dampens MAPK activation (4), thereby attenuating all downstream signalling and inflammatory outcomes (6) as shown above.

Figure 6 schematically delineates the anti-inflammatory modulatory interface of statins as mediated through the mitogen-activated protein kinase (MAPK) signalling architecture. Analogous to the NF-κB cascade, DAMPs serve as proximal activators of principal MAPK subfamilial modules—ERK1/2, JNK, and p38—within innate immune effector populations. Engagement of these kinase axes precipitates phosphorylation-dependent signal propagation culminating in the activation of downstream transcriptional regulators, thereby driving the expression of pro-inflammatory cytokines and chemotactic mediators, including IL-6 [148,149]. Furthermore, DAMP species of mitochondrial provenance have been implicated in the activation of neutrophils via ligation of the formyl peptide receptor-1 (FPR-1), initiating phosphorylation and catalytic potentiation of p39 and ERK1/2, processes requisite for the stimulus-coupled secretion of IL-8 [150].

As delineated previously, statin pharmacodynamics involve suppression of flux through the mevalonate biosynthetic axis, thereby curtailing the generation of isoprenoid intermediates indispensable for the post-translational prenyl conjugation and functional conformational licencing of small GTP-binding proteins. Attenuation of this lipid-modification process constrains downstream mobilization of MAPK subfamilial modules—ERK, JNK, and p38, which constitute pivotal regulatory nodes in the inducible transcriptional programming of pro-inflammatory cytokines and endothelial adhesion determinants within vascular and immunocompetent cell populations. Notwithstanding this canonical suppressive profile, statins under certain experimental or microenvironmental conditions have been observed to potentiate p38 MAPK signalling, a phenomenon capable of priming the NLRP3 inflammasome and augmenting IL-1β secretion from macrophages, thereby exemplifying the context- and lineage-contingent variability of response [151]. The prevailing anti-inflammatory phenotype is principally ascribed to inhibiting Rho-GTPase-governed MAPK activation cascades, culminating in diminished NF-κB pathway throughput. Through the downmodulation of MAPK signalling competency, statins effectuate a broad repression of inflammatory mediator biosynthesis, concomitantly attenuating leucocyte trafficking and endothelial cell activation [48,152,153].

### 3.5. Effect on T-Cell Differentiation

Innate immunological reactivity to pro-inflammatory stimuli entails the ontogenetic progression of thymocyte-derived precursors into naïve CD4^+^ T lymphocytes, which subsequently undergo lineage commitment toward regulatory T cells (T-reg) or T helper 17 cells (Th17) through an intricately orchestrated integration of T cell receptor (TCR)-mediated signalling, cytokine milieu, and a network of cross-modulatory cascades incorporating the MAPK axis, Toll-like receptor (TLR) signalling, and the NF-κB pathway. Within this framework, TCR-initiated signal transduction exerts a pivotal role in dictating T-reg versus Th17 lineage bias, in part through the activity of interleukin-2–inducible T cell kinase (ITK), whose functional predominance favours Th17 specification at the expense of T-reg development [154]. Transforming growth factor-β (TGF-β) constitutes an obligate cue for both lineages, instigating the activation of SMAD3/4 heterotrimeric transcriptional assemblies to promote T-reg commitment via Foxp3 expression, whereas the concomitant presence of IL-6 or IL-21 engages STAT3, which interacts with SMAD3 in a manner that obstructs SMAD3/4 complex assembly, thereby biassing differentiation toward the Th17 lineage via induction of RORγt expression [155,156].

Foxp3-expressing T-regs mitigate inflammatory tone by eliciting anti-inflammatory cytokines such as IL-10 and TGF-β, upregulating inhibitory co-receptors including CTLA-4, and imposing metabolic restrictions upon effector T lymphocytes [155,157]. Furthermore, Foxp3 transcriptionally antagonizes pro-inflammatory drivers such as RORγt, thereby directly suppressing Th17 ontogeny and effector functionality. Induction of the T-reg phenotype is accompanied by attenuated MAPK and Rho-GTPase signalling relative to Th17 differentiation, a biochemical configuration that contributes to the acquisition of suppressive competence [157]. In the context of T-regs, TLR-dependent signalling can fine-tune suppressive activity; however, hyperactivation of the TLR/MyD88/IKK cascade has the potential to destabilize Foxp3 expression and compromise suppressive efficacy. TANK-binding kinase 1 (TBK1) exerts an indirect yet modulatory influence on T-reg biology via its regulatory intersections with NF-κB and IRF-dependent pathways [158,159].

Conversely, Th17 cells potentiate inflammatory responses through RORγt-directed transcriptional programmes driving IL-17 and IL-22 production [155,157]. The acquisition of the Th17 phenotype necessitates coordinated input from TCR engagement, TGF-β signalling, and IL-6/IL-21-mediated cues, with STAT3 activation serving as the principal transcriptional driver of RORγt induction and activity [158]. IL-6/STAT3 signalling additionally impedes SMAD3/4 complex formation, reinforcing the preferential commitment to Th17 over T-reg fate. In this lineage, the MAPK and Rho-GTPase pathways exhibit heightened activation, thereby sustaining effector functionalities and amplifying cytokine output. Furthermore, TLR/MyD88/IKK signalling within antigen-presenting cells augments IL-6 and IL-23 production, providing an extrinsic cytokine milieu that intensifies Th17 polarization [160,161,162].

**Figure 7:**
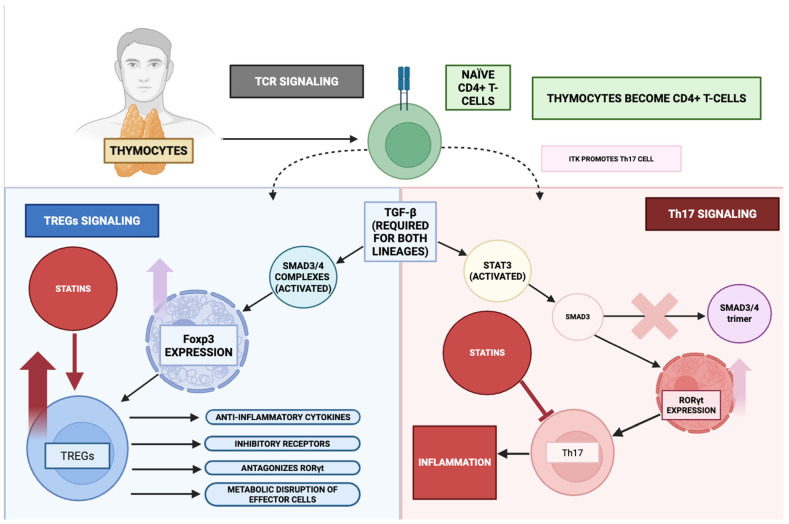
Statins modulate T-cell plasticity towards an anti-inflammatory phenotype. This figure demonstrates the dynamic balance between Tregs and Th17 cells from naïve CD4+ T cells. Anti-inflammatory Tregs (Foxp3 expression) are upregulated via statins’ anti-inflammatory activity. On the other hand, pro-inflammatory Th17 cells (expressing RORγt) are attenuated by statins, thus shifting this balance towards lower inflammatory levels in the body.

Through the above mechanisms, and as summarized in Figure 7, statins increase the frequency of Treg expression while suppressing Th17 differentiation through inhibition of the mevalonate pathway [50,162]. Statins also induce T-reg recruitment to sites of inflammation via CCL1-dependent chemotaxis [163]. Additionally, inhibition of RORγt expression and IL-17 production involves interference with STAT3 and MAPK signalling. As shown in studies investigating models of autoimmune disease and atherosclerosis, this results in a shift in the Th-17/Treg balance towards immune tolerance and reduced inflammation [163,164,165].

### 3.6. Effect on Leukocyte Adhesion and Migration

Leucocyte tethering, arrest, and transendothelial migration represent cardinal phases in both immunosurveillance and inflammatory pathophysiology, unfolding through a sequentially orchestrated multistep paradigm. The initial phase involves selectin-mediated capture and rolling interactions, succeeded by chemokine-triggered activation of integrin conformational states, culminating in firm adhesion via αLβ_2_ (LFA-1) and α_4_β_1_ (VLA-4) integrins, and eventual diapedesis across the endothelial barrier. These processes necessitate highly plastic modulation of integrin affinity–avidity parameters, cytoskeletal remodelling driven by small GTPases such as Rho and Rac, and a coordinated endothelial programme incorporating upregulation of adhesion determinants (e.g., ICAM-1, VCAM-1) alongside junctional architecture reorganization to facilitate leucocyte extravasation [166,167,168].

**Figure 8:**
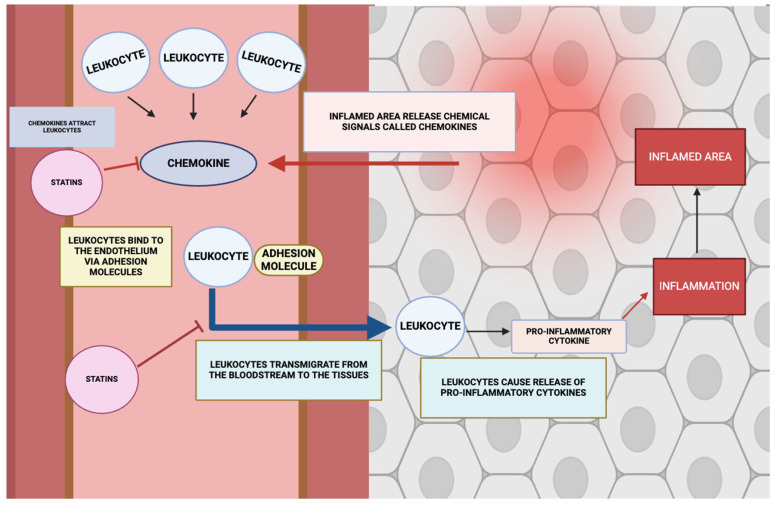
Statins attenuate leukocyte adhesion, transmigration, and inflammation. This figure illustrates the multi-step cascade of leukocyte activity—from adhesion and transmigration to modulating inflammation—during inflammation. Statins inhibit this cascade at multiple levels, by inhibiting chemokines, inhibiting leukocyte transmigration, and overall inhibiting the inflammation observed in the tissues.

As depicted in Figure 8, pharmacological suppression of the mevalonate biosynthetic cascade by statins perturbs the molecular machinery governing leucocyte adhesive and migratory behaviour. Such intervention compromises integrin activation and cytoskeletal dynamism within leucocytes, thereby attenuating adhesion molecule display and diminishing leucocyte–endothelial engagement [168,169]. Concomitantly, statins exert a transcriptional downmodulatory effect on endothelial adhesion molecules—including E-selectin, ICAM-1, and VCAM-1—further constraining immune cell ingress into inflamed parenchyma [168].

Empirical observations indicate heterogeneity in the magnitude of these effects, with lipophilic statins manifesting a more pronounced immunomodulatory profile relative to their hydrophilic counterparts [50,170]. Lipophilic congeners more effectively abrogate Rho GTPase prenyl conjugation, resulting in more profound suppression of adhesion molecule expression, more substantial impairment of integrin activation, and greater restriction of leucocyte migratory competence. Beyond these effects on innate immune trafficking, such agents more potently facilitate T-reg expansion and functional competence while concurrently restraining Th17 differentiation and IL-17 synthesis, via interference with MAPK and STAT3 signalling modules downstream of the mevalonate axis. Collectively, statin-mediated disruption of leucocyte adhesion–migration dynamics contributes to the mitigation of both vascular and systemic inflammatory burden by reducing immune cell infiltration and recalibrating adaptive immune responses.

### 3.7. Effect on Cytokine Production

The principal immunostimulatory mediators central to the inflammatory signalling frameworks described above [170], notably the interleukin-1 family member β-isoform, the pleiotropic glycoprotein interleukin-6, and tumour necrosis factor of the α-subclass—arise from the convergent activation of multiple upstream signal transduction architectures. The β-isoform of interleukin-1, acting through canonical NF-κB signal relay, potentiates the inflammatory cascade by inducing pyrogenic responses, enhancing the transcriptional upregulation of endothelial counter-receptors for immune cell arrest, and facilitating the recruitment and translocation of circulating leukocyte subsets [171,172,173,174]. This mediator further amplifies the network by driving additional soluble immunoregulatory factors—including the α-subclass tumour necrosis factor and interleukin-6—and by supporting polarization toward the T helper 17 phenotype, thus interlinking innate pattern-recognition responses with antigen-specific adaptive immunity [174].

Interleukin-6, whose synthesis is inducible via β-interleukin-1, α-tumour necrosis factor, and integrated NF-κB/MAPK activation, exerts systemic actions that encompass stimulation of hepatocyte-driven acute-phase reactant synthesis, promotion of B-cell terminal differentiation, and reinforcement of Th17 lineage commitment, while concurrently attenuating T-regulatory cell development [175,176]. It constitutes the predominant molecular trigger for hepatic C-reactive protein biosynthesis through STAT3-mediated transcriptional programming, with β-interleukin-1 and α-tumour necrosis factor providing synergistic potentiation of this effect. This β-interleukin-1/interleukin-6/C-reactive protein triad forms a critical inflammatory amplification loop: the β-isoform of interleukin-1 induces interleukin-6 expression via NF-κB- and C/EBPβ-dependent pathways, which in turn drives direct transcriptional activation of the C-reactive protein gene [170,177].

**Figure 9:**
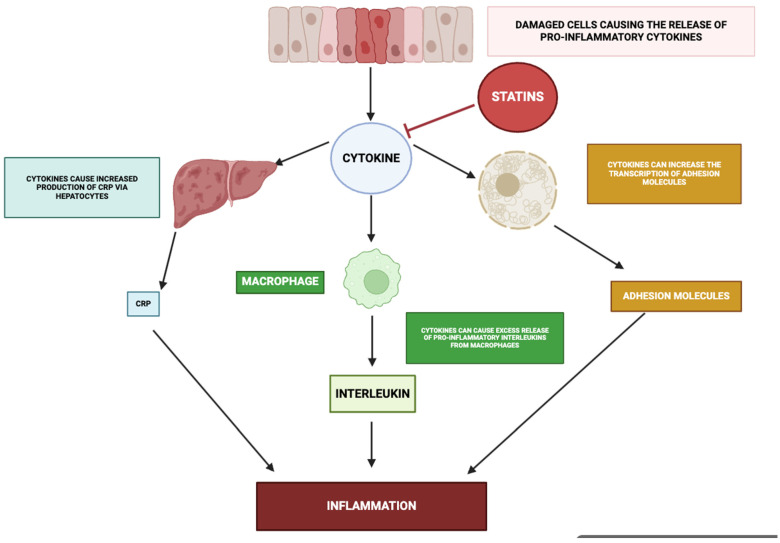
Pro-inflammatory cytokine network and statin-mediated attenuation. This figure illustrates the network of key pro-inflammatory cytokines and other inflammatory molecules, such as CRP, and their consequent effects. These effects are inhibited via statins’ anti-inflammatory activity on cytokines, thus attenuating downstream signalling.

The α-subclass tumour necrosis factor, generated by myeloid and T-lineage cells following activation of NF-κB and MAPK modules, engages its cognate receptors to further propagate NF-κB-dependent transcription, initiate programmed cell death, and induce expression of vascular adhesion ligands (including ICAM-1 and VCAM-1), thereby facilitating immune cell tethering, arrest, and transendothelial passage. In addition, it reinforces the inflammatory network by stimulating the synthesis of β-interleukin-1 and interleukin-6, establishing a reciprocally amplifying cytokine circuit. As elaborated in preceding sections and schematically depicted in Figure 9, statins recalibrate these mediator networks via suppression of mevalonate-derived intermediates, dampening pro-inflammatory effector output and enhancing counter-regulatory anti-inflammatory signalling.

## 4. Statins in Chronic Inflammatory Conditions

Given the anti-inflammatory mechanisms of statins on a molecular and genetic level, several studies have extensively investigated the use of statins in chronic inflammatory conditions. Through in vitro models, in vivo models, and clinic trials, the following sections integrate the underlying inflammatory pathway with the application of statins in the following chronic inflammatory conditions: diabetes, OA, and cancer. Additional sections also review the use of statins in RA and COPD. It is important to note how several studies highlight the anti-inflammatory role of statins independent of their lipid-lowering mechanisms.

### 4.1. Inhibition of Pro-Inflammatory Cytokines

As delineated previously, statin-mediated pharmacological interference attenuates the biosynthetic availability of isoprenoid derivatives, principally farnesyl pyrophosphate (FPP) and geranylgeranyl pyrophosphate (GGPP), whose presence is indispensable for the post-translational lipid conjugation–dependent signalling competency of low–molecular-weight GTP-binding proteins, including members of the RhoA, Rac, and Ras subfamilies [178]. In the context of diabetes, this poses immense clinical relevance, due to its hallmark state of chronic inflammation and metabolic stress [179,180]. This stress can impair the normal signalling of these small GTPases, thus implicating them in the pro-inflammatory state and insulin resistance in metabolically relevant tissues—vascular endothelium, adipocytes and skeletal muscles. For instance, RhoA is implicated in oxidative stress and increased vascular permeability, contributing to diabetic vascular complications [181,182]. Moreover, Rho GTPases induce the expression of inflammatory adipokines in adipocytes [183], thus further promoting an inflammatory state and insulin resistance in the diabetic patient. In addition, Rac1 dysfunction leads to insulin resistance in skeletal muscles via attenuated GLUT4 transport and glucose uptake [184]. As such, the administration of statins would allow the aberrant activity of these signalling molecules/pathways to be attenuated via the inhibition of the mevalonate pathway. 

Multiple investigative efforts have elucidated the proximate molecular modalities through which statins perturb small GTPase functionality. In this context, Tanaka et al. reported that both atorvastatin and pitavastatin potentiate the transcriptional and/or translational output of small GTP-binding protein GDP dissociation stimulator (SmgGDS), a chaperoning entity that orchestrates proteasome-dependent catabolism of the Rac1 isoform within endothelial nuclei, thereby facilitating its turnover and functional attenuation [185]. Vecchione et al. demonstrated that atorvastatin mitigates reactive oxygen species (ROS) accrual within human aortic endothelial cells (HAECs) by constraining the pathologically dysregulated activation state of Rac1 [186]. Concordantly, Bruder-Nascimento et al. documented that atorvastatin induces a substantive downmodulation of RhoA abundance in murine models of diabetes mellitus, specifically within the pathophysiological milieu of diabetic nephropathy [187].The chronic inflammatory environment in diabetic patients is in part facilitated by the NF-κB cascade [188], which is triggered by free fatty acid (FFA)-induced inflammation and glucolipotoxicity (increased glucose and lipid levels) and lead to beta islet cell dysregulation [189]. NF-κB drives the transcription of the inflammatory molecules TNF-α and IL-6, which facilitates the dysfunction in Type 2 Diabetes (T2D) [190]. In Type 1 Diabetes (T1D), pro-inflammatory cytokines secreted by immune cells within the islets induce NF-κB activation in β-cells, contributing to their auto-induced damage [191]. Furthermore, NF-κB pathway stimulation can result in vascular complications in not only the pancreas, but also in the kidney, heart and retina, hence exacerbating the systemic inflammatory damage in diabetic patients [192]. This inflammatory over-activation translates into severe clinical manifestations such as diabetic nephropathy, cardiomyopathy, and retinopathy.

Statins offer alternative therapeutic avenues that can reduce the incidence of the above adverse outcomes in diabetic patients. One such study by Zhang et al. showed atorvastatin ameliorated nephrotoxicity in streptozotocin-induced diabetic mice via attenuated NF-κB and TNF-α signalling [193]. Similarly, simvastatin showed cardio-protective properties by reducing NF-κB translocation and TNF-α activation in the myocardium of diabetic mice, allowing for reduced hypertrophy and improved cardiac function [194]. Simvastatin’s anti-inflammatory activity is further supported by Lin et al., which showed a downregulation in NF-κB and TNF-α in mice fed a high-fat diet [195].

However, while molecular and mechanistic insights are increasingly robust, effective clinical translation remains limited by an incomplete understanding of how these biochemical pathways interact with patient-level inflammatory and metabolic risk. Recent clinical evidence highlights that low-grade inflammation and glycemic dysregulation, frequent in individuals with familial hypercholesterolemia (FH) or metabolic syndrome, not only shape the pattern of atherosclerotic damage but also modulate the efficacy of lipid-lowering therapies such as statins, independent of achieved LDL-C levels. For example, the referenced study by Di Giacomo Barbagallo et al. demonstrated that among genetically defined FH patients, those with non-LDL receptor mutations exhibited worse glycemic control, higher fasting plasma glucose and HbA1c, and greater peripheral atherosclerotic plaque burden compared to those with LDL-receptor mutations, even though LDL-C levels were higher in the LDLR mutation group. Notably, glycemic profile and inflammatory state were associated with distinct distributions of atherosclerotic lesions, namely, high glycemic status and insulin resistance correlated with greater peripheral plaque, whereas higher lifelong LDL-C exposure in certain genotypes was linked to more pronounced coronary artery calcification [196].

These findings underscore that glycemic abnormalities and chronic low-grade inflammation act as independent drivers of vascular injury, and their interplay can attenuate or modify the vascular benefits of statin therapy beyond simple LDL-C reduction. Evidence indicates that measures such as the triglyceride-glucose (TyG) index are emerging as valuable markers connecting dysglycemia [197], insulin resistance, and circulating inflammatory mediators to early atherosclerotic changes. Therefore, optimal risk stratification and evaluation of statin efficacy should move beyond traditional lipid endpoints to incorporate assessment of inflammatory biomarkers (e.g., hs-CRP, interleukin-6, TNF-α) and detailed patient-specific metabolic profiling. This approach could identify patients at “residual inflammatory risk” who may benefit from adjunctive or alternative anti-inflammatory therapies and ultimately provide a more tailored prevention strategy for high-risk groups including FH and those with metabolic dysfunction [196].

There have been many studies that have highlighted the pleiotropic anti-inflammatory action of statins independent of the drug’s primary lipid-lowering mechanism that can be extended in inflammatory-driven conditions such as OA. For instance, two separate Mendelian randomization studies demonstrate the protective effects of atorvastatin, rosuvastatin, and simvastatin on hip and knee OA [198,199]. As such, these have given researchers ground for investigating the role statins can play in the management of OA. The chronic inflammatory processes underlying the disease progression in OA are primarily orchestrated by cytokines such as IL-1β, TNF-α, and IL-6 that are produced by activated chondrocytes, synovial fibroblasts, and infiltrating immune cells [200]. These pro-inflammatory mediators orchestrate the propagation of a catabolic synovial milieu, wherein the augmented transcriptional activation of matrix metalloproteinases (MMPs), concomitant attenuation of anabolic extracellular matrix biosynthesis, and sustained synovial inflammatory signalling synergistically exacerbate joint tissue degeneration [201]. Notably, the catabolic bioactivity of IL-1β encompasses the induction of programmed chondrocytic cell death, the suppression of proteoglycan biosynthetic pathways, and the upregulation of secondary pro-inflammatory mediator cascades, collectively engendering a self-perpetuating degenerative circuit that accelerates articular cartilage attrition [202].

Statins’ benefit in directly suppressing these cytokines has been shown in a study using porcine cartilage explant models that were designed to mimic OA [53]. Treatment with atorvastatin was shown to significantly inhibit the release of TNF-α induced by IL-1β stimulation. Another study investigating the effects of simvastatin in fibroblast-like synoviocytes (FLS), a cell type involved in synovitis associated with RA and OA, demonstrated dose-dependent inhibition of IL-6 and IL-8 production [203]. This inhibitory effect was maintained even when the FLS were stimulated with IL-1β [204]. Additionally, the inhibitory effects of simvastatin on IL-6 and IL-8 were reversed by co-incubation with mevalonic acid (specifically GGPP), while the addition of FPP failed to reverse the inhibition. This indicates that simvastatin can counteract the effects of pro-inflammatory signals and mediate anti-cytokine effects through depletion of GGPP, independent of the FPP-dependent branches of the mevalonate pathway. This elucidates a testable mechanism for the pleiotropic anti-inflammatory effects of statins and suggests that the potency of different statins may correlate with their ability to inhibit GGPP synthesis in target joint cells.

Persistent, dysregulated inflammatory signalling constitutes a central oncogenic driver, with diverse neoplastic lineages exhibiting a sustained elevation of immunomodulatory mediators that potentiate malignant progression, metastatic dissemination, and therapeutic refractoriness. In colorectal carcinoma (CRC), pathognomonic amplification of tumour-promoting immunocytokine networks—exemplified by the overabundance of tumour necrosis factor superfamily ligands, IL-1 family members, pleiotropic IL-6–type glycoproteins, and Th17-associated effector cytokines—propels disease evolution. Contemporary pharmacological strategies aimed at curtailing malignancy-associated inflammation have interrogated discrete nodal points within these signalling axes; for instance, antagonism of the IL-1 receptor with monoclonal agents such as canakinumab has demonstrated efficacy in attenuating lung cancer incidence and mortality [205]. Mechanistic interrogation by Sun et al. revealed that inflammatory mediator–driven repression of tumour-suppressive microRNAs, notably miR-615-5p, derepresses oncogenic transcripts such as *STC1*, thereby expediting tumour proliferative kinetics [206]. A prospective cohort by Florescu et al. delineated progressive stage-dependent escalation in circulating concentrations of IL-1–related, IL-6–related, and TNF-associated proteins in CRC, with IL-6 exhibiting the steepest trajectory [207], findings corroborated by a meta-analysis identifying IL-6 signalling as a prognostic correlate of augmented tumour burden and diminished survival [208]. Analogous immunopathological patterns have been documented in breast malignancies, wherein IL-17A orchestrates a pro-tumorigenic cytokine-chemokine cascade—including IL-6, TGF-β, IL-1 isoforms, IL-8, TNF ligands, CXCL1, and CCL2—to reinforce a tumour-supportive stromal niche [209,210]. Elevated IL-1 family signalling has likewise been associated with advanced disease and therapeutic non-responsiveness in breast carcinoma [211,212]. In pulmonary neoplasia, hyperactivation of inflammatory mediator production by tumour-associated alveolar macrophages yields marked surges in IL-6– and IL-1–related factors both locally and systemically, correlating with adverse clinical trajectories [213,214,215]. Cumulatively, these oncological datasets converge on the conclusion that dysregulated cytokine networks sculpt a microenvironment conducive to malignancy. In this context, the pleiotropic immunomodulatory properties of HMG-CoA reductase inhibitors have garnered considerable investigative interest, particularly their capacity to attenuate principal cytokine-driven signalling circuits.

Despite early concerns regarding a potential increase in cancer incidence among statin users, recent research show that statins reduce cancer risk by 20–28%, and also lower the likelihood of recurrence in prostate cancer patients post-radical prostatectomy [216]. Experimental evidence indicates that simvastatin exerts a pronounced suppressive effect on neoplastic cell–elicited pro-inflammatory immunocytokine release—specifically attenuating IL-1 family β-isoform and type II interferon output—while concomitantly restraining the proliferative kinetics of colorectal carcinoma cells [217,218]. In vitro experiments further support this, showing that simvastatin reduces IL-6 and IL-8 levels in colorectal cancer cells [218]. In the context of breast cancer, statins have been extensively researched for their ability to inhibit pro-inflammatory cytokines. A comprehensive review conducted by Dang et al. outlines several mechanisms by which statins, especially lovastatin, regulate tumour progression, notably through the suppression of inflammatory cytokine signalling via the LKB1–AMPK–p38–MAPK–p53 pathway [219]. Supporting this, Liu et al. demonstrated that treatment with simvastatin has been shown to inhibit protein prenylation in breast cancer cells, leading to decreased IL-6 secretion and reduced proliferation of senescent conditioned media on breast cancer cells [220]. In lung cancer, simvastatin has demonstrated comparable potential. Treatment of mesenchymal stromal cells (MSCs) isolated from lung cancer patients led to a significant reduction in the production of IL-6 and chemokines like CCL2, and CCL3 that play key roles in tumour progression and immune modulation [221]. Moreover, simvastatin has been shown to inhibit the NF-κB pathway, leading to suppression of IL-8 production, further highlighting its anti-inflammatory potential [222]. Additionally, a study found that both pitavastatin and pravastatin significantly reduced IL-6 and IL-8 expression in LPS-stimulated bronchial epithelial cells, underscoring their potential to modulate cytokine-driven inflammation within the lung TME [223]. Together, the evidence across colorectal, breast, and lung cancer models highlights a consistent anti-cytokine effect for statins. By attenuating IL-1β, IL-6, IL-8 and related chemokines, statins may weaken the inflammatory pathways that sustain tumour growth and immune evasion in cancer and strengthen the efficacy of therapeutic regimens in cancer patients.

To complement the mechanistic discussion and enhance translational clarity, we provide below a concise summary of clinical studies evaluating the effects of various statins on key inflammatory biomarkers and cancer-related outcomes across different patient populations. This synthesis highlights the heterogeneity in statin responses and underscores their emerging adjunctive potential beyond lipid lowering (Table 4).

Beyond statins, emerging lipid-lowering therapies that also exhibit anti-inflammatory or metabolically neutral profiles are gaining traction, particularly in patient populations with comorbid cardiovascular, metabolic, and neoplastic conditions. One notable example is inclisiran, a small interfering RNA (siRNA) that targets hepatic PCSK9 synthesis. Unlike traditional statins, inclisiran lowers LDL-C by enhancing LDL receptor recycling in hepatocytes, utilizing a unique RNA interference mechanism that allows for infrequent (biannual) dosing and may improve adherence over long-term therapy [236]. Crucially, recent clinical data highlight that inclisiran not only offers robust LDL-C, lowering efficacy, with reductions consistently around 50%, but also sustains a neutral or potentially favourable profile regarding glycometabolic and inflammatory parameters. Large-scale phase III trials (such as ORION-9, -10, and -11) and real-world studies report an absence of glycemic perturbations or negative impacts on glucose metabolism, a concern that can affect statins in predisposed patients. Similarly, inclisiran does not appear to induce pro-inflammatory changes, and its hepatic specificity minimizes off-target effects that might be problematic in patients with metabolic syndrome, diabetes, or cancer-associated dysmetabolism [237].

These characteristics render inclisiran an attractive adjunct or alternative to statin therapy, especially in high-risk subgroups for whom inflammation, metabolic imbalance, and cancer biology are closely intertwined. As ongoing research continues to evaluate the long-term cardiovascular outcomes and immunometabolic effects of inclisiran, there is growing interest in its potential synergy with statins, not only for complementary LDL-C lowering, but also for co-modulation of immunometabolic pathways that underlie complex cardiovascular and oncologic comorbidities. Future studies should further delineate the benefits of combining inclisiran with statins and anti-inflammatory agents to optimize risk reduction in these especially vulnerable patient populations [237].

### 4.2. Modulation of Immune Cell Phenotypes

The dynamic modulation and expression of immune cell phenotypes, particularly the plasticity of T helper cells, Treg, and macrophage subsets, are central to the pathogenesis and treatment of chronic inflammatory diseases. Aberrant immunophenotypic plasticity, manifested as maladaptive skewing from anti-inflammatory/tolerogenic lineages (e.g., Foxp3^+^ regulatory T cells, alternatively activated M2 macrophages) toward pro-inflammatory/effector subsets (e.g., IFN-γ–dominant Th1, IL-17–producing Th17, classically activated M1 macrophages), perpetuates a state of unresolved inflammation and collateral tissue injury. Contemporary immunotherapeutic paradigms increasingly focus on recalibrating these lineage-differentiation trajectories to re-establish immunological equilibrium and facilitate resolution of chronic inflammatory pathology.

Another crucial aspect of diabetic pathology is the modulation of immune cell phenotypes. Diabetic patients tend to present with a pro-inflammatory immune cell phenotype, which allows for the perpetual inflammatory-mediated damage and loss of repair in the affected tissues [238]. One such example is Th1 lymphocytes and their pro-inflammatory cytokines TNF-α and IFN-γ [239]. Consequently, by inhibiting the prenylation and functional activation of G-proteins as discussed before, statins can potentially dampen TCR signalling and lead to a significant reduction in the secretion of these Th1-associated inflammatory mediators, thereby inhibiting the overall Th1 immune response. In T2D, elevated levels of TNF-α and IFN-γ play a major role in causing insulin resistance by disrupting insulin signalling pathways and promoting pancreatic beta-cell dysfunction [240]. A predominance of Th2-skewed immune polarization may confer a cardiometabolic advantage, as evidenced by the findings of Madhumita et al. [241], wherein individuals with diabetes mellitus and concomitant coronary artery pathology exhibited a disproportionately augmented Th1 cytokine milieu concomitant with an attenuated Th2-associated secretome. Coward and Chow [242] demonstrated in a cohort of immunologically healthy volunteers that atorvastatin attenuated the prevalence of Th1-effector cytokine–producing lymphocytes, predominantly IFN-γ and IL-2-secreting subsets, while concomitantly skewing the immune axis toward a Th2-biassed cytokine profile, typified by augmented IL-4 and IL-10 production. In an independent clinical evaluation encompassing middle-aged and elderly cohorts with established Type 2 diabetes mellitus and coexistent periodontitis, therapeutic administration of atorvastatin precipitated a marked attenuation in pro-inflammatory cytokine burden, evidenced by substantially diminished IL-6 and TNF-α titers within both gingival crevicular exudates and salivary secretions—findings congruent with a localized downmodulation of inflammatory activity. Complementary in vitro analyses revealed that cerivastatin, simvastatin, lovastatin, and atorvastatin each induced a concentration-dependent repression of Th1 lineage commitment (manifested as reduced IFN-γ output) alongside potentiation of Th2 differentiation (elevated IL-4 production) in murine T lymphocytes, with cerivastatin displaying the most potent immunopolarizing efficacy. Importantly, this Th1→Th2 repolarization was abrogated upon exogenous mevalonate supplementation, implicating canonical blockade of the mevalonate-isoprenoid biosynthetic axis as the mechanistic substrate for these immunomodulatory effects [243].

Attenuation of a Th1-skewed immunological milieu has also been documented within the context of Type 1 diabetes mellitus (T1D). In the TCR-HA/RIP-HA double-transgenic (dTg) murine model of autoimmune diabetes, oral administration of atorvastatin elicited a marked immunophenotypic reorientation: splenic lymphocyte populations in “protected” atorvastatin-treated mice exhibited a pronounced Th2-dominant signature, in contrast to the overt Th1 predominance observed in untreated diabetic counterparts [244]. Quantitative RT-PCR profiling revealed significant transcriptional upregulation of canonical Th2-associated transcription factors—STAT6, c-MAF, and GATA3—in the atorvastatin-protected cohort. In contrast, diabetic controls displayed elevated expression of Th1-defining regulators STAT4 and T-bet. Complementary findings by Espinosa-Carrasco et al. corroborated the immunomodulatory capacity of statins, demonstrating a reduction in pathogenic CD8^+^ T-cell infiltration into pancreatic islets [245]. The evidence delineates a role for statin-driven immunomodulation in attenuating autoreactive effector T-lymphocyte functionality, suppressing the transcriptional and translational programmes underpinning pro-inflammatory cytokine synthesis, and consequently forestalling β-cell attrition. By orchestrating a shift in CD4^+^ T-cell lineage commitment away from a Th1-dominant axis toward a more immunoregulatory Th1/Th2 equilibrium, such pharmacological intervention disrupts the feed-forward inflammatory circuitry that perpetuates the autoimmune milieu in T1D.

When it comes to inflammation, macrophages are among the first immune cells at the site of the inflammatory response to initiate, signal other inflammatory cells, and to regulate the environment according to the body’s requirements. In immunologically quiescent states, monocyte-derived macrophages retain a high degree of phenotypic malleability, transitioning bidirectionally along the polarization continuum between classically activated (M1) pro-inflammatory effector phenotypes and alternatively activated (M2) immunoregulatory phenotypes [124]. In the context of diabetic pathology, however, this polarization axis becomes skewed toward sustained M1 predominance [246], thereby reinforcing and perpetuating a chronic pro-inflammatory milieu through amplification of cytokine-driven feed-forward inflammatory cascades. In a recent in vitro study conducted by Muffova et al., the human macrophages showed that fluvastatin suppresses pro-inflammatory M1 markers and enhances anti-inflammatory M2 markers [247]. While not necessarily conducted in the disease setting of diabetes, the aspect of M1 polarization phenotype is vital in diabetic pathophysiology.

While discussing the pro-inflammatory phenotype of diabetes, it is important to investigate the anti-inflammatory landscape in diabetes as well, specifically the lack thereof. Due to the overwhelming inflammatory signalling, anti-inflammatory immune cells, such as Treg, are noted to be diminished. In order for successful management of diabetes, it is vital to increase these anti-inflammatory immune cell phenotypes to suppress the pro-inflammatory state and lead to better health outcomes.

Meng et al. reported that simvastatin augments Treg abundance and enhances the intraplaque expression of Foxp3, TGF-β, and IL-10 within atherosclerotic lesions of apolipoprotein E-null (ApoE^−/−^) murine models [248]. Concordantly, atorvastatin exposure in human peripheral blood mononuclear cells (PBMCs) elevated the frequencies of CD4^+^CD25^high^ and CD4^+^CD25^+^Foxp3^+^ subsets, while in hyperlipidaemic patients, simvastatin similarly elicited an upsurge in circulating Treg levels [249]. Downregulation of inflammation in these plaques can lead to reduced endothelial dysfunction and diabetic cardiomyopathy [131,250]. In previous studies, statin-induced Treg upregulation was observed during non-treatment periods. To address the evidentiary gap concerning the immunomodulatory capacity of statins, Rodrigues-Perea et al. performed an in vivo longitudinal intervention in normocholesterolaemic male volunteers, quantifying CD4^+^ T-cell transcript abundance for FOXP3, IDO, TGF-β, and IL-10 over a 45-day regimen, with sampling on days 0, 7, 30, and 45. Both statin treatments elicited a significant expansion in Treg frequency concomitant with elevated FOXP3 mRNA expression [251]. Although the study was conducted in non-diabetic volunteers, it provides robust evidence of statins’ anti-inflammatory activity; thus, further triangulation and experiments must be conducted as well. The findings from these studies, however, can be translated to potential benefits in protection the onset and progression of diabetic microvascular and macrovascular complications.

The chronic inflammatory infiltrate in the synovium of an OA joint is largely populated by macrophages, mostly of M1 phenotype [252], with emerging evidence pointing towards the involvement of adaptive immune cells [253]. Statins’ immunomodulatory effects appear to actively reprogram the functional phenotype of these immune cells towards a more tolerogenic and anti-inflammatory state [254]. While direct investigations of these effects on OA models are pending, data from other disease models show compelling evidence of statins’ effect on macrophage phenotype imbalance. In an experimental rat model of immune-mediated glomerulonephritis, atorvastatin administration not only attenuated overall macrophage infiltration but also promoted a phenotypic reprogramming, evidenced by a diminution of M1-like macrophage subsets characterized by a pro-inflammatory cytokine/chemokine expression profile, alongside an expansion of M2 macrophage populations exhibiting anti-inflammatory, immunoregulatory, and tissue-reparative functional attributes, including IL-10–producing cells [255]. A similar reprogramming effect was observed in a co-culture model simulating the inflammatory environment of periodontal disease, where simvastatin suppressed the inflammatory response of macrophages while upregulating markers associated with tissue homeostasis and the M2 phenotype [256].

Additionally, statins also exhibit modulatory effects on T-lymphocytes as shown in a study on human PBMCs [257]. Both simvastatin and lovastatin were effective at inhibiting T-cell proliferation against polyclonal and antigen-specific stimuli by inducing cell cycle arrest in the G0/G1 phase. Additionally, statins’ ability to promote regulatory T-cell action by dually inhibiting IL-6 and promoting antigen-presenting cells to be more “tolerogenic” highlights the drug’s therapeutic potential in mitigating the immunologic landscape associated with OA.

Modulation of immune cell phenotypes within the tumour microenvironment (TME) is a pivotal determinant of cancer progression. In this context, diverse leukocyte populations undergo reprogramming to adopt tumour-supportive roles. These include myeloid lineage cells such as tumour-associated macrophages (TAMs), tumour-associated neutrophils (TANs), and myeloid-derived suppressor cells (MDSCs); lymphoid subsets such as CD4^+^ T helper cells and regulatory T cells (Tregs); and antigen-presenting populations such as dendritic cells (DCs). Tumour cells exploit this phenotypic plasticity through alterations in cytokine signalling, thereby fostering an immunosuppressive milieu that subverts anti-tumour immunity and promotes malignant growth [258,259]. In CRC, MDSCs are among the most prevalent immunosuppressive populations, known to inhibit T- and NK-cell activity while promoting the expansion of Tregs and TAMs and thus contributing to immune evasion and metastasis [260]. Additionally, CRC-derived CCL5 has been shown to recruit and polarize suppressive immune cells, including Tregs, TAMs, and mast cells, while activating pathways such as CCR5-p65/STAT3 to suppress cytotoxic T-cell activity further aiding in immune escape [261]. In the context of breast cancer, TAMs are among the most prevalent immune cells within the tumour microenvironment and are linked to more aggressive disease characteristics, such as poor prognosis and resistance to hormone therapies [262]. Similarly, in lung cancer, immune resistance often arises from multiple factors such as reduced antigen presentation, impaired T-cell responses, and activation of alternative immune checkpoints all of which contribute to reduced tumours less responsiveness to immunotherapy [263]. As a result, targeting the immune microenvironment has become a central strategy in overcoming tumour progression and therapeutic resistance.

Statins, in particular, have demonstrated immunomodulatory and anti-tumour effects across various cancer types. In colorectal cancer, they have been associated with reduced angiogenesis in early stages and increased Treg infiltration in advanced stages, indicating stage-specific mechanisms that may limit tumour aggressiveness [264]. Additionally, statins may impair immunosuppressive signalling by lowering cholesterol levels in tumour cells, thereby restoring T cell function. Additionally, statins may impair immunosuppressive signalling by lowering cholesterol levels in tumour cells, thereby restoring T cell function [265]. In the early stages of lung cancer, statin use has been associated with reduced infiltration of pro-tumorigenic TAMs in premalignant adenocarcinoma lesions, indicating a potential chemopreventive effect [266]. In the context of non-small cell lung carcinoma (NSCLC), statin administration has been reported to attenuate PD-L1 surface expression and trigger ferroptotic cell death pathways, thereby potentially constraining tumoural aggressiveness and metastatic competence [267]. Within experimental breast carcinoma paradigms, lovastatin has been shown to recalibrate tumour-associated macrophage (TAM) polarization toward a classically activated, M1-skewed phenotype, a shift mechanistically linked to suppression of IL-10 and concomitant augmentation of IFN-γ expression within CD45^+^ leukocyte populations [268]. Moreover, combinatorial regimens incorporating lovastatin with the microtubule-stabilizing chemotherapeutic paclitaxel have demonstrated synergistic efficacy, characterized by enhanced intratumoural infiltration of cytotoxic CD8^+^ T lymphocytes, downmodulation of PD-L1 expression, and superior tumour growth control in both in vitro and in vivo experimental frameworks [269]. By modulating macrophage polarization, enhancing T cell activity, and suppressing immune checkpoint expression, statins demonstrate potential as adjuncts to immunotherapy. Their consistent effects across colorectal, lung, and breast cancers suggest a common mechanism of immune modulation that merits further clinical investigation.

### 4.3. Endothelial Protection and Inflammation Reduction

Statins can also significantly improve endothelial dysfunction and oxidative stress. Endothelium health is an important factor to consider in inflammatory conditions such as diabetes, OA, and cancer; besides the release of inflammatory factors and immune cell adhesion, in conditions that are hyper-dependent on endothelium for their pathogenesis, statins’ effect must be investigated to investigate if it can attenuate their progression.

The state of chronic hyperglycemia in diabetes is the underlying mechanism of the disease’s pathogenesis. While in non-diseased individuals, glucose can be taken by metabolic cells via insulin stimulation, the insulin dysfunction in diabetic patients does not allow for such measures, leading to a pathologic state of hyperglycemia. Hyperglycaemia constitutes a critical precipitant of diabetic cardiomyopathy, primarily through endothelial perturbations mediated by non-enzymatic protein glycation and the subsequent accumulation of advanced glycation end-products (AGEs) [270]. The initiation and progression of such endothelial dysfunction are governed by a multifactorial interplay involving impaired nitric oxide (NO) bioavailability, elevated oxidized low-density lipoprotein (ox-LDL) burden, and heightened inflammatory mediator activity—each of which is adversely modulated by hyperglycaemia-induced downregulatory effects [271,272].

NO is a key modulator of endothelial function (anti-inflammatory and vasodilatory effects) [273]. Hyperglycemia can trigger depletion of NO by impairing eNOS activity, via decreased expression, impairing tetrahydrobiopterin (BH4) formation leading to eNOS uncoupling [272,274]. Furthermore, hyperglycemia can inhibit NO’s activity directly via an increased production of superoxides which can deactivate NO by forming peroxynitrite (ONOO-) [275]. On the other hand, the role of hyperglycemia and ox-LDL is another major contributor to this on-going inflammatory endothelial damage in diabetes. The hyperglycemic state and increased oxidative stress leads to ox-LDL, which in turn leads to endothelial dysfunction, positive reinforcement of oxidative stress, reduced NO and activation of pro-inflammatory pathways [275]. To add to this, once inflammatory cells and cytokines are stimulated and recruited to the endothelium, they also add to the eNOS impairment and oxidative stress, creating another debilitating and progressive cycle of endothelial damage in diabetic patients. 

Multiple experimental studies have delineated a favourable modulatory influence of statins on the nitric oxide (NO) axis and endothelial nitric oxide synthase (eNOS) functionality. In male *db*/*db* mice, Luo et al. demonstrated that oral administration of simvastatin at 40 mg/kg/day elicited a pronounced induction of endothelial Krüppel-like factor 2 (KLF2) expression within the aortic endothelium, a transcriptional programme that translated into enhanced eNOS catalytic activity and, consequently, greater endothelial resilience via NO-mediated cytoprotection [276]. Expanding this line of enquiry, Ota et al. examined the actions of atorvastatin, pravastatin, and pitavastatin in endothelial cultures subjected to oxidative insult—a milieu conducive to premature endothelial cellular senescence and thereby to vascular dysfunction and atherogenic remodelling [277]. The authors reported that all three agents facilitated phosphorylation of Akt at Ser473, which, in turn, augmented eNOS-dependent NO generation in senescent human umbilical vein endothelial cells (HUVECs). In *Sirt1*^+/–^ murine models of streptozotocin-induced diabetes, pitavastatin treatment was likewise associated with a discernible upregulation of eNOS expression. Complementary findings by Li et al. revealed that rosuvastatin conferred neurovascular benefits in diabetic neuropathy, acting through neuronal nitric oxide synthase (nNOS)-driven NO signalling to restore endoneurial vascular perfusion, preserve neural NO bioactivity, and improve overall nerve functional integrity [278]. Mason et al. further provided evidence that atorvastatin ameliorated eNOS uncoupling phenomena, thereby reinstating endothelial homeostasis through the restoration of NO signalling capacity [279]. Similarly, Tian et al. observed that rosuvastatin administration in *db*/*db* mice enhanced NO signalling potential by attenuating reactive oxygen species (ROS) production downstream of angiotensin II type 1 receptor (AT_1_R)-linked NAD(P)H oxidase activation [280].

The NF-κB pathway crosslinks with adhesion molecule expression, such as ICAM-1, LFA-1, and MAC-1 on endothelial cells [281]. These adhesion molecules serve as pivotal mediators in the tethering, firm arrest, and subsequent transmigration of inflammatory leukocytes across the vascular endothelium, thereby sustaining the self-amplifying inflammatory milieu characteristic of diabetic vasculopathy and contributing to progressive endothelial structural and functional compromise [282,283]. There is also strong evidence to show the inhibitory effects statins have on not only the NF-κB transcription, but also on these vital adhesion molecules. Rezaie-Majd et al. investigated the effects of simvastatin intervention in a group of hypercholesterolemic patients with high diabetes risk and vascular inflammation. Observed by the researchers in both in vivo and vitro settings, simvastatin significantly reduced ICAM-1 and LFA-1 expression on monocytes and endothelial cells, at a genomic (mRNA) and proteomic level [284]. However, Rezaie-Majid et al. were not the only ones to note these lowering effects; Nomura et al. saw a significant decrease in sE-selectin and sL-selectin levels in hyperlipidemic diabetic patients after 6 months of pitavastatin treatment [285].

In the context of T1D, evidence from a randomized, double-blind clinical trial demonstrated that atorvastatin administration augmented both endothelium-dependent vasodilation, as assessed by flow-mediated dilation, and endothelium-independent vasodilation, as determined by glyceryl trinitrate–mediated dilation, within the brachial arteries. These vascular benefits were mechanistically linked to enhanced nitric oxide (NO) bioactivity concomitant with attenuation of oxidative stress [286]. Complementary in vitro investigations revealed that cerivastatin and fluvastatin upregulated mRNA expression of GTP cyclohydrolase (GTPCH), the rate-limiting enzyme in tetrahydrobiopterin (BH4) biosynthesis, and increased endothelial nitric oxide synthase (eNOS) abundance in human umbilical vein endothelial cells (HUVECs) [287]. Similarly, Wenzel et al. also demonstrated how in male Wistar rats, atorvastatin was able to upregulate (GTPCH-I), a key enzyme for de Novo BH4 synthesis. This increased BH4 would allow for augmented eNOS activity and endothelial protection [288]. In addition, researchers have also investigated the efficiency of statins over other medications. For instance Landmesser et al. found that flow-mediated dilation, superoxide dismutase activity and active endothelial progenitor cells number was markedly improved after simvastatin, as compared to ezetimibe treatment, which showed no improvement [289]. Lastly, studies showed that this improvement in endothelial function was not just localized to improve NO functionality but also connected to ox-LDL levels. Sallam et al. conducted a randomized control trial showing how the combination of amlodipine with atorvastatin improved the lipid profile [290]. Moreover, Tsimikas et al. conducted a study with 2341 patients affected by acute coronary syndrome, assessing their ox-LDL content in blood samples at baseline and after treatment with 80 mg per day atorvastatin or placebo. The researchers found a reduction in all ox-LDL carried by apoB-100 particles, suggesting atorvastatin’s potent anti-inflammatory activity. Furthermore, the researchers also noted a small increase in lipoprotein (a), suggesting atorvastatin may upregulate levels of lipoprotein (a) in order to mobilize and clear ox-LDL [291]. Overall, these studies suggest to the scientific community the clinical relevance of statins in diabetic endothelial management.

The synovial membrane is a highly vascularized tissue, that plays a key role in the development of OA [292]. Activation of endothelial cells provokes an enhanced transcriptional and translational programme for adhesion molecules, thereby facilitating the tethering, firm adhesion, and subsequent transendothelial migration of circulating leukocytes into the synovial compartment [293], a process that sustains and amplifies the local inflammatory milieu. Statins’ modulation of NO production and interference with surface adhesion molecules expression (such as E-selectin and ICAM-1) underlie their vasodilatory and anti-inflammatory properties [22,294].

The inflamed synovial microenvironment is characterized by significant hypoxia due to a mismatch between the metabolic demands of the hyperplastic synovial tissue and its blood supply [295,296]. This local hypoxia can itself be a damaging stimulus, triggering activation of the complement system on the surface of endothelial cells, leading to inflammation and cell injury [297]. Statins have been shown to specifically counteract this hypoxia-induced damage. Under normal oxygen conditions, statins have little effect on the expression of the vital complement-inhibitory protein CD59 on endothelial cells. However, under hypoxic conditions that mimic the rheumatoid joint, atorvastatin induces a potent, dose-dependent upregulation of CD59 expression [298]. This upregulation, which is dependent on HMG-CoA reductase inhibition and NO production, provides the endothelium with enhanced protection against attack by the complement membrane attack complex (MAC). This finding suggests that the vasculoprotective effects of statins are not generic but are amplified within the specific pathological milieu of an inflamed joint. The effect of statins on angiogenesis itself is more complex and appears to be biphasic; low concentrations can be pro-angiogenic, whereas high concentrations are angiostatic [299]. This dose-dependent duality is a critical consideration, as the high doses potentially required for anti-inflammatory effects might simultaneously inhibit beneficial repair processes, adding another layer of complexity to their therapeutic use in OA.

In the context of cancer, sustained inflammation within the TME can cause endothelial cell dysfunction, thereby promoting tumour progression and metastasis. These dysfunctional endothelial cells influence cancer progression through several mechanisms, including disrupted cell adhesion, increased vascular permeability, and activation of key pro-inflammatory pathways such as NF-κB and STAT3 signalling [300]. Cancer prevention and treatment can also occur through statins’ other pleiotropic effects of mediating angiogenesis, apoptosis and cell proliferation [300]. These effects have been demonstrated to occur in a dose-dependent manner with, lower doses activating Akt and downstream NO production and higher doses inhibiting production of non-sterol derivatives of mevalonate. In CRC, tumour-associated endothelial cells (TECs) have been shown to support epithelial proliferation and contribute to immune remodelling [301]. Similarly, in breast cancer, endothelial cells can enhance tumour cell survival even under nutrient-deprived conditions, ultimately increasing metastatic potential [302]. In the context of pulmonary malignancies, particularly non-small cell lung cancer (NSCLC), circulating concentrations of Cripto-1 (CR-1) and vascular endothelial growth factor (VEGF) have been shown to be markedly elevated relative to those observed in healthy control cohorts. Importantly, CR-1 titres exhibited a pronounced increase in patient subsets presenting with distant metastatic dissemination [303]. Given this critical role of endothelial dysfunction in cancer, targeting the vascular component has become a key therapeutic strategy. Statins have shown strong anti-angiogenic properties at micromolar concentrations. They inhibit endothelial cell growth and migration while inducing apoptosis in TECs [304]. In CRC experimental models, co-administration of statins with the anti-angiogenic agent bevacizumab elicited a marked attenuation of endothelial cell viability, invasive capacity, and capillary-like tube morphogenesis, collectively culminating in suppression of tumour expansion and metastatic dissemination [305]. In breast cancer, statin therapy has also been associated with vascular protection, particularly in patients undergoing radiotherapy, where statins like rosuvastatin and pravastatin were linked to a lower incidence of MACE, reflecting improved endothelial function [306]. Likewise, in lung cancer, atorvastatin has been reported to attenuate VEGF expression by limiting reactive oxygen species (ROS) generation through inhibition of Rac1-driven NADPH oxidase, while concurrently enhancing antioxidant defences such as glutathione peroxidase (GPx). This combined effect suppresses pro-inflammatory signalling cascades and preserves endothelial structural and functional integrity [307]. Collectively, these findings underscore the multifaceted mechanistic roles of statins in oncological contexts, highlighting their capacity to modulate tumour-promoting pathways, remodel the tumour microenvironment, and attenuate pro-angiogenic and pro-inflammatory signalling, thereby contributing to improved therapeutic outcomes. This positions statins as promising adjunct therapies that can improve vascular health and may also boost the effectiveness of current cancer therapies across various tumour types.

### 4.4. Inhibition of Inflammasome Activation

Inflammasome-driven signalling constitutes a pivotal axis in the pathobiology of chronic inflammatory states, orchestrating maladaptive immune activation across metabolic, neoplastic, and degenerative joint disorders. Among these cytosolic multiprotein platforms, NLRP3 inflammasome represents a principal effector node, whose activation is precipitated by a diverse repertoire of metabolic derangements, redox disequilibria, and sterile danger-associated molecular patterns, thereby amplifying tissue-destructive inflammatory cascades and accelerating disease trajectory. The involvement of the NLRP3 inflammasome in the pathophysiology of diabetes remains a subject of considerable scientific contention, with experimental and clinical evidence yielding divergent interpretations. Certain studies delineate a context-dependent regulatory role, wherein NLRP3 activation mediates controlled inflammatory resolution through downstream IL-1 family signalling attenuation, thereby exerting anti-inflammatory influences. Conversely, other investigations implicate sustained or dysregulated NLRP3 activation in the perturbation of insulin receptor signalling cascades via chronic IL-1β overproduction, oxidative stress amplification, and crosstalk with stress-activated kinases, ultimately impairing insulin sensitivity and glycaemic control. This apparent dichotomy underscores the necessity for refined mechanistic dissection of inflammasome kinetics, spatial activation patterns, and cell-type-specific contributions within metabolic tissues.

NLRP3 is part of the innate immunity; as such, it is stimulated by DAMPs and activates pro-apoptotic caspases for maturation and release of other pro-inflammatory cytokines like IL-1β and IL-18 [308]. In diabetes, the body recognizes the abnormal elevated free fatty acids, the sustained hyperglycemic levels and ROS as DAMPs, compelling the trigger of NLRP3 inflammasome activation [309]. Thus, another inflammatory pathway enters the scene to facilitate the beta-cell dysfunction and insulin resistance discussed before.

In osteoarthritis (OA), a comparable pattern of inflammasome misregulation is evident; persistent hyperactivation of the NLRP3 inflammasome constitutes a central pathogenic axis driving articular cartilage matrix attrition and synovitis, thereby promoting progressive disease evolution [310]. NLRP3 inflammasome functions as a sensor to endogenous metabolic stressors such as cholesterol crystals by recruiting and activating caspase-1 [131,310].

In oncological contexts, an accumulating body of evidence indicates that multiple malignancies are characterized by heightened NLRP3 inflammasome activation. This dysregulated inflammasome signalling contributes to tumour growth and has been linked to poorer clinical outcomes in several malignancies. NLRP3 activation in macrophages promotes invasion and migration of tumour cells by driving epithelial–mesenchymal transition (EMT) [311]. Similarly, in lung cancer, stimulation of the inflammasome with LPS and ATP enhances proliferation and migration of cells [312]. Clinically, high NLRP3 expression in lung adenocarcinoma is associated with increased infiltration of immunosuppressive M2 macrophages and poorer survival outcomes [313]. Additionally, cancer-associated fibroblasts respond to DAMPs by activating the inflammasome and releasing IL-1β, amplifying pro-inflammatory signalling in the tumour stroma [314].

In the context of diabetes, Lv et al. demonstrated that simvastatin can inhibit activation of NLRP3 inflammasome in vascular endothelial cells thereby protecting against hyperglycemia-induced endothelial dysfunction and improving vascular permeability [315]. Similarly, Luo et al. successfully showed how rosuvastatin can elevate inflammatory-driven diabetic cardiomyopathy by downregulating NLRP3 inflammasome activity along with MAPK pathway [316]. Furthermore, evidence indicates that statins can attenuate lysosomal injury–driven activation of the NLRP3 inflammasome, thereby mitigating obesity-associated increases in endothelial permeability [317]. Despite the study being scoped on obesity, these results can be utilized for diabetic patients management as well, since obesity is a major factor in the pathogenesis of diabetes [318]. Furthermore, statins when taken in combination also demonstrate the same inhibitory activity, as investigated by Wang et al. Two statins—simvastatin and mevastatin—were administered in endothelial cells to see if statins can improve endothelial outcomes. Collectively, the statins show inhibitory activity of inflammasome in the endothelial cell lines. Although not particularly investigated in the context of diabetes, the same pregnane X receptor (PXR)-dependent mechanism is also present in diabetes, hence the translational relevance of the study can be seen [319].

Statins administered in combination therapy with other non-statin drugs can increase the potency of targeted action. In a cohort of insulin-resistant Wistar rats, treatment was administered either with dapagliflozin alone or in combination with atorvastatin [320]. In the monotherapy group, dapagliflozin partially reversed metabolic disruption and reduced kidney injury in these rats; however, the effects were much less compared to the combination group, which demonstrated substantial improvement in inflammasome activation and autophagy dysfunction.

Conversely, some studies have reported how statins exacerbate diabetic pathogenesis by increasing risk of new-onset diabetes. Recently, a study by Henriksbo et al. found atorvastatin activated p38, hence primed p38 to act on NLRP3 inflammasome [151]. Similarly, a study by Henriksbo et al. demonstrated that fluvastatin dose-dependently enhanced IL-1β secretion from macrophages, indicative of NLRP3 inflammasome activation [321]. Both sides carry robust evidence, hence warranting further in-depth investigation of statins in NLRP3 inflammasome activity.

In OA as well, statins appear to modulate inflammasome activity. In clinical studies, statin therapy was shown to downregulate the gene expression of NLRP3 in PBMCs from patients with cardiovascular disease [322,323]. As previously mentioned, this is further substantiated by an in vivo study on vascular endothelial cells, where simvastatin and mevastatin significantly suppressed NLRP3 inflammasome activation [139]. In rat model investigations, atorvastatin has been found to attenuate NLRP3 inflammasome activation in intracerebral hemorrhage by interfering with TLR4 and MyD88 signalling pathways, and likewise in TNF-α–stimulated nucleus pulposus cells, which display phenotypic similarities to chondrocytes [324].

It is important to note statins’ opposing effect on the NLRP3 inflammasome- initiating activation through the upregulation of IL-1β synthesis and enhancement of caspase-1 enzymatic function [151]. This contradictory action can depend on the lipophilicity of the statin used, the signalling pathways involved, the current metabolic state, and the initial inciting event [131,151,325]. For OA, it implies that a statin could potentially be beneficial by suppressing inflammasome activity in chondrocytes and synoviocytes while having neutral or even detrimental effects elsewhere.

While direct studies in tumour models remain limited, mechanistic findings from related systems suggest that statins may exert anti-inflammatory effects in cancers characterized by high oxidative stress. Evidence indicates that rosuvastatin attenuates ox-LDL–driven upregulation of thioredoxin-interacting protein (TXNIP), a pivotal upstream regulator of NLRP3 inflammasome activation, thereby diminishing IL-1β–dependent inflammatory signalling [326,327]. Although these findings derive from non-cancer models, the specific role of statins in inhibiting inflammasome activation within cancer models remains underexplored. Thus, it is plausible that statins may show comparable anti-inflammasome effects in cancer contexts, particularly in malignancies characterized by high oxidative stress.

In summary, statins modulate NLRP3 inflammasome activity through mechanisms such as NF-κB inhibition, TXNIP suppression, and PXR activation. While anti-inflammatory effects have been observed in models of diabetes, cancer, and osteoarthritis, findings in diabetes and OA remain mixed, suggesting statin responses may be context-dependent, influenced by factors like statin lipophilicity, tissue environment, and disease stage. 

### 4.5. Modulation of Protease-Activated Receptor-2 (PAR-2) Signalling

Protease-activated receptors (PARs 1–4) constitute a subclass of G-protein–coupled receptors engaged in diverse physiological and pathophysiological processes [328]. These are key mediators of cellular responses in inflammation and disease progression. Although direct studies on statins’ effects on PAR signalling are scarce, emerging evidence, particularly on PAR-2, suggests statins may attenuate this pathway, contributing to their therapeutic effects across diabetes, cancer, and osteoarthritis.

In diabetes specifically, PAR-2 has been shown to stimulate inflammatory pathways and attenuate cellular metabolism, increasing insulin resistance and promoting obesity [329]. PAR-2, in particular, has been implicated in driving inflammation through activation of NF-κB and induction of TNF-α [330]. In work conducted by Hayashi et al., the interplay between PAR-2 and factor Xa was examined within the context of inflammation-driven diabetic nephropathy. In this condition, factor Xa levels are elevated, which in turn enhances PAR-2 signalling, with both elements acting synergistically to sustain inflammation through NF-κB and MAPK pathways [331]. Furthermore, PAR-2 has been observed to promote fibro-proliferate disorders, initiate podocyte injury, tubular epithelial cell inflammation and kidney damage via IgA-induced nephropathy [332].

In OA, the inflamed joint, marked by its catabolic environment, is rich in enzymes released from synovial cells and infiltrating leukocytes that can degrade the cartilage matrix. PAR, specifically PAR-2, is a key sensor for these cellular responses by inciting pro-inflammation and catabolism that drives joint destruction [333,334,335,336,337]. Studies have demonstrated a marked upregulation of PAR-2 in cartilage and chondrocytes derived from OA patients, as compared to those from normal, healthy tissue [338]. In OA, elevated PAR-2 expression increases the sensitivity of chondrocytes to proteolytic stimuli present in the surrounding matrix, leading to the upregulation of matrix metalloproteinases such as MMP-1 and MMP-13, as well as induction of the inflammatory enzyme cyclooxygenase-2 (COX-2) [338]. This downstream effect is driven by the activation of intracellular signalling pathways involving Erk1/2 and p38 MAPK. Additionally, targeting PAR-2 expression in OA has shown potential therapeutic benefit in a study investigating oleocanthal’s activity in in vitro human chondrocyte models [339]. Treatment with Oleocanthal led to a significant, dose-dependent reduction in PAR-2 expression, accompanied by decreased levels of pro-inflammatory cytokines such as TNF-α and IL-1β.

A comparable pattern of dysregulation is evident in oncology, with PAR-2 overexpression documented across diverse tumour types. In prostate and pancreatic cancers, elevated PAR-2 levels have been linked to enhanced proliferative capacity, while in colorectal malignancies, overexpression correlates with more aggressive invasion patterns. Similar associations are seen in hepatic and cutaneous cancers, where heightened PAR-2 expression is tied to accelerated tumour progression and poorer patient prognosis [340,341]. In CRC particularly, PAR-2 activation reduces doxorubicin-induced cell death by promoting anti-apoptotic signalling [342]. Research findings indicate that PAR-2 drives breast-cancer cell migration and invasion [343] and in lung adenocarcinoma its overexpression is associated with lymphatic spread and poor postoperative survival [344].

Statins’ effects on PAR-2 modulation have been investigated in several cell lines, including HUVECs and CRC cell lines [333,345]. The former study showed how statins prevent the induction of tissue factor by specific PAR-2 activating peptides, proving statins can exert upstream regulatory control [333]. Although direct studies examining the effects of statins on PAR-2 signalling in specific diseases remain limited, Patnaik et al. demonstrated that both atorvastatin and rosuvastatin selectively suppress PAR-2 expression at the transcript and protein levels in a dose-dependent fashion [345]. These downstream events resulted in diminished release of the pro-inflammatory cytokine TNF-α and a mitigation of aberrant calcium signalling. Although conducted in a cancer model, the core mechanism, i.e., attenuation of PAR-2 activity, may be relevant across multiple inflammatory conditions where PAR-2 is implicated to play a pathogenic role.

In our recent investigation, we examined the effects of the lipophilic statin atorvastatin and the hydrophilic statin rosuvastatin on PAR-2 expression in the human CRC cell lines HT-29 and Caco-2. Both agents elicited a marked downregulation of PAR-2 at both the protein (translational) and mRNA (transcriptomic) levels, accompanied by a concomitant suppression of the pro-inflammatory cytokine TNF-α [326]. Furthermore, statin treatment favourably modulated intracellular calcium dynamics, an effect with potential implications for PAR-2-dependent signalling cascades, inflammatory amplification, and tumour cell survival [326]. However, these findings warrant further extension through mechanistic dissection of the downstream signalling networks, assessment of PAR-2 knockdown or knockout models, and evaluation of the potential synergistic effects of statins with conventional chemotherapeutics or targeted agents. Such studies will be essential to fully delineate the therapeutic potential of statin-mediated PAR-2 modulation in CRC and its impact on tumour progression, angiogenesis, and the inflammatory tumour microenvironment. Importantly, the implications of statin-mediated PAR-2 modulation may extend beyond the cancer cell itself to include alterations in the surrounding stromal and adipose compartments, particularly peritumoral adipose tissue (PTAT), which is increasingly recognized as a metabolically active and immunologically dynamic contributor to tumour progression.

PTAT is increasingly recognized as an active participant in tumour progression, functioning not only as a metabolic reservoir but also as a paracrine signalling hub. Recent evidence indicates that PTAT frequently undergoes browning, a phenotypic conversion of white adipocytes into a more thermogenically active, brown or beige-like state. This process is characterized by mitochondrial enrichment, enhanced oxidative metabolism, and upregulation of uncoupling proteins, notably UCP1 [346]. Such metabolic reprogramming fundamentally alters the inflammatory and angiogenic profile of the TME, with consequences for immune cell recruitment, nutrient availability, and cancer cell survival. The phenomenon has been documented in breast cancer, renal cell carcinoma, and likely other malignancies, highlighting its relevance across multiple tumour types [347].

In breast cancer models, tumour-derived adrenomedullin (ADM), a hypoxia-inducible peptide, drives UCP1 expression in adjacent adipocytes through paracrine signalling [348]. This results in delipidation of cancer-associated adipocytes, with smaller lipid droplets, increased mitochondrial density, and heightened thermogenic capacity. These changes facilitate fatty acid mobilization and modulate cytokine networks, ultimately supporting tumour invasiveness. In renal cell carcinoma, perirenal adipose tissue adjacent to the tumour exhibits markedly higher UCP1 expression compared to normal perirenal fat [349]. This browning correlates positively with Fuhrman grade and tumour stage, while histological examination reveals smaller adipocyte size, greater cell density, and increased expression of thermogenic markers. Such findings suggest that browning in PTAT is not merely a by-product of systemic cachexia or metabolic disturbance, but rather a locally orchestrated adaptation driven by tumour–stroma interactions.

PARs, particularly PAR-2, have emerged as important mediators of adipose tissue remodelling within the peritumoral niche [349]. PAR-2 activation in the TME has been linked to enhanced cancer cell survival, reduced chemosensitivity, and the upregulation of anti-apoptotic proteins. In breast cancer models, genetic deficiency of PAR-2 delays tumour progression and reduces angiogenesis, implicating it in the angiogenic switch associated with browning and vascular remodelling [350]. Mechanistically, PAR-2 may influence adipocyte phenotype through ERK/MAPK and NF-κB signalling, which intersect with transcriptional programmes controlling UCP1 expression and mitochondrial biogenesis.

Statins, by inhibiting HMG-CoA reductase, modulate isoprenoid-dependent signalling, mitochondrial function, and inflammatory pathways. These pleiotropic effects create a plausible framework for statins to influence adipocyte browning in the peritumoral compartment. A key mechanism involves activation of AMPK, a master regulator of metabolic reprogramming in adipose tissue. Statin-induced AMPK activation can promote mitochondrial biogenesis via PGC-1α and PPARγ coactivation, enhance fatty acid oxidation through acetyl-CoA carboxylase phosphorylation and carnitine palmitoyltransferase 1 activation, and induce UCP1 expression, driving thermogenic programming in white adipocytes.

However, this relationship is not straightforward. Recent data suggest that statins may paradoxically suppress browning in certain contexts [351]. In vitro experiments have shown that statin treatment can reduce UCP1 and thermogenic gene expression in human white adipocytes, while short-term statin administration in animal models has diminished brown adipose tissue activity [352]. This suppressive effect is thought to occur through depletion of geranylgeranyl pyrophosphate, an essential intermediate for the prenylation of small GTP-binding proteins such as Rho and Rac1, which are necessary for adipocyte differentiation and mitochondrial recruitment. Thus, the influence of statins on browning is likely dose- and context-dependent, with potential differences between short- and long-term exposure, as well as between tissue types.

Regardless of their effects on browning per se, statins consistently demonstrate significant anti-inflammatory activity within adipose tissue depots. They reduce the proportion of pro-inflammatory macrophages, promote M2-like anti-inflammatory macrophage polarization, and attenuate inflammatory cytokine production [353]. In addition, statin therapy has been associated with reductions in epicardial adipose tissue thickness and improvements in local immune–metabolic profiles [354]. Within PTAT, these anti-inflammatory actions could indirectly suppress tumour-promoting inflammation, even in scenarios where browning is diminished.

Although direct clinical evidence linking statin therapy to modulation of PTAT browning in cancer patients is currently lacking, converging observations from basic and translational studies support a testable hypothesis. Statins, through AMPK activation and mitochondrial regulation, their modulation of macrophage phenotypes, and potential interference with PAR-2 signalling, could alter the immune–metabolic crosstalk within the peritumoral adipose compartment. These interactions may influence oxidative metabolism, angiogenesis, and inflammatory signalling, thereby impacting tumour progression.

Future research in this domain should focus on integrating advanced imaging modalities with biopsy-based assessments to quantify PTAT browning in cancer patients undergoing statin therapy. In vitro co-culture models incorporating tumour cells, adipocytes, and macrophages will be essential for dissecting the interplay between statin pharmacodynamics, PAR-2 activity, and UCP1 expression. Multi-omics approaches, including transcriptomics, proteomics, and metabolomics, could illuminate the metabolic rewiring of PTAT under statin influence, and clinical correlation with tumour aggressiveness, treatment response, and survival outcomes will be crucial to establish translational relevance.

If validated, the capacity of statins to modulate PTAT phenotype, either by directly altering thermogenic programming or indirectly via anti-inflammatory effects, could add a novel dimension to the pleiotropic anti-cancer mechanisms of this drug class. Such insights would not only deepen understanding of tumour–stroma metabolic interactions but also open avenues for targeted therapeutic strategies combining metabolic modulation with conventional or immune-based cancer therapies.

### 4.6. Antioxidant and ROS Scavenging Activity

The pathological involvement of oxidative–antioxidant imbalance in inflammation-driven disorders such as OA, diabetes, and cancer has been extensively examined, both to delineate the mechanistic underpinnings of disease pathogenesis and to identify prospective avenues for therapeutic intervention. ROS are part of the normal inflammatory signalling and immune protection; however, when levels reach pathogenic levels, it can lead to mitochondrial dysfunction, increased inflammation, and further metabolic dysregulation [355].

Researchers have documented a pronounced disequilibrium between reactive oxygen species (ROS) generation and the endogenous antioxidant defence network in diabetes, a disruption that underpins the persistent oxidative stress characteristic of the condition [356]. The underlying hyperglycemic state promotes ROS production through multiple pathways that ultimately activate the NADPH oxidase enzyme, a key synthesizer of ROS [356,357]. Rac1, a key subunit of NADPH, can become dysregulated as highlighted earlier and can further increase NADPH enzyme levels. Furthermore, elevated activation of NADPH can amplify ER stress, which exacerbates the insulin resistance experienced in diabetes [358]. This results in a positive loop of further protein folding dysfunction and NADPH activation, which worsens the chronic inflammatory state and overall prognosis. This hyperglycemia-stimulated NADPH oxidase activity and downstream cascade has several complications, one of which is in a diabetic retinopathy model [359]. ROS generated via NADPH oxidase serves as an essential mediator in the signalling cascade that drives hypoxia-induced vascular endothelial growth factor (VEGF) synthesis and the subsequent angiogenic response. Similarly, within OA joints, excess ROS production contributes directly to chondrocyte dysfunction, promoting cellular senescence, apoptosis and enzymatic degradation of the extracellular matrix components [360,361]. This is exacerbated with comorbidities such as hypercholesterolemia, which has been proven to induce profound mitochondrial dysfunction in chondrocytes. Furthermore, multiple studies have shown the crosstalk between NOX activity and ER stress, contributing to the insulin resistance experienced in diabetes [362,363].

Cancer cells typically exhibit a state of heightened oxidative stress, characterized by persistently elevated intracellular levels of reactive oxygen species (ROS), which act as critical mediators in both the initiation and progression of malignant transformation [364]. These reactive intermediates engage in deleterious interactions with essential cellular macromolecules, including nucleic acids, proteins, and membrane lipids—thereby inducing cumulative oxidative damage that underpins genomic instability and functional impairment of key regulatory pathways. In CRC, sustained ROS overproduction has been mechanistically linked to the promotion of carcinogenesis, dynamic remodelling of the tumour microenvironment, and the emergence of therapeutic resistance phenotypes [365,366]. Analogously, in breast cancer, ROS act as potent drivers of both genetic reprogramming and immunomodulatory alterations, fostering an environment conducive to tumour growth, metastatic dissemination, and evasion of therapeutic pressure [367]. Moreover, estrogen-mediated ROS generation constitutes a pivotal pathogenic axis in breast carcinogenesis, wherein it amplifies the transcriptional upregulation of pro-proliferative and pro-inflammatory cytokine networks, further potentiating neoplastic progression [368].

Statins’ antioxidative properties—encompassing direct free radical neutralization, suppression of pro-oxidant enzymes, and curtailment of excessive ROS generation—underscore their potential to attenuate the pathogenesis of diverse inflammatory disorders [360,361,369,370,371,372]. Anjos et al. showed that atorvastatin, when given to T2D patients, inhibited NADPH-oxidase dependent ROS generation [370]. Similarly, Cheng et al. highlighted the role of pitavastatin in reno-protective features in Dahl salt-sensitive rat model. They found that among other anti-inflammatory activity, it attenuated NADPH oxidase activity and also ameliorated Rac1 expression [373]. Piconi et al. cultured HUVECs treated with rosuvastatin at different glucose concentration and found that rosuvastatin inhibition of the overexpression of genetic subunits of NADPH oxidase, such as p47-phox, p67-phox, and p22-pho [374]. Additionally, Bruder-Nascimento et al. elucidate Rac1-sensitive NOX mechanisms whereby atorvastatin protects against ROS-mediated vascular injury in diabetes through inhibition of cytosol-to-membrane translocation of p47(phox), Rac1 and Nox1/2/4 [375].

Similarly, another study demonstrated that atorvastatin, pravastatin, and cerivastatin suppress NADPH oxidase function by inhibiting p21 Rac, a critical subunit of the enzyme complex, in rat aortic segments with intact endothelial lining [371]. Conversely, these suppressive effects are not confined to mitigating the chronic inflammatory milieu of diabetes but also encompass downstream sequelae, with studies demonstrating their influence on complications such as diabetic nephropathy and retinopathy. A study evaluating the impact of rosuvastatin on the glomerular filtration barrier in Zucker obese rats demonstrated that the treatment reduced NADPH oxidase activity and enhanced podocyte membrane integrity [376]. Another study conducted in Zucker obese rats found that pitavastatin decreased ROS and NADPH levels (even at an mRNA level) in the mice [377]. Li et al. reported that lovastatin lowered NADPH oxidase 4 expression in retinal capillary endothelial cells (RCECs) as well as in db/db mice receiving lovastatin treatment [298].

Moreover, several studies have also shown the alleviation of ER stress via various statins, which contribute to ROS production in many inflammatory diseases. For instance, Xu et al. demonstrated how rosuvastatin can alleviate ER stress in HUVECs through attenuation of ER stress biomarkers [372]. Another study demonstrated that atorvastatin attenuated activation of the eIF2α–ATF4–CHOP signalling pathway in obese C57BL/6J mice, thereby contributing to the alleviation of endoplasmic reticulum stress [378]. Furthermore, when 3T3-L1 adipocyte cells were treated with simvastatin, it showed a reduction in ER stress via downregulation of ox-LDL-induced ER stress [379]. Such studies underscore the importance of statins’ actions on mitigating the inflammatory milieu underlying diabetes.

In the context of OA, an in vitro comparative study that measured the ability of different statins to antagonize the oxidation of a test substrate by both hydroxyl and peroxyl radicals found that all tested statins exhibited antioxidant activity [369]. Within this class, simvastatin was identified as the most effective scavenger of hydroxyl radicals, while fluvastatin displayed the highest capacity for scavenging peroxyl radicals, indicating differential properties among the various statins. In an animal model where OA was precipitated by a high-cholesterol diet, treatment with atorvastatin was shown to significantly attenuate the progression of cartilage degradation. This protective effect was explicitly associated with the mitigation of chondrocyte mitochondrial dysfunction and the suppression of ROS overproduction. By dually mitigating the imbalance of ROS production, these studies underscore the benefit of treatment with statins in OA patients, especially those with underlying metabolic risk factors.

Due to their role in cancer progression, ROS are increasingly seen as promising therapeutic targets, with statins showing particular potential through their effects mediated by mevalonate pathway inhibition and antioxidant effects [380]. In mouse models of CRC, simvastatin reduced tumour growth by downregulating ROS levels and triggering caspase-1–dependent pyroptosis [381]. In breast cancer cells, statins were shown to activate the antioxidant transcription factor NRF1 via ROS signalling, leading to increased expression of tumour-suppressive miR-140-5p [382]. Together, these findings indicate the central role of ROS in cancer progression and highlight the potential of statins as adjunct therapies with antioxidant and anti-tumour effects.

### 4.7. Reduction in Acute Phase Proteins

Acute phase proteins (APPs) are systematic markers for inflammation that are widely utilized in clinical practice. For systemic diseases such as OA, diabetes and cancer that often had chronic low-grade inflammation, CRP, fibrinogen and serum amyloid A (SAA) are often elevated [383]. In addition to its established diagnostic and prognostic roles, CRP directly participates in inflammatory processes by promoting pro-inflammatory cytokine production and triggering activation of the complement cascade [384].

These APPs been linked to the development of insulin resistance and the progression of metabolic and vascular complications arising from diabetes [385,386]. IL-6 released from adipose tissue and immune cells stimulates the production of CRP and SAA, both of which act to intensify the inflammatory cascade [387]. While their levels are typically elevated to a much lesser extent than in classic systemic inflammatory diseases like RA, they serve as valuable markers for systemic inflammatory burden and disease activity in OA [388]. Similarly, APP are consistently elevated in various cancers and are associated with poorer outcomes. In CRC, increased plasma levels of CRP and fibrinogen have been associated with significantly elevated risk of disease development [389,390]. In breast cancer, CRP and SAA concentrations are elevated in patients with advanced disease stages [391], and higher levels of these proteins have been associated with lower overall survival rates [392]. Notably, SAA expression in TAMs and tumour cells has been linked to the occurrence of lymphovascular infiltration and the spread of cancer to regional lymph nodes [392].In lung cancer, SAA levels were found to be higher in patient serum and plasma [393].

In the PRINCE trial, researchers observed that pravastatin decreased CRP levels, at both 12 and 24 weeks, largely independent of LDL-C reduction [394]. A recent meta-analysis conducted by Mashaba et al. [395] evaluated the impact of statin therapy on C-reactive protein (CRP) concentrations and carotid intima–media thickness (CIMT) in individuals with type 2 diabetes. The analysis demonstrated that statin administration led to significant reductions in both CIMT and CRP, with a daily dose of 20 mg atorvastatin emerging as the most efficacious regimen. Similarly, in another meta-analysis by Zhang et al., the researchers investigated various statin types and dosages in reducing CRP [229]. As previously noted, the JUPITER trial, show that statin therapy lowers serum hs-CRP levels in individuals with dyslipidemia or coronary heart disease, highlighting simvastatin 40 mg/day as a notably effective option, while atorvastatin 80 mg/day demonstrated superior long-term benefits [396]. Since CRP is part of the initial innate immune system response to inflammatory triggers, reduction in CRP levels could suggest a lower incidence of acute and initial-phase diabetes onset and attenuation of the perpetual inflammatory cycle.

In contrast, evidence regarding the influence of statins on serum amyloid A (SAA) remains inconsistent. Experimental studies employing collagen-induced arthritis in mice reported that statin administration did not produce any notable reduction in systemic SAA levels. However, circulating levels of the oxidatively modified lipoprotein complex (SAA-LDL) were significantly reduced in patients with hypercholesterolemia. Given that ox-LDL has been implicated in activating synovial cells and promoting inflammation in the joint [139], the ability of statins to selectively reduce this modified lipoprotein species could be highly relevant to OA pathogenesis. Therefore, further studies investigating molecular mechanisms of statins on these specific biomarkers in OA joints may yield beneficial therapeutic results.

Statins exert pleiotropic effects in cancer by inhibiting tumour cell proliferation, inducing apoptosis, impairing angiogenesis, and modulating the tumour microenvironment, partly through reduction in APPs [219,397,398]. Evidence suggests that atorvastatin significantly reduces IL-6 and CRP levels in cancer patients [397,399], and other research has confirmed statin-mediated CRP reduction across various diseases and cell types [396]. In breast and colorectal cancer specifically, statins have been shown to downregulate inflammatory signalling pathways and may reduce cancer-related mortality, with evidence suggesting that lipophilic statins may be more effective in this regard [400,401]. For liver cancer, statins may attenuate inflammation-driven carcinogenesis, as chronic hepatic inflammation is a key driver of hepatocellular carcinoma [402]. These findings suggest that statins may help mitigate cancer-associated inflammation by downregulating APP levels.

### 4.8. Statins and Rheumatoid Arthritis

RA is an autoimmune chronic inflammatory condition with a multifactorial pathogenesis. It has been hypothesized that targeting the inflammatory pathways in this condition would alleviate its symptoms and slow down the progression of the disease. Additionally, RA is closely linked with increased cardiovascular risk due to chronic inflammation, which exacerbates atherosclerosis and vascular dysfunction [403]. Discontinuation of statins in RA patients is associated with an increased risk of cardiovascular mortality [404]. Therefore, investigating the use of statins to not only modulate the inflammatory pathways involved in RA pathogenesis, but to also alleviate concomitant cardiovascular manifestations, invites exploration into statins as a promising therapeutic option in RA patients.

Studies have demonstrated that atorvastatin effectively exerts its lipid-lowering ability in RA patients [405]. In terms of their anti-inflammatory effects, a meta-analysis showed that atorvastatin significantly decreased erythrocyte sedimentation rate (ESR), CRP, TNF-α and IL-6 levels, markers of inflammation [405,406]. Additionally, an in vivo study has also shown that specific statins such as simvastatin may confer better efficacy than its more popular counterpart’s atorvastatin and rosuvastatin [407]. Conversely, a double-blind study with lovastatin treatment showed no effect on disease activity or CRP levels [408].

A study by Yokota et al. highlighted earlier demonstrated that simvastatin inhibits IL-6 and IL-8 production, as well as cell proliferation induced by TNF-α in fibroblast-like synoviocytes from RA patients [203]. These findings build on those by Takemoto et al. [409], suggesting that simvastatin mediates this anti-inflammatory effect by suppressing Rho and Ras-like protein activities through depletion of isoprenoid compounds. In an in vitro study using PBMC cultures, atorvastatin decreased the levels of IL-17A, TNF-α, IL-6, and IL-10, with the magnitude of suppression varying according to the administered concentration. Analysis showed that cytokine reduction was significant in samples of patients with severe RA [410]. Additionally, research has indicated that atorvastatin improves regulatory T cell activity by inhibiting PI3K-Akt-mTOR and ERK transduction pathways, addressing defects in regulatory T cells implicated in RA [411].

The Trial of Atorvastatin in Rheumatoid Arthritis (TARA) demonstrated that atorvastatin therapy led to a significant reduction in the Disease Activity Score in 28 joints (DAS28), a validated composite index for RA disease activity, with outcomes ranging from moderate to good clinical responses. These improvements were accompanied by marked decreases in erythrocyte sedimentation rate (ESR) and C-reactive protein (CRP) levels [406]. As a follow-up, Mäki-Petäjä et al. demonstrated that simvastatin and ezetimibe resulted in similar decreases in inflammatory markers, supporting the notion that the anti-inflammatory benefits of statins are connected to their cholesterol-lowering effects [412]. Furthermore, Xing et al. proved that, when compared with the used of disease-modifying antirheumatic drugs (DMARDs), atorvastatin showed a significant reduction in DAS28 [405,413]. In contrast, a much larger cohort study conducted by Lodi et al. disproved the findings from the TARA study, highlighting that the anti-inflammatory benefits that statins confer on RA patients do not match those of DMARDs [414].

Across these investigations, the predominant pathway by which statins confer anti-inflammatory effects in RA patients involves elevation of HDL levels [415]. This elevation facilitates cholesterol efflux, thereby diminishing the lipid raft content within immune cell plasma membranes, while concurrently lowering the expression of Toll-like receptor 4 (TLR4) and the interleukin-3 receptor β-subunit (IL-3Rβ).

Although existing evidence suggests that statins may offer anti-inflammatory benefits in RA, further randomized clinical trials and large-scale cohort studies are needed to validate these results and fully establish the therapeutic potential of statins in RA management.

### 4.9. Statins and COPD

Chronic Obstructive Pulmonary Disease (COPD) constitutes a persistent inflammatory disorder of the lower airways, typified by progressive airflow limitation and associated respiratory morbidity. In this context, considerable investigative attention has been directed toward leveraging the pleiotropic anti-inflammatory properties of statins to attenuate acute exacerbations, enhance clinical outcomes, and suppress both inflammatory signalling cascades and the structural remodelling of pulmonary tissue.

In a rat model of COPD, atorvastatin administration attenuated structural alterations of the pulmonary vasculature and dampened inflammatory responses [416]. Histopathological examination demonstrated a pronounced reduction in the accumulation of inflammatory cells within the perivascular compartment, together with amelioration of endothelial damage in the vascular wall. Mechanistically, these effects are linked to inhibition of acetyl-CoA conversion to cholesterol, leading to decreased guanosine triphosphate–binding protein synthesis and prevention of NF-κB nuclear translocation. This signalling interruption suppresses the release of pro-inflammatory cytokines, including IL-8 and TNF-α, ultimately constraining pulmonary vascular inflammation and limiting pathological tissue remodelling. Additionally, atorvastatin was found to increase HDAC2 expression via NF-κB acetylation and reduce vascular endothelial growth factor production, thus modulating pulmonary vascular inflammation and remodelling. However, such anti-inflammatory benefits are dose-dependent, as high doses may further exacerbate lung damage [417]. A pilot-study conducted by Mroz et al. (ClinicalTrials.gov ID NCT01748279) confirmed atorvastatin’s anti-inflammatory effects [418], with a follow-up clinical trial currently investigating its effect on COPD exacerbations (ClinicalTrials.gov ID NCT04789057).

Statins have also been linked to decreased frequency of COPD exacerbations and related hospitalizations. A population-based nested case–control study reported a 30% reduction in the risk of hospitalization among statin users, with more pronounced effects observed in patients within six months of statins use or at higher doses [419]. The Rotterdam study highlighted the long-term benefits of statin use in reducing mortality in COPD patients, with up to a 39% reduction in all-cause mortality and 64% decrease in pulmonary-associated mortality [420]. The protective effect was particularly significant in COPD patients with elevated systemic inflammation (indicated by high CRP levels), suggesting that statin’s mechanism of modulating inflammatory processes is crucial for mortality reduction. A systematic review and meta-analysis incorporating data from more than 238,000 individuals with COPD, across 15 studies, identified a statistically significant association between statin therapy and reduced mortality risk, specifically, a 38% decrease in all-cause mortality and a 52% decrease in COPD-specific mortality [421].

The concomitant administration of statins with other anti-inflammatory interventions, such as omega-3 fatty acids, which attenuate NLRP3 inflammasome activation, and lycopene, which suppresses IL-6, TNF-α, and IL-1β production—has demonstrated potential in modulating inflammatory pathways in COPD [422]. Interestingly, the study displayed rosuvastatin’s pro-inflammatory properties, as evidenced by increased neutrophilic airway inflammation. The increase in neutrophils was primarily associated with changes in LTB4R and ALOX15 gene expressions, known for their role in neutrophil recruitment.

These results highlight the potential of statins as adjunctive therapy in managing COPD. Future studies should aim to optimize dosing strategies and investigate combination therapies to enhance their therapeutic potential in COPD.

## 5. Limitations

While statins exhibit many beneficial effects, several studied provide an opposing view on their toxic and harmful side effects. The following section discusses the limitations of statins in respect to their effects on organ dysfunction, hormonal axes, epigenetics, and socioeconomic status.

### 5.1. Organ Dysfunction

**Figure 10:**
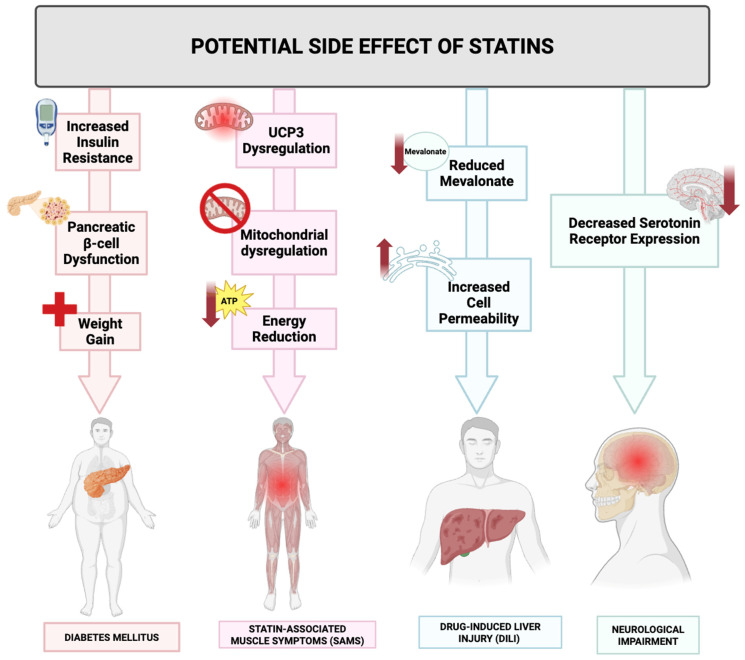
Potential adverse effects associated with statin therapy. This figure depicts the commonly reported adverse effects of statin administration, most commonly statin-associated muscle symptoms (SAMSs) (linked to genetic predisposition and mitochondrial dysfunction), increased risk of T2D, drug-induced liver injury (DILI), and various neurological impairments, all possibly due to altered cholesterol metabolism and subsequent molecular expressions.

Figure 10 demonstrates the toxic side effects of statins on the liver, pancreas, muscle and brain. Statin therapy is frequently accompanied by adverse musculoskeletal sequelae, collectively termed statin-associated myopathies, which represent a subset within the broader clinical spectrum of statin-associated muscle symptoms (SAMS). The exact mechanism of action is still a point of contention; however, it is postulated that the mechanism of action differs due to genetic polymorphism, as genetic dysregulations of specific genes like Uncoupling protein 3 (UCP3) make individuals more susceptible to SAMS [423,424]. Furthermore, statin-mediated downregulation of the intermediates in the de novo cholesterol pathway and Co-enzyme Q10 biosynthesis, leads to mitochondrial dysregulation, reduced energy production and muscular atrophy [425,426]. An observational study by Casula et al. found that 9.6% of the cohort experienced SAMS, with higher rates reported among women and subjects with physical activity [427]. An estimate of 7–29% SAMS was reported by statin-users in various observational studies; this indicates a potential adverse effect of statin therapy [28,427,428].

Furthermore, a series of meta-analyses undertaken at the University of Glasgow consistently delineated an association between statin therapy and an elevated incidence of type 2 diabetes (T2D), with the 2015 meta-analysis specifically reporting an 11% higher relative risk of T2D onset in statin-treated individuals compared to those receiving placebo [429]. While the precise mechanism remains under investigation, statin-induced diabetes has been linked to pancreatic-beta cell dysfunction from increased intracellular cholesterol, weight gain, and augmented insulin resistance.

Statins’ primary mechanism of reducing cardiovascular risk plays an important role in patient with comorbid liver disease, especially fatty liver disease [68]. However, studies exploring this use is limited due their capability of causing drug-induced liver injury (DILI). While statins have beneficial pleiotropic effects on the liver, there have been studies that suggest that reduction in mevalonate may cause an increase in liver enzymes. Furthermore, statins may reduce cell permeability, resulting in increased leakage and elevated detection of liver enzymes, contributing to the DILI [70]. Chen et al. carried out a population based case–control study with 4165 cases and found that statin-induced liver injury was not significant compared to control group; however, when looking at the statin group, a higher dosage of rosuvastatin before DILI occurred showed significant association with liver injury [72].

Statin-administration has been associated with a number of neurological impairments, ranging from aggressive behaviour to decreased serotonin expression. An association between decreased cholesterol and decreased serotonin receptor expression has been postulated [75]. Serotonin is a key neurotransmitter involved in the regulation of mood and behaviour. However, population-based studies have suggested a protective association between statin use and depression [77].

While reviewing studies investigating statins, it is important consider studies reporting the drug’s negative or non-significant results. Table 5 and Table 6 list such studies where statins—used alone, in combination with other statins, or alongside different drug classes– yielded such results. Such limitations add a holistic approach to investigating the drug’s potential therapeutic benefits and provides further research avenues. 

One limitation to the research designs investigating the benefits of statins include the variety of research designs. Observational studies, being purely correlational in nature, contrast with the in vivo animal models. Although they present statistically significant data, the extent to which this data can be applied to human patients is limited in understanding statin-mediated toxicity. Furthermore, often these studies are performed with a limited sample size, which results in reduced statistical power and possible inconclusive data being presented, leading to issues in determining the true effects, extent and clinical application of statins. When extending the results to populations of different gene pools, the results lack predictability. Hence, the cytotoxic effect of statins observed on a specific population and sample might have a different manifestation in a different population, compelling further research into the statin-mediated effects on these conditions. 

Among the studies listed, the randomized clinical trials are ones with the highest internal validity, allowing the researchers to establish a reliable cause-and-effect relationship of the statin variable being studied. However, when investigating statins in clinical trials, another factor to weigh in is the compliance rate. Although randomized clinical trials minimize the researcher bias, inconsistent adherence to trial protocols results in data variability. A meta-analysis encompassing data from 20 studies involving a cumulative cohort of 376,162 patients demonstrated suboptimal adherence to statin therapy, with a substantial proportion discontinuing treatment following initiation [448]. A different retrospective analysis of statin administration was performed, and it was found that there was a decline in statin persistence, with women showing a lower persistence rate than men [449]. Reasons for non-compliance with statins are varied, including, but not limited to, access to medical care, transitions between multiple healthcare providers, and the cost of medications [450].

Beyond issues like medication adherence, another aspect to consider when looking into statins is the difference in concentrations. Statins’ effects on the endothelial cells are dependent on the dosage given, i.e., lower concentrations of statins impose anti-senescence, and antiapoptotic effects, whereas the opposite seems to be true for higher statin dosage [451], which highlights the importance of using consistent dosage of statins in research studies to avoid misinterpretations of results due to varying concentrations.

A point to consider when evaluating statin-mediated effects is the model of the statin being evaluated. simvastatin and atorvastatin are lipophilic statins and rosuvastatin is non-lipophilic. This allows simvastatin and atorvastatin to be more readily absorbed by the body, as their ability to freely permeate the cell membrane allows for greater rates of diffusion and drug utilization. On the other hand, rosuvastatin being hydrophilic allows it to be more readily excreted by the renal system. Taking this into account, when determining the ability of statins to present cytotoxicity or other adverse effects, the efficacy, safety profile and interactions are dynamic in different clinical scenarios, e.g., lipophilic stains are postulated to exhibit higher cardiovascular benefits but result in more SAMS [452].

However, large-scale meta-analyses and randomized controlled trials have shown no clinically significant difference in the incidence of SAMS between lipophilic and hydrophilic statins at equivalent lipid-lowering doses [453,454,455]. In vitro studies suggest greater cytotoxicity of lipophilic statins in muscle cells, especially in genetically susceptible individuals, but this has not translated into a higher clinical incidence of muscle adverse events in the general population. The American Heart Association and the National Lipid Association both emphasize that all statins can cause SAMS, and the choice of statin should be individualized based on patient comorbidities, drug interaction risk, and prior intolerance.

In addition, when considering the therapeutic effect of statins in conditions like CRC, real-world applications of these statins involve co-therapy with other drugs, necessitating a poly-pharmacodynamic evaluation. Investigating any possible synergistic effect occurring would allow statins to be further evaluated as a possible therapeutic avenue. On the other hand, if there is any drug-induced antagonization of statin, that suggests that statins on their own might have a beneficial effect, but in co-therapy that effect may become harmful, allowing healthcare practitioners to further consider how statins may be initiated in future treatment strategies of conditions like cancer, diabetes, or rheumatological conditions. Similarly, researchers studying the pharmacokinetics interaction of these drugs with statins may be presented with drugs “masking” or reducing the bioavailability of statins, potentially hindering their true therapeutic potential. This was observed in the study listed above by Lee et al., which showed that Sarilumab used to treat rheumatoid arthritis reduce bioavailability of statins (Table 6) [439].

Despite the above findings, Rea et al.’s study demonstrated that statin discontinuation was associated with increased risk of hospital admissions [456]. Similarly, among patients with acute myocardial infarction that survived at least 1 year after the event, researchers found an increased risk of all-cause, CVD and non-CVD mortality with low statin adherence, suggesting an association between low-adherence and increased risk in the following years [457].These findings underscore that maintaining high adherence to statin therapy is critical for reducing both cardiovascular events and mortality, and that even moderate lapses in adherence can significantly diminish the protective benefits of statins.

### 5.2. Hormones

While statins are generally safe and effective for lowering low-density lipoprotein cholesterol (LDL-C), understanding how other hormones affect them is pivotal. In liver cells cholesterol can be excreted in the form of bile or can be stored as esters [458]. Cholesterol homeostasis within hepatocytes is subject to regulatory control by thyroid hormones, an effect mediated predominantly via thyroid hormone receptor-β (TRβ) signalling pathways [459]. Under normal conditions TSR-β lowers the level of cholesterol; however, dysregulation in thyroid hormone can have an adverse effect on LDL-C metabolism [460]. For instance, in hypothyroidism, lower levels of TSR-β will lead to higher levels of cholesterol and consequently patients would require higher statin doses to achieve effective lipid lowering. Conversely a meta-analysis showed that 55–60% of patients with untreated hyperthyroidism exhibited liver dysfunction due to high levels of thyroid hormone [461,462]. This dysfunction includes direct liver toxicity and hepatocyte anoxia due to the increased levels of thyroid hormone [463,464]. Since the liver is essential for statin metabolism via CYP450 enzymes, a decrease in the enzyme due to liver dysfunction will significantly reduce statin metabolism potentially increasing the risk of statin-related adverse effects [465,466]. 

Apart from thyroid hormones, research suggests that there are several other hormones that affect statin metabolism, one of which is corticosteroids. Corticosteroids, specifically glucocorticoids, have a significant impact on the liver function by causing liver enlargement, steatosis and glycogenesis. Additionally, high levels of intravenous corticosteroids, particularly methylprednisolone, have been associated with acute liver injury leading to acute liver failure. This impaired liver function caused due to corticosteroids can then affect the body’s ability to metabolize statins, leading to increased levels of statin in the bloodstream with further associated side effects [467].

While certain hormones can affect statin metabolism, there is substantial research revolving around how statins themselves might influence the function of other hormones. Statins inhibit cholesterol biosynthesis, which may have a negative impact on gonadal steroidogenesis [468]. A prospective study conducted involved men with pre-existing T2D that were given high dosage of statins. The study revealed that high dosage of statin caused a decrease in androgen levels in the participants [469]. Subsequent investigations have identified an association between sustained statin administration and a diminution in total circulating testosterone concentrations in males, accompanied by concomitant reductions in sex hormone-binding globulin (SHBG) and dehydroepiandrosterone (DHEA) levels [468,470].

In addition to that, statins act on body’s hormonal balance in other different ways. Research shows that simvastatin and high doses of atorvastatin are significantly associated with impaired glucose metabolism leading to a potentially higher risk of developing T2D in patients, especially those with a family history of the disease [471,472]. In light of the well-documented pathophysiological association between polycystic ovarian syndrome (PCOS), dyslipidemia, and insulin resistance, an independent clinical investigation evaluated the therapeutic impact of concomitant statin and metformin administration in women with PCOS. The findings demonstrated that such combination therapy failed to elicit a meaningful enhancement in insulin sensitivity or a reduction in hyperandrogenic indices in the study cohort [473,474,475]. Furthermore, the impact of statins on patients with existing type 1 diabetes remains a topic of ongoing research; their effectiveness and safety profile for T1D patients require further exploration using in-depth clinical trials. 

### 5.3. Epigenetics

While this review discusses the biological aspect of statin therapy and the potential pleiotropic effects, in order to holistically evaluate statin utilization in patients, the interplay between social and biological mechanisms must also be highlighted. Ochoa-Rosalea et al. conducted 5 cohort studies to look at the epigenetic correlation between stain-usage and risk of T2D, and they found a downregulation of ABCG1 gene expression. ABCG1 plays a role in regulating blood sugar levels, hence its downregulation indicated to the researchers an increased risk of diabetes [476]. Furthermore, some genetic variants may allow statins to become less effective compared to their counterparts. Cano-Corres et al. observed 6 genetic variants effect on statin’s ability to reduce cholesterol levels in hyperlipidemic patients [477]. Among the 6 variants, the researcher observed that patients with variant HMGCR c.1564-106A > G showed the least reduction cholesterol with statin therapy, putting these patients at a disadvantage. Furthermore, Niemi et al. found when observing single nucleotide protein rs4149056 mutations, specifically found in a family of organic anion transporter family which encoded for hepatic organic anion transporter P1B1, that statins may induce downregulation, hence increasing the concentration of hydrophilic statins and plasma, and hence increasing the risk of SAMS [478].

Another gene that has been the focus of research when investigating the pharmacogenetics of statins is the gene the encodes for the CYP450 family of proteins, as it is one of the main classes of proteins responsible for metabolizing statins. A mutation in CYP class of enzyme can lead to inhibited activity of drug, causing excess buildup of statins in plasma and increasing the risk of statin-induced side effects, like rhabdomyolysis. Preissner et al. identified 2000 mutations in the CYP gene, with single nucleotide protein mutations (SNP) having the most impact. The mutations in Caucasians was observed with other ethnicities to reveal 199 SNP mutations affecting this class of enzymes [479], hence suggesting the prevalence of these mutations and their impact in effecting, predicting and allowing personalized treatment to patients under statin care. Mulder et al. found that homozygous carriers of CYP2D6 gene were prone to withdrawing from statin treatment due to rhabdomyolysis [480,481]. CYP3A5 is another enzyme in the class of CYP enzymes that are involved in the hepatic metabolization of statins. Individuals with an CYP3A5 * 1 allele require higher dosages of lipophilic statins to achieve the same therapeutic outcome compared to other individuals with different alleles for the same enzyme [482]. Such studies demonstrate how genetic variations play a significant role in understanding statins effect on patients.

### 5.4. Socioeconomic

There have been a number of studies that investigate the correlation between socioeconomic status (SES), disparity and statin therapy, with many postulating that a lower SES disposes the patient to lower statin adherence, and hence statin benefits. Erickson et al. investigated statin adherence and accessibility to pharmacies in Michigan and found that adherence to statin prescription was lower for patients residing in lower income areas [483]. A study that investigated the correlation of socioeconomic disparity and statin adherence in preventing premature CVD in Hungary found that individuals from a lower tax bracket were less likely to receive prescriptions for preventive statin therapy against CVD [484]. Thomsen et al. found that patients in the retired workers group, when compared to the top managers group, showed lower rates of statin utilization [485]. Socioeconomic background of a patient undertaking statin therapy is significant in determining the success of the therapy. Patients that come from a lower socioeconomic bracket may have difficulty adhering to statin therapy, as seen in these studies, due to multifarious reasons, such as inability to afford medication and other priorities in life interfering in taking the medication. Patients being informed on the significance of undertaking statins or their role in their disease management is another essential factor to ensuring that statin adherence is high and accurate. However, often times, patients from a lower SES background do not have adequate literacy to support their therapy. Furthermore, access to healthcare can also determine the efficacy of statins. Patients from a lower SES background, when compared to their counterparts, have harder times accessing adequate healthcare, which can lead to delays in adjusting statin medications (if needed), regular checkups, and more. Lastly, lifestyle differences in individuals in these varying socioeconomic brackets also affect the efficacy of statins. If an individual is following a relatively healthy lifestyle, compared to a relatively morbid lifestyle, the efficacy of statins will be greater in an individual with a healthy lifestyle. The compliance to a drug being low, administration not meeting the set guidelines, and morbid lifestyle modifications due to socioeconomic disparity is another point for clinical practitioners to consider, as the reduction in statin potential may not be due to the drug itself but due to other systematic factors.

## 6. Conclusions

The anti-inflammatory pleiotropy of statins, historically viewed as a subordinate corollary to their lipid-lowering action, is now assuming the contours of a distinct therapeutic paradigm. By engaging and modulating cardinal molecular circuits—most notably NF-κB, the NLRP3 inflammasome, MAPK cascades, and T-cell lineage specification—statins orchestrate a concerted suppression of pro-inflammatory signalling that traverses the domains of metabolic dysregulation, vascular pathology, and oncogenesis. Such mechanistic breadth, supported by an ever-expanding corpus of preclinical and clinical evidence, compels a reappraisal of statins as immunomodulatory pharmacophores with a therapeutic ambit that transcends their canonical cardiovascular remit.

In an era increasingly defined by polypharmacy, particularly within the management of multimorbid states such as diabetes, cardiovascular disease, and cancer, statins occupy a uniquely advantageous position. Their molecular promiscuity, when judiciously harnessed, permits the attenuation of inflammatory crosstalk that often underlies treatment resistance or drug–drug antagonism. Indeed, their combinatorial deployment alongside nutraceuticals (e.g., oleocanthal, curcumin), anti-cytokine biologics, or cytotoxic chemotherapeutics offers a tantalizing prospect of synergistic immunomodulation coupled with the potential rational de-escalation of toxic drug burdens. The immunomodulatory potential of statins, manifested through their ability to remodel the TME and recalibrate immune effector cell phenotypes, augments their strategic value as co-adjuvants to immune checkpoint blockade or precision-targeted small-molecule therapeutics, especially in neoplastic settings wherein inflammation-driven oncogenic progression constitutes a major impediment to sustained therapeutic efficacy.

The future trajectory of statin research must therefore be anchored in pharmacogenomic stratification to delineate responder phenotypes, unravel inter-individual variability in pleiotropic efficacy, and inform the construction of precision-based combination regimens. Parallel advances in medicinal chemistry could yield second-generation statin derivatives, explicitly optimized for anti-inflammatory potency and minimal diabetogenic risk. Equally, systems biology approaches integrating transcriptomic, proteomic, and metabolomic data will be indispensable in deconvoluting the statin interactome and predicting optimal polypharmacological synergies.

In its broader implications, the evolving narrative of statins underscores a paradigm shift in drug discovery: a recognition that established agents, when interrogated through the lens of network pharmacology, can transcend their original indication to reshape therapeutic landscapes. As chronic inflammation emerges as the cardinal nexus linking cardiometabolic disease, neurodegeneration, and cancer, statins are poised not merely to persist as lipid-centric prophylactics but to assume the mantle of cornerstone modulators within an integrated, multi-targeted therapeutic armamentarium.

## 7. Future Perspectives

The pleiotropic nature of statins, while mechanistically compelling, still demands rigorous clinical translation. Large-scale, genotype-stratified trials that simultaneously capture lipidomic, metabolomic and single-cell transcriptomic outputs are essential to identify the molecular “responders” who derive maximal anti-inflammatory benefit, and to parse out those at highest risk for diabetogenic or myopathic complications. Deep-phenotyping approaches that integrate Mendelian randomisation with multi-omic read-outs should clarify whether on-target (HMGCR-dependent) or off-target pathways, such as TLR4/MyD88, NLRP3 or PAR-2, drive the clinically relevant immunomodulation seen in disparate diseases.

Network pharmacology increasingly reveals that chronic diseases cluster around shared inflammatory hubs [486], repositioning statins within poly-drug regimens that deliberately converge on these hubs could reduce overall pill burden and limit cumulative toxicity. Proof-of-concept studies are already pairing statins with SGLT-2 inhibitors to blunt NLRP3 activity in diabetic kidney disease, and with low-dose colchicine to extinguish residual IL-1β signalling after acute coronary events. The sequence, dose-intensity and relative timing of such combinations will require adaptive platform trials that use early changes in CRP, IL-6 or plaque 18F-FDG (Fluorodeoxyglucose) uptake as gate-keeping biomarkers instead of hard clinical end points.

Second-generation “inflammation-focused” statin analogues are another frontier. Scaffold remodelling aimed at maintaining HMG-CoA reductase affinity while selectively enhancing isoprenoid depletion in macrophages has yielded lead compounds that amplify Treg polarization and M2 macrophage switching without significantly perturbing skeletal muscle prenylation [487]. Coupling these analogues to hepatotropic or leukocyte-targeted nanoparticles may further increase therapeutic indices and allow inhaled, intra-articular, or tumour-localized delivery for COPD, osteoarthritis, or solid-tumour immunotherapy, respectively.

In oncology, the synergy between statins and immune-checkpoint inhibitors merits urgent exploration. Pre-clinical data show that cholesterol efflux driven by ABCA1 upregulation augments CD8^+^ T-cell metabolic fitness and enhances anti-PD-1 efficacy. Small basket trials that integrate high-dose rosuvastatin with PD-(L)1 blockade across inflamed tumours, such as microsatellite-instable colorectal, triple-negative breast, and smoking-related lung cancers, could establish whether statins can convert “immune-cold” lesions to “immune-hot” phenotypes and thereby rescue checkpoint resistance.

Beyond therapeutic development, implementation science must tackle the persistent adherence gap. Digital pill dispensers linked to cloud-based inflammatory dashboards, community pharmacist-led titration clinics, and culturally tailored education modules have all reduced statin discontinuation in pilot studies by up to 30%. Embedding such adherence–support systems in future efficacy trials would yield efficacy–effectiveness concordance data that are currently lacking in real-world cohorts.

Finally, the public health impact of statins in low- and middle-income countries remains under-realized. Cost-efficient fixed-dose combinations that marry generic statins to antihypertensives and nutraceutical antioxidants could generate synergistic anti-inflammatory effects while simplifying pharmacy logistics. Parallel capacity-building efforts should expand pharmacogenomic screening, particularly for SLCO1B15 alleles, in high-prevalence regions to reduce avoidable toxicity and improve trust in generic formulations. Collectively, these avenues herald a shift from cholesterol-centric usage towards a precise and inflammation-modifying paradigm that positions statins as foundational agents across cardiometabolic, rheumatologic, pulmonary, and neoplastic spectra.

## Figures and Tables

**Table 1:** Statin classification with their representative trials.

Statin	Type	Hydrophilic/Lipophilic	Enzyme System for Metabolism	Conditions Used	Representative Trial	Remarks
Atorvastatin	Synthetic	Lipophilic	CYP3A4	Hyperlipidemia, cardiovascular disease	ASCOT-LLA (Anglo-Scandinavian Cardiac Outcomes Trial—Lipid-Lowering Arm)	Highly potent, commonly used in primary and secondary prevention.
Simvastatin	Semi-synthetic	Lipophilic	CYP3A4	Hyperlipidemia, cardiovascular disease	4S (Scandinavian Simvastatin Survival Study)	Derived from fermentation, prodrug that requires activation.
Lovastatin	Natural	Lipophilic	CYP3A4	Hyperlipidemia, cardiovascular disease	AFCAPS/TexCAPS (Air Force/Texas Coronary Atherosclerosis Prevention Study)	First statin discovered, naturally occurring in fungi.
Fluvastatin	Synthetic	Lipophilic	CYP2C9	Hyperlipidemia, secondary prevention of CHD	LIPS (Lescol Intervention Prevention Study)	Less commonly used, shorter half-life.
Pravastatin	Semi-synthetic	Hydrophilic	Sulfation, CYP3A4 (minor)	Hyperlipidemia, cardiovascular disease	CARE (Cholesterol and Recurrent Events)	Lower muscle-related side effects, minimal metabolism by CYP450
Rosuvastatin	Synthetic	Hydrophilic	CYP2C9 (minor), CYP2C19 (minor)	Hyperlipidemia, cardiovascular disease, inflammation	JUPITER (Justification for the Use of Statins in Prevention: an Intervention Trial Evaluating Rosuvastatin)	Highly potent, longer half-life, minimal CYP450 interaction.
Pitavastatin	Synthetic	Lipophilic	CYP2C9, UGT1A3, UGT2B7	Hyperlipidemia, primary prevention	LIVES (Livalo Effectiveness and Safety)	Newer statin, less effect on glucose metabolism.
Cerivastatin	Synthetic	Lipophilic	CYP2C8, CYP3A4	Hyperlipidemia	Withdrawn (due to risk of rhabdomyolysis)	Currently not in use

**Table 2:** Statins’ anti-inflammatory effects investigated in pre-clinical trials.

Statin Type	Population	Intervention	Major Outcomes	Study
Simvastatin	Control mice, Severe asthma mice (House dust mite (HDM)+ Ovalbumin (OVA)+ lipopolysaccharide (LPS)), Conventional asthma mice (Ovalbumin (OVA)).	Control mice treated with saline solution; Severe and conventional asthma mice received either: Cl-amidine 10 mg/kg, DNase I 1000 IU, or Simvastatin 40 mg/kg	Simvastatin-treated mice demonstrated anti-inflammatory activity by reducing the levels of pro-inflammatory cytokines like IL-1β in BALF, increase anti-inflammatory TREG cells and decrease pro-inflammatory Th2 and Th17 in severe asthma mice group.	Chen et al. [51]
Simvastatin	IEC-6 cell line exposed to TNF-α. Non-treated control mice group. Indomethacin only treated mice group. Indomethacin + PPI mice group. Indomethacin + Simvastatin mice group.	IEC-6 cells with inflammation treated with 0.5 μM simvastatin. In the in vivo part of the study, Group 1 was control mice group. Group 2 treated with 12 mg/kg of Indomethacin only. Group 3 with 12 mg/kg Indomethacin + 5 mg/kg of PPI. Group 4 with 12 mg/kg Indomethacin+ simvastatin 15 mg/kg.	In the in vitro experiment, the Simvastatin treated IEC-6 cells showed lower pro-inflammatory markers like IL-6, along with decreased inflammatory proteins like NF-κB. In the in vitro model, the mice treated with Simvastatin showed fewer intestinal bleeding and ROS production.	Kim et al. [52]
Atorvastatin	Rat cartilage explant in osteoarthritic model exposed to IL-1β	Control model. Cartilage model exposed to only IL-1β. Cartilage model exposed to IL-1β + 1, 3 or 10 μM of Atorvastatin. An additional set of each of these cartilage models were also incubated with 1400 W (selective iNOS inhibitor)	Atorvastatin group reduced levels of TNF-α and GAG release, suggesting less cartilage degradation. It also decreased MMP-13 and O_2_^−^ levels. Furthermore, the source of NO in control and Atorvastatin group was eNOS, a beneficial form of NO.	Pathak et al. [53]
Rosuvastatin	Control Mice group. LPS only mice group. LPS+ Rosuvastatin mice group. Rosuvastatin only mice group. RAW264.7 macrophage cell line.	5 mg/kg/day of Rosuvastatin was used. In the In vitro part of the study, RAW264.7 macrophages were cultured with LPS only. Another set with LPS+ 10 and 20 μM of Rosuvastatin	Rosuvastatin prevented sepsis-related death of mice and reduced pro-inflammatory markers like TNF-α. In the RAW264.7 macrophages, rosuvastatin prevented inflammation via downregulating NF-κB.	Tang et al. [54]
Rosuvastatin	Untreated mice group (Dextran sulphate sodium (DSS) only). Treated mice group (Dextran sulfate sodium (DSS)+Rosuvastatin). IEC-6 cell line exposed to TNF-α	Untreated mice group received only DSS. Treated mice group received DSS + 0.3 mg/kg/day of Rosuvastatin for 21 days. The IEC-6 cells were exposed to TNF-α and only some cell cultures were treated with 2 μmol/L of Rosuvastatin.	In the DSS-treated colitis mice, Rosuvastatin significantly reduced disease activity index compared to untreated group, and levels of pro-inflammatory cytokines like IL-6 were also attenuated. In vitro part of study showed that Rosuvastatin treated cell culture showed lower levels of inflammatory mediators and ROS.	Shin et al. [55]
Fluvastatin, Atorvastatin, Pravastatin	Murine RAW264.7 macrophages cell line	RAW264.7 macrophages treated with the following for 24 h: Fluvastatin at 1.25, 2.5, 5 and 10 μM, Atorvastatin at 10, 25, 50 and 100 μM. and Pravastatin at 25, 50 and 100 μM.	Fluvastatin and Atorvastatin increased H2S levels by Akt signalling. Pravastatin did not show a significant effect. Fluvastatin and Atorvastatin increased expression of cystathionine γ-lyase (CSE). Fluvastatin reduced IL-1β and MCP-1 mRNA levels.	Xu et al. [56]
Pravastatin	Mouse Podocytes	Cells treated with 0.01, 0.03, 0.1, 0.3 and1 mM Pravastatin	Carboxymethyllysine (CML)-induced MCP-1 was downregulated by Pravastatin. Pravastatin prevented phosphorylated ERK in podocytes and NF-ĸB and Sp1 translocation.	Gu et al. [57]
Simvastatin	M1 macrophage model stimulated with IFN-γ and LPS	1.0, 2.5, 5.0 µmol/L of Simvastatin was cultured in M1 macrophages for 9 h	Simvastatin exhibited anti-inflammatory effects by stimulating M1 macrophages to switch to M2 phenotype.	Li et al. [58]
Simvastatin	Elastase-induced pulmonary emphysema in C57BL/6 mice	Mice were treated with 20 μg of Simvastatin or 200 μL of PBS every day for 2 weeks	Simvastatin reduced inflammation via reducing neutrophils and hydroxyproline levels	Takahashi et al. [59]
Atorvastatin	Murine RAW264.7 macrophages cell line	The cells were pre-treated with 0, 10, 20 and 40 mmol/L of Atorvastatin for 1 h. After, some cells were incubated with 40 mg/mL of ox-LDL for 24 h.	Atorvastatin downregulated ox-LDL-induced inflammatory cytokines, COX-2 expression, MAPK activation and IκB-α degradation.	Shao et al. [60]
Pravastatin, Simvastatin	Adult male cynomolgus monkeys	The monkeys consumed atherogenic diet for 12 months while receiving either no treatment, 40 mg/kg per day of pravastatin or 20 mg/kg per day of Simvastatin.	The statins reduced inflammation by lowering IL-1β levels	Sukhova et al. [61]

**Table 3:** Table of clinical trials investigating activity of inflammatory markers in different disease states.

Statin Type	Condition	Phase	Duration	Eligibility	Major Outcome	Inflammatory Markers	Clinical Trial ID	Comments
Simvastatin	Breast Cancer	2	24–48 weeks	Women aged ≥ 18 with early or advanced breast cancer, one healthy breast, not pregnant, non-smokers, non-drinkers, and not on any other medications (including cancer, hormonal, lipid-lowering, or antibiotics) for at least 3 months, with no significant health issues. Excludes men, women with bilateral breast cancer, those under 18, healthy volunteers, or those not meeting the above criteria.	Simvastatin significantly reduced total cholesterol, LDL, triglycerides, hsCRP, and estrone sulphate, particularly in postmenopausal women, indicating anti-inflammatory and anti-cancer potential.	hsCRP	NCT00334542 [62]	The researchers recorded that about 20% of the people in the trial underwent muscular-related issues after statin treatment.
Lovastatin	Breast cancer	2	6 months	Inclusion criteria: 18 to 65 year old. Must have a high risk of developing breast cancer with known mutation in BRCA2, or other high-risk mutation. Must have family history of breast cancer. ECOG performance status 0 and no known other health conditions. Alcohol consumption must not exceed 3 alcoholic drinks per week. Exclusion criteria: Men. Women aged below 18 and above 65. Women with no risk of developing breast cancer/mutation in BRCA2, or other high-risk mutation or family history. Have had invasive breast cancer in past 2 years. Other known health conditions, especially muscular or liver related. Taking other medications like tamoxifen or allergic to statins. Alcoholic or breastfeeding.	No significant biomarker modulation	C-reactive protein (CRP)	NCT00285857 [63]	30 women participated, with 27 completing the trial, hence the observation of no significant biomarker modulation may be due to the small sample size. [63]
Rosuvastatin	Breast cancer	2	4 weeks	Inclusion Criteria: 18 year or above women with Metastatic breast cancer at stage IV. ECOG performance at 0, 1, 2. Currently receiving endocrine therapy with or without HER2 for at least 6 weeks. No other significant health condition. Exclusion criteria: below 18 women with non-metastatic breast cancer or metastatic breast cancer not at stage IV. Women participating in other clinical trials. Women completed HER2 or other chemotherapy. Have used statins recently or are taking other specific medication that interacts with the treatment drug. Have other health issues, including cardio, liver or muscular related problems. Asian descent women. Women who are alcoholic, smoke or pregnant/breastfeeding.	NRP	C-reactive Protein (CRP)	NCT01299038	The sample size is too small for (19 participants), leaving room for possible statistical errors.
Simvastatin	Breast cancer	2	14 days	Inclusion criteria: 18 or older post-menopausal women. Have history of invasive breast cancer, currently taking anastrozole for breast cancer treatment. Overall good health and no severe allergy to the medication. Exclusion criteria: younger than 18, no history of invasive breast cancer. Have other medical conditions and allergic to medication treatment. Currently using other statins, hormone therapy or antibiotics. Alcohol consumption exceeds 3 drinks a day. Pregnant or breastfeeding.	NRP	Serum Estradiol levels	NCT00354640	Simvastatin was given with anastrozole
Atorvastatin	Prostate cancer	2	6 months	Inclusion criteria: 18 years to 120 years old men. Have prostate cancer that has recurred after surgery or treatment. Rising PSA levels indicating cancer reoccurrence. No other cancer. Overall good health and platelet count. Exclusion Criteria: Younger than 18 years old or older than 120 years old. Have a history of heart conditions. Currently using other medication, including anti-thrombotic medications and statins. Ongoing cancer infection.	NRP	NF-κB, IL-6, Prostaglandin E2	NCT01220973	Atorvastatin was given with celecoxib. Although the trial period was relatively long, the sample size was small, with 27 participants at baseline and 1 withdrawing before therapy.
Lovastatin	Melanoma	2	6 months	Patients eligible for enrolment comprised individuals in overall satisfactory systemic health presenting with multiple atypical melanocytic naevi or with completely resected stage I–II melanoma without adjuvant therapy within the preceding three months, using effective contraception if of reproductive potential, while excluding those with untreated or advanced (stage III–IV) melanoma, recent exposure to lipid-lowering agents, hypersensitivity to lovastatin or congeners, clinically significant comorbidities, or current pregnancy or lactation.	No significant results observed between Lovastatin group and placebo group.	C-reactive protein and RelA (a subunit of NF-κB)	NCT00462280 [64]	Possibly the reason for not achieving significant results might be due to the short duration of the trials [64].
Rosuvastatin	Chronic Obstructive Pulmonary Disease	2	12 weeks	Inclusion criteria: Stable COPD patients aged 40–80 years, COPD stages I to IV based on GOLD criteria, no COPD exacerbations within the last three weeks. Exclusion criteria: Other diagnosed lung diseases except certain bronchitis or mild bronchiectasis, significant cardiovascular diseases, severe hypertension, extreme obesity, uncontrolled diabetes, severe hypercholesterolemia, specific neuromuscular or renal conditions, abnormal liver function, pregnancy, substance abuse, recent statin use or reaction, recent malignancy, uncontrolled hypothyroidism, recent participation in other clinical trials, and use of medications interacting with Crestor.	No significant change in endothelial function or FEV1. Contradictory inflammatory response, marked by decreased CRP and increasing IL-6	CRP, IL-6	NCT00929734 [65]	The researchers published the trial (RODEO Trial, see section “Statins and COPD”) [65]. The contradictory inflammatory response may be due to the short trial period.
Atorvastatin	Rheumatoid arthritis	3	6 months	Inclusion criteria: Adults aged 18–65 years with rheumatoid arthritis. Exclusion criteria: Pregnant or lactating individuals, and those with hepatic or renal impairment.	Recruiting	DAS-28	NCT03770702	The study is still recruiting.
Simvastatin	Pre-diabetic + Hypercholesterolemia + CVS Risk	N/A	24 weeks	Adults aged 18–75 years with a BMI of 25–40 kg/m^2^ and pre-diabetes, defined by impaired glucose tolerance or fasting hyperglycaemia, were eligible, excluding individuals with triglycerides > 350 mg/dL, LDL cholesterol > 200 mg/dL, unstable blood pressure, cardiovascular, hepatic, or renal pathology, or current use of anti-inflammatory agents or other pharmacological modulators of lipid or glucose homeostasis.	The researchers observed reduction in total LDL cholesterol and apolipoprotein B levels. However, triglycerides only showed a decrease after combined therapy. Simvastatin reduced CRP levels, but no significant effect t was observed for IL-6 or TNF-α.	CRP, IL-6, TNF-α	NCT01103648 [66]	The researchers gave monotherapies of statin and ezetimibe but later gave combination therapy to both groups. Perhaps the reason the researchers obtained contradictory results for IL-6 and TNF-α may be due to the small sample size [66].
Simvastatin	Stable Angina	N/A	6 weeks	Inclusion criteria: Adults aged 18–80 years with stable angina and LDL cholesterol levels between 70 and 160 mg/dL. Exclusion criteria: Renal failure, current treatment with more than 20 mg of simvastatin, hepatic disease, inflammatory diseases, and age younger than 18 or over 80.	LDL-cholesterol was reduced by simvastatin; however, there was no significant differences in levels of hsCRP, ox-LDL, or sCD40L between the 2 treatment groups.	CRP, ox-LDL, MCP-1, sICAM-1, sCD40L, IL-6	NCT00474123 [67]	One group of participants were treated with Statin and Ezetimibe [68]. The short duration of the clinical trial may present a limitation when assessing the effects on the inflammatory markers, as it may take longer to show results with the treatment.
Simvastatin	Type 1 Diabetes	2	N/A	Inclusion criteria: Adults over 20 years old with Type I diabetes (onset before 20 years and on insulin therapy since diagnosis), with diabetes duration over 1 year and no clinical macrovascular complications. Exclusion criteria: HbA1c > 10% in the last year, use of Glucophage or thiazolidinediones. Rheumatoid arthritis, abnormal liver function, thyroid disorders or other significant health conditions. Using steroid therapy or anti-inflammatory drugs except low-dose aspirin. Abnormal blood count. Pregnant/smoking or lactating.	NRP	CRP, IL-6, IL-8, TNF-α, NF-κB, monocyte cells, CD40	NCT00441844	It was a randomized double-blind trial, hence the probability of bias is reduced, and the results hold more validity.
Atorvastatin	Atrial Fibrillation	4	1 year	Inclusion criteria: Elderly patients aged 68–82 years with atrial fibrillation, adequate oral anticoagulation therapy, and cholesterol levels between 4.5 mmol/L and 7 mmol/L. Exclusion Criteria: Indication for cholesterol-lowering treatment according to Dutch CBO-cholesterol guidelines (2004).	Atorvastatin significantly reduced the inflammatory markers and improved memory and cognition. MRI revealed les brain tissue loss in treatment group, particularly in hippocampus and amygdala.	Hs-CRP, IL-1RA. IL-9, IL-13, IL-17, IFNγ, IL-12, MIP-1β	NCT00449410 [69]	Atorvastatin was given along with Ezetimibe [70]. The areas with less tissue loss were associated with emotion as well, suggesting further research opportunity to investigate atorvastatin as an anti-inflammatory in emotional disorders.
Rosuvastatin	Non-familial hypercholesterolemia, physical inactivity	4	20 weeks	Eligible participants comprised adults aged 40–65 years with nonfamilial hypercholesterolaemia, total cholesterol exceeding 200 mg/dL, LDL concentrations above 130 mg/dL, sedentary lifestyle, and moderate-to-low ethanol consumption, whereas exclusion encompassed hepatic or renal pathology, acute inflammatory or infectious states, other clinically significant morbidities, administration of agents such as corticosteroids or alternative lipid-lowering pharmacotherapies, active tobacco use, involuntary or intentional body mass reduction exceeding 2 kg within the preceding six months, and surgical intervention within the prior three months.	Rosuvastatin reduced TLR-4 expression on monocyte cells and TLR-4 activity. As a result of reduced TLR-4 activity, it reduced downstream inflammatory cytokines like IL-6, IL-12 and TNF-α	TLR4, CD14, IL-6, TNF-α, hsCRP, B7-1	NCT00295373 [71]	Researchers published these results but also 2 more papers related to it [72]. The researchers also introduced a behavioural intervention (exercise). The use of inhibitors in this study gives more strength and reliability to statins effect on TLR-4 and its mediated processes.
Rosuvastatin	Heart Failure	2	3 months	Adults aged 18–75 years with chronic heart failure classified as NYHA stage III–IV, peak oxygen uptake < 20 mL/min/kg, left ventricular ejection fraction < 30%, left ventricular end-diastolic diameter > 55 mm, and stable pharmacotherapy for ≥4 weeks were eligible, whereas exclusion applied to individuals with hepatic transaminase elevation (GOT, GPT), increased serum creatinine, insulin-dependent diabetes mellitus, arterial hypertension, myopathic disorders or elevated CK, fibrate therapy, concomitant administration of CYP3A4-metabolized agents, or any additional pathology precluding study participation.	Rosuvastatin improved vascular health by increasing levels of VEGF (a growth factor for blood vessels) and decreased levels of oxidized LDL.	Ox-LDL	NCT00176332 [73]	Reduced oxidized LDL suggests possible anti-inflammatory action and needs further investigation [73].
Simvastatin	Septic Shock	4	N/A	Adults aged > 18 years with septic shock of <48 h’ duration were eligible, whereas exclusion applied to pregnancy, inability to tolerate enteral administration, anticipated survival < 72 h, recent exposure to simvastatin or other HMG-CoA reductase inhibitors, hypersensitivity to the investigational agent or structural analogues, known or suspected porphyria, elevated rhabdomyolysis risk, and haemorrhagic shock.	NRP	NA	NCT00450840	N/A
Atorvastatin	Crohn’s Disease	2	13 weeks	Adults aged 18–65 years with clinically confirmed Crohn’s disease, C-reactive protein > 2 mg/L in the absence of infection, and either fecal calprotectin > 250 mg/kg or CDAI > 150 were eligible, whereas exclusion applied to CDAI > 450 or prednisolone therapy exceeding 15 mg/day.	Atorvastatin reduced CXCL10 levels suggesting a decrease in CRP as they are correlated, therefore demonstrating atorvastatin’s anti-inflammatory effects.	CCL2, CCL4, CCL11, CCL13, CCL17, CCL22, CCL26, CXCL8, CXCL10, sP-selectin, sE-selectin, sICAM-3, thrombomodulin, CRP.	NCT00454545 [74]	The researchers published their results [75]. The trial only had 10 patients investigate the efficacy of the drug on, combined with the short trial period of 13 weeks. Hence, the trial should be replicated with higher number of participants and duration to achieve more reliable results.
Atorvastatin	Endometriosis	N/A	6 months	Premenopausal women aged 18–45 years providing written informed consent, exhibiting clinical manifestations of endometriosis for ≥3 months confirmed via laparoscopy or laparotomy within the preceding 4 months to 5 years (preferably with histological corroboration), with moderate pain symptoms such as dysmenorrhoea and no evidence of sexually transmitted infection, were eligible. Exclusion encompassed malignancy of the ovary, adrenal gland, endometrium, cervix, or breast; pregnancy or lactation; unexplained uterine or cervical hemorrhage; hormonal therapy within the prior 3 months (6 months for GnRH analogues); menstrual cycle irregularity > 35 days or secondary amenorrhoea > 3 months; chronic disorders involving the pelvic or abdominal cavity; uncontrolled type I or type II diabetes mellitus; venous thromboembolism or other contraindications to the investigational medicinal product; and continuous administration of CYP3A4 inhibitors.	NRP	Inflammatory status	NCT00675779	Atorvastatin was given either alone or in combination with oral contraceptives.
Atorvastatin	Dyslipidemia	4	12–24 weeks	Inclusion criteria: Adults aged 21–75 years with LDL-C > 130 mg/dL and HDL-C ≤ 45 mg/dL in men or ≤ 55 mg/dL in women. Exclusion criteria: History of hypersensitivity to any statin, niacin, or aspirin; diagnosis of diabetes or fasting glucose > 125 mg/dL; untreated hyper or hypothyroidism (unless treatment is stable); and other health, medication, and logistical criteria.	Atorvastatin reduced LDL-cholesterol and increases HDL-cholesterol. Both treatments reduced inflammation markers like hs-CRP and TNF-α, with a more pronounced effect seen with the combination therapy. No change in IL-6	hsCRP	NCT00194402 [76]	Atorvastatin was given with Slo-Niacin [77]. No difference in IL-6 was noted.
Atorvastatin	Heart Failure	4	6 months	Adults ≥ 18 years with mild-to-moderate heart failure (NYHA class II–III) of any etiology, documented left ventricular systolic dysfunction (LVEF < 45%) by echocardiography within 3 months prior to randomisation, clinically stable, receiving optimized HF pharmacotherapy unchanged for ≥4 weeks before enrolment, and exhibiting normal fasting total cholesterol were eligible. Exclusion criteria comprised concurrent lipid-lowering therapy, absolute or relative contraindications to statins, established chronic inflammatory disorders, or medical conditions necessitating anti-inflammatory or immunosuppressive treatment.	Atorvastatin reduced expression of PIIINP, Hs-CRP, and BNP. The most reduced expression observed was for PIIINP	hsCRP, TNF-α, IL-6	NCT00795912 [78]	Further investigations should be conducted on why PIIINP out of all was most reduced. [78]
Atorvastatin	Abdominal surgery	3	5 weeks	Male and female patients aged 25–55 years undergoing non-bowel abdominal surgery involving an abdominal incision under spinal anesthesia, weighing 50–120 kg, capable of comprehending and adhering to all study procedures, and providing written informed consent were eligible. Exclusion criteria encompassed bowel surgery, procedures under general anaesthesia, emergency interventions, high-risk infection laparoscopic surgery, pregnancy or lactation (or absence of reliable contraception), active malignancy, uncontrolled diabetes mellitus, untreated hypertension, psychotic disorders or other significant psychiatric impairment, any condition predisposing to non-compliance or adversely influencing treatment outcomes, and known hypersensitivity to the investigational product or its constituents.	NRP	TNF-α, CRP	NCT00902967	N/A
Atorvastatin	Sepsis	2	10 days	Patients aged ≥ 15 years with a clinical diagnosis of severe sepsis or septic shock established within the preceding 24 h were eligible, whereas exclusion applied to prior statin use within 30 days, definitive clinical indication for statin therapy, ongoing immunosuppressive treatment, elevated risk of rhabdomyolysis, AIDS diagnosis, inability to tolerate enteral administration, pregnancy, or anticipated survival < 48 h.	NRP	N/A	NCT00452608	N/A
Simvastatin	Cystic Fibrosis	1	28 days	Patients aged ≥ 10 years with cystic fibrosis who were clinically stable and had an FEV_1_ > 50% of predicted were eligible, whereas exclusion applied to hepatic pathology, *Burkholderia cepacia* infection, corticosteroid therapy, and symptomatic allergic rhinitis.	NRP	IL-6, IL-8, and NOS2 mRNA	NCT00255242	N/A
Atorvastatin	Dilated Cardiomyopathy	N/A	6 months	Patients aged ≥ 18 years with dilated cardiomyopathy (per ESC 2007 criteria), EF ≤ 40% confirmed by echocardiography, absence of significant coronary artery stenosis > 30% on cardiac catheterization, and provision of written informed consent were eligible. Exclusion criteria encompassed abnormal blood pressure, concomitant cardiac disorders or heart failure, current statin therapy, hepatic or renal dysfunction, uncontrolled diabetes mellitus, alcohol or substance misuse, pregnancy or lactation, recent surgical procedures, thyroid disease, and immune system disorders.	Atorvastatin reduced IL-6 and TNF-α. Atorvastatin also improved heart function by decreasing NT-proBNP, which increased in control group but decreased in atorvastatin group. The atorvastatin group were able to walk more and had fewer hospitalization rates.	TNF-α, IL-6, IL-10	NCT01015144 [79]	N/A
Simvastatin	Myocardial Infarction	4	4 weeks	Eligible population comprised individuals aged 40–70 years presenting within a temporal window of ≤24 h from the inception of symptomatology consistent with acute myocardial infarction, exhibiting electrocardiographic perturbations characterized by ST-segment elevation ≥ 1 mm in the limb-lead vector orientation or ≥2 mm in the precordial-lead vector orientation across two anatomically contiguous leads, in conjunction with corroborative biochemical evidence of myocardial cytolysis, namely elevated creatine kinase–MB isoenzyme and troponin concentrations. Exclusionary parameters encompassed any antecedent exposure to statin-class pharmacotherapeutics within the six-month interval preceding the index myocardial infarction event.	NRP	CRP	NCT00905905	One group was given statin with Ezetimibe.
Rosuvastatin	Abdominal Sepsis	2	3 days	Eligibility parameters encompassed male and female patients aged 18–80 years presenting with abdominal sepsis, wherein a definitive diagnosis of diffuse peritonitis was surgically established within ≤48 h of clinical progression, including cases precipitated by penetrating trauma from metallic projectiles or bladed weaponry with concomitant contamination of the peritoneal cavity. Additional inclusionary requirements comprised an APACHE II severity index ≥ 8 and formal consent to enrolment. Exclusionary parameters included prior or current administration of pharmacological agents such as statins or fibrates; end-stage impairment of hepatic or renal function; ongoing pregnancy; recent traumatic insult; documented hypersensitivity to statin-class compounds; or concurrent management in a healthcare facility external to the study site.	NRP	IL-6, IL-1β, TNF-α, CRP	NCT00357123	N/A
Pravastatin	Crohn’s Disease	N/A	6 weeks	Men and non-pregnant women aged 18–65 years, adhering to an adequate contraceptive regimen, with active Crohn’s disease defined by HBI > 5 or serum CRP above the upper reference limit, receiving a stable pharmacological regimen for ≥4 weeks with continuation planned for 6 weeks, and maintained on an unchanged azathioprine/6-MP or methotrexate dosage for ≥8 weeks prior to enrolment were eligible. Exclusion applied to individuals < 18 or >65 years, concurrent use of alternative statins or other lipid-lowering agents, hypersensitivity to statin-class drugs, pregnancy or lactation, clinically significant hepatic or renal functional impairment, severe Crohn’s disease–related complications or other major comorbidities, and participation in other investigational therapeutic protocols.	NRP	CRP, ESR	NCT00599625	N/A
Simvastatin	Myocardial Infarction	4	5 days	Patients aged 45–70 years presenting within ≤24 h of the onset of clinical manifestations consistent with acute myocardial infarction, exhibiting ST-segment elevation across two anatomically contiguous electrocardiographic leads, and demonstrating biochemical confirmation of myocardial injury through elevated CK-MB and troponin concentrations were eligible. Exclusion encompassed any prior statin exposure within the preceding six months.	The researchers found that early and higher doses of simvastatin were more affective. Simvastatin reduced levels of CRP, IL-2 and TNF-α, along with improving blood vessel function and reducing oxidative stress	CRP	NCT00906451 [80]	The trials utilized a good sample size of 125 patients, allowing more reliable statistical analysis [80].
Pitavastatin	Metabolic Syndrome	4	12 weeks	Hypercholesterolemic individuals aged ≥ 20 years were eligible, whereas exclusion applied to those currently receiving lipid-lowering pharmacotherapy, diagnosed with familial hypercholesterolaemia, exhibiting renal pathology, disorders of the hepatic, gallbladder, or biliary systems, or pregnancy.	Pitavastatin reduced CRP levels and lower levels of adiponectin.	hsCRP, soluble ICAM-1	NCT00444717 [81]	N/A
Atorvastatin	Surgery	N/A	48 h	Patients aged > 45 years scheduled for elective high-risk surgery as per POISE criteria, and suitable for baseline brachial artery ultrasonography prior to the procedure, were eligible. Exclusion criteria comprised absence of informed consent, pregnancy, contraindications to the brachial artery ultrasound protocol (including intolerance to 0.4 mg sublingual nitroglycerin), concurrent participation in a conflicting clinical investigation, or prior enrolment in STAR-VaS or STAR-VaS2.	NRP	CRP	NCT00967252	N/A
Atorvastatin + Simvastatin	N/A	4	6 weeks	Inclusion Criteria: Adults aged 18 years or older with low HDL-c cholesterol levels (<40 mg/dL for men, <50 mg/dL for women) or elevated LDL-c or non-HDL-c levels (for TG 200–500 mg/dL) requiring therapy per NCEP guidelines, with an identifiable primary care provider and a working phone number for follow-up. Exclusion Criteria: Under 18, unstable heart condition, liver problem/other significant health condition. Alcoholic, pregnant or breastfeeding. Taking other medication that can interfere with the study or participating in other studies.	NRP	hsCRP	NCT00736463	N/A
Rosuvastatin	Healthy	NA	NA	Healthy male, non-Asian individuals aged 19–39 years were eligible, whereas exclusion applied to those exhibiting elevated hepatic function indices, increased serum creatinine concentrations, or receiving anti-inflammatory or immunomodulatory pharmacotherapy.	NRP	NA	NCT00874757	N/A
Simvastatin	Type 2 Diabetes	NA	8 weeks	Inclusion Criteria: Adults aged 35–80 years with type 2 diabetes, HbA1c between 6.0% and 9.0%, and LDL-c > 100 mg/dL with no lipid-lowering treatment in the last six months. Exclusion Criteria: Refusal to provide informed consent.	NRP	NF-κB, IL-6, CRP	NCT01424891	One group was given Simvastatin in combination with Ezetimibe
Atorvastatin	Polycystic Ovary Syndrome	NA	6 months	Inclusion Criteria: Women aged 30–50 years diagnosed with PCOS (Rotterdam criteria) and using safe non-hormonal contraception. Exclusion Criteria: Use of cholesterol-lowering agents, antidepressants, oral cortisone medication, hormonal contraception, nursing, pregnancy, type 2 diabetes, liver disease, menopause, kidney or liver failure.	Atorvastatin improved lipid profile by decreasing LDL cholesterol and increasing HDL cholesterol. It reduced CRP levels significantly. Dehydroepiandrosterone sulphate (DHEA-S) decreased but Atorvastatin increased insulin resistance and had no effect on testosterone.	CRP	NCT01072097 [82]	More research needs to be conducted to find out the cause of the mixed effects on androgens
Rosuvastatin	Dietary selenium deficiency + dietary zinc deficiency	NA	4 months	Eligibility parameters encompassed adult and geriatric cohorts with angiographically substantiated coronary atherosclerotic pathology and hemodynamically stable angina pectoris, characterized by ≥70% luminal calibre reduction in at least one segment of a principal epicardial coronary conduit, or ≥50% diminution in the intraluminal calibre of the left main coronary artery. Exclusionary determinants incorporated the presence of cardiac complications or any severe systemic comorbid condition, including but not limited to endocrine disorders of thyroidal origin, hematological dyscrasias, congenital anomalies, autoimmune-mediated hepatopathies, end-stage renal insufficiency, malignant neoplasia, concomitant infectious states, osteoporotic bone demineralisation, or post-surgical physiological stress states. Patients were also excluded if receiving pharmacological agents such as antacid formulations, antimicrobial agents, micronutrient supplementation, ethanol ingestion, or maintaining active tobacco use.	Rosuvastatin was effective in lowering LDL cholesterol and triglycerides. Rosuvastatin alone or in combination therapy has no effect on the level of antioxidant, zinc or selenium mineral. However, it did reduce hs-CRP levels	hsCRP, IL-6	NCT01547377 [83]	One group was given rosuvastatin was co-treated with Zinc and Selenium [84]. Further studies need to be conducted with different dosages and population to reliably establish the possibility of using rosuvastatin in combination with zinc or selenium in conditions requiring antioxidant properties.
Atorvastatin	Inflammation	4	16 weeks	Inclusion Criteria: Adults aged 18 years and older who are normocholesterolemic. Exclusion Criteria: Individuals with cardiovascular disease or risk equivalents, malignancy, active alcohol abuse, contraindications to statins, interacting drugs, or chronic use of anti-inflammatory drugs.	No significant change in ENA-78 cytokine at the end of the 16-week period	ENA-78	NCT00361283	Although the trial had a relatively large sample size of 108 individuals, 81 of them completed the trial and 27 were lost to follow-up.
Simvastatin	Depression + Coronary Artery Disease + Acute Coronary Syndrome	1	20 weeks	Adults aged 18–60 years presenting with mild depressive symptomatology and elevated inflammatory indices (CRP > 2 mg/L) were eligible, whereas exclusion encompassed language or communication limitations, active substance misuse, clinically significant comorbidities such as hepatic or cardiovascular pathology, pregnancy, concurrent pharmacotherapy with potential to interfere with study parameters, or meeting established criteria for initiation of cholesterol-lowering therapy.	Terminated	CRP	NCT00208117	The researchers were planning to compare outcomes in patients given either Simvastatin, Sertraline or placebo. However, the trial was terminated due to researchers being unable to enrol participants.
Atorvastatin	Rheumatoid Arthritis	4	12 weeks	Adults aged ≥ 18 years meeting ACR classification criteria for rheumatoid arthritis, with disease duration ≥ 1 year, active disease status, and maintained on stable DMARD therapy for ≥3 months were eligible. Exclusion criteria comprised inability to provide informed consent, pregnancy or lactation, eligibility for or current use of lipid-lowering agents, documented hepatic pathology, elevated hepatic transaminases within the preceding two months, and recent exposure to hydroxychloroquine.	Atorvastatin reduced the inflammatory properties of HDL-cholesterol and hs-CRP. While there were improved, there was no significant improvement in the disease and ICAM-1.	hs-CRP, cytokine/ICAM-1	NCT00356473 [85]	Further investigation needs to be conducted as to why there was no improvement in disease or ICAM-1 levels.
Simvastatin	Diabetes + Dyslipidemia	NA	30 days	Inclusion Criteria: Adults aged 35–64 years with primary hyperlipidemia (total cholesterol > 200 mg/dL, triglycerides > 150 mg/dL), type 2 diabetes, and informed consent. For women: post-menopausal (>12 months), post-hysterectomy, or using mechanical contraception. Exclusion Criteria: Severe medical conditions like heart failure, kidney failure or cancer withing the last 5 years. secondary hyperlipidemia, morbid obesity (BMI > 40 kg/m^2^), alcohol or drug abuse, non-compliance, and specific laboratory abnormalities (elevated alanine transferase, creatine kinase, low hemoglobin, platelets, or white blood cells).	NRP	IL-1, TNF-α, IL-6, IL-10, hsCRP	NCT01101204	The researcher gave combined treatment of 10 mg Simvastatin, 1000 mg Metformin, and 100 mg Fenofibrate.
Simvastatin	Community-acquired pneumonia	3	30 days	Inclusion Criteria: Participants must be ≥60 years of age with a clinically and/or radiographically confirmed diagnosis of pneumonia. Exclusion Criteria: Exclusion criteria include the presence of severe sepsis or septic shock, hepatic cirrhosis (any etiology), acute coronary syndrome within the current admission or recent past, total serum cholesterol levels outside the reference safety range (marked hypercholesterolaemia or hypocholesterolaemia), ongoing therapeutic anticoagulation, current systemic corticosteroid therapy or other immunosuppressive pharmacotherapy, and declination of informed consent.	NRP	TNF-α, IFN-γ, CRP, PAI-1	NCT01651728	N/A
Simvastatin	Coronary Artery Disease	NA	24 h	Inclusion Criteria: Adults aged ≥ 18 years scheduled to undergo percutaneous coronary intervention (PCI) or diagnostic coronary angiography, with no documented history of statin intolerance or adverse reactions, and who have provided written informed consent. Exclusion Criteria: Exclusion criteria comprise: Severe acute coronary syndromes, including ST-segment elevation myocardial infarction (STEMI) at presentation. Documented hypersensitivity or intolerance to HMG-CoA reductase inhibitors. Unexplained myalgia or myopathy, or creatine kinase (CK) elevation suggestive of statin-associated muscle injury. Acute hepatic disease or persistent, clinically significant transaminase elevation. Concomitant therapy with cyclosporine, systemic azole antifungals, macrolide antibiotics, niacin, fibrates, nefazodone, or >1 quart/day grapefruit juice. Renal insufficiency (serum creatinine > 2.0 mg/dL). Known active malignancy. Severe left ventricular systolic dysfunction (LVEF < 25%). Patients not admitted to a coronary care unit or managed exclusively in an outpatient setting.	Terminated	hsCRP	NCT00588471	Study termination was necessitated by inadequate recruitment, resulting in failure to achieve the target enrolment required for statistical validity.
Atorvastatin	Atherosclerosis	4	3 months	Inclusion Criteria: Subjects aged 18 to 80 years with accumulation of FDG-PET in the carotid artery or aorta. Exclusion Criteria: LDL cholesterol level outside 120–180 mg/dL, current use of statins or fibrates, symptomatic coronary or cerebrovascular diseases, myocardial infarction or stroke within 6 months, recent vascular interventions or operations, poorly controlled diabetes or hypertension, neoplasms, and systemic inflammatory diseases.	NRP	IL-6, soluble ICAM-1, hsCRP	NCT00920101	The participants were given Atorvastatin along with lifestyle modifications (dietary).
NA	Coronary Artery Disease	2/3	12 weeks	Inclusion Criteria: Patients aged 20–90 diagnosed with coronary artery disease (CAD) with at least 50% stenosis in one major coronary artery confirmed by cardiac catheterization, and currently receiving statin therapy. Exclusion Criteria: Individuals under 18 years of age, pregnant women, and those taking antioxidant vitamin supplements are excluded from the study.	The levels of the antioxidant enzymes activities were significantly higher in coenzyme Q10 group. The levels of inflammatory markers were significantly lower after coenzyme Q10 supplementation.	hsCRP, TNF-α, IL-6, adiponectin	NCT01424761	The study published their results [86]. Although the researchers’ focus is on investigating Coenzyme Q10, all patients were undergoing statin therapy, suggesting possible anti-inflammatory benefits.
Atorvastatin	Acute Kidney Injury post Cardiac Surgery	NA	72 h	Inclusion Criteria: Adult patients (≥18 years) scheduled for elective coronary artery bypass grafting (CABG) employing cardiopulmonary bypass (CPB). Exclusion Criteria: Emergency or redo CABG procedures. Pre-existing renal impairment, defined as glomerular filtration rate (GFR) < 60 mL/min/1.73 m^2^. Hepatic dysfunction or failure. Documented myopathic disorders (hereditary, metabolic, or inflammatory). Left ventricular systolic dysfunction with LVEF < 40%. Known hypersensitivity or contraindication to HMG-CoA reductase inhibitors (statins). Pregnant or lactating women.	NRP	IL-6, IL-10, TNF-α, hsCRP	NCT01547455	The duration of the study was relatively short. With a sample size of only 96 participants, increasing the number of patients could help to enhance generalizability.
NA	Type 2 Diabetes + Atherosclerosis + Dyslipidemia	4	5 days	Inclusion Criteria: Adults with an age range of 35–80 diagnosed with type 2 diabetes mellitus and eGFR > 30 mL/min/1.73 m^2^, on statin therapy for at least 6 months at specified minimal dosages. Exclusion Criteria: Type 1 diabetes mellitus, chronic renal failure (eGFR < 30 mL/min/1.73 m^2^), recent acute diseases, chronic inflammatory diseases, active cancer, LDL cholesterol > 160 mg/dL, significant atherosclerosis, use of ezetimibe, fibrates, or niacin, EP hormone therapy, pregnancy, lactation, or inability to provide informed consent.	NRP	M1/M2 polarization, hsCRP	NCT01600690	This study investigates statin withdrawal. Duration of the study is relatively short with a small sample size of just 34 patients.
Atorvastatin	Bronchiectasis	4	6 months	Inclusion Criteria: Adults aged 18–75 years with a radiologically confirmed diagnosis of bronchiectasis. Clinically significant disease characterized by expectoration of mucopurulent or purulent sputum during periods of clinical stability. History of ≥2 infective pulmonary exacerbations per annum. Exclusion Criteria: Current tobacco use or smoking cessation within the preceding < 12 months, or cumulative smoking history exceeding 15 pack-years. Diagnosis of cystic fibrosis. Active allergic bronchopulmonary aspergillosis or active pulmonary tuberculosis. Poorly controlled asthma. Pregnancy or lactation. Documented hypersensitivity to HMG-CoA reductase inhibitors (statins). Active malignancy. Chronic hepatic disease. Established cardiovascular or cerebrovascular disease. Statin exposure within the preceding 12 months. Chronic macrolide antibiotic therapy. Chronic colonization with *Pseudomonas aeruginosa*.	Between 23 June 2011, and 30 January 2011, 82 patients were screened for inclusion in the study and 22 were excluded before randomisation. 30 individuals were assigned atorvastatin and 30 were allocated placebo. The change from baseline to 6 months in LCQ score differed between groups, with a mean change of 1.5 units in patients allocated atorvastatin versus −0.7 units in those assigned placebo (mean difference 2.2, 95% CI 0.5–3.9; *p* = 0.01). 12 (40%) of 30 patients in the atorvastatin group improved by 1.3 units or more on the LCQ compared with five (17%) of 30 in the placebo group (difference 23%, 95% CI 1–45; *p* = 0.04). Ten (33%) patients assigned atorvastatin had an adverse event versus three (10%) allocated placebo (difference 23%, 95% CI 3–43; *p* = 0.02). No serious adverse events were recorded.	Assessment of sputum neutrophil numbers and apoptosis; neutrophil activation in the airway, measured by sputum myeloperoxidase, free elastase activity, and interleukin 8 (a key neutrophil chemoattractant in bronchiectasis); systemic inflammation, measured by white-blood-cell count, concentrations of C-reactive protein, and the erythrocyte sedimentation rate; other markers of systemic inflammation, including amounts of interleukins 1β, 6, 8, 10, and 12p70, and tumour necrosis factor α.	NCT01299181	This study was referenced in the following research paper [87]. Had a small sample size of 60 participants
Atorvastatin	Type 2 Diabetes + Atherosclerosis	Withdrawn	12 weeks	Inclusion Criteria: Adults aged 35–80 years with a confirmed diagnosis of type 2 diabetes mellitus. Exclusion Criteria: Current or prior insulin therapy. Use of lipid-modifying agents, including statins, within the preceding 12 months. Women of childbearing potential. Active systemic inflammatory disease or vasculitic disorders. Symptomatic coronary artery disease. Symptomatic cerebrovascular disease. Clinically significant comorbidities, including active infection or malignancy. Hepatic dysfunction, defined as total bilirubin > 3.0 mg/dL or ALT > 2.5× the upper limit of normal (ULN). Renal dysfunction, defined as serum creatinine > 2.0 mg/dL.	NRP	hs-CRP, adiponectin, MCP-1, PAI-1, TNF-α, IL-6	NCT00932048	Study was withdrawn
Atorvastatin	Vascular calcification + Atherosclerosis + Dyslipidemia+ Inflammation	3	1 year	Inclusion Criteria: Adults aged 18–80 years. On maintenance bicarbonate hemodialysis, scheduled three times weekly. Willingness to participate, with provision of written informed consent. Exclusion Criteria: Presence of serious, life-limiting comorbidities, including active malignancy, active infection, or end-stage cardiac, pulmonary, or hepatic disease. Scheduled for living donor renal transplantation. Pregnancy or lactation. Recent (unspecified interval) coronary artery bypass grafting. Acute myocardial infarction or unstable angina. Absolute indication or contraindication for lipid-lowering therapy. Documented statin hypersensitivity. Use of lipid-modifying agents within the preceding 3 months. Uncontrolled hypothyroidism. Use of investigational drugs within the preceding 30 days. Inability to tolerate oral medications. Current treatment with systemic corticosteroids or other immunosuppressive agents. Alcohol or substance abuse. Elevated hepatic transaminases (ALT or AST > 3× ULN). Unexplained elevation of creatine kinase (CK). Inability to provide informed consent.	NRP	hsCRP	NCT00481364	N/A
Simvastatin	Asthma	3	8 weeks	Inclusion Criteria: Eligible participants will be male or female adults aged 18 to 70 years with forced expiratory volume in one second (FEV_1_) > 60% of the predicted value, as determined by standardized spirometric assessment, and requiring < 1000 µg/day of beclomethasone dipropionate or an equivalent inhaled corticosteroid dose for maintenance therapy. Exclusion Criteria: Subjects will be excluded if they have a documented history of chronic renal disease or a serum creatinine concentration > 2.0 mg/dL, or a history of chronic hepatic disease of any etiology. Additional exclusions encompass current pregnancy or lactation, prior exposure to statin pharmacotherapy, documented hypersensitivity or allergic reaction to statins, a history of statin-induced myositis, or any asthma exacerbation necessitating systemic corticosteroid therapy within the 3-month interval preceding study enrolment.	No Result Posted	Sputum eosinophil	NCT00792337	Participants were given Simvastatin and Budenoside together. Duration of the study is relatively short. The study also utilized a small sample size of 53 enrollments.
Atorvastatin	Obstructive Sleep Apnea	2	3 months	Inclusion Criteria: Eligible participants will comprise adult male and female subjects aged > 18 years with a confirmed diagnosis of obstructive sleep apnoea syndrome (OSAS), operationally defined by an apnoea–hypopnoea index (AHI) > 30 events/hour on polysomnography. Subjects must concurrently exhibit clinical arterial hypertension (ATH) of grade I or II, pharmacologically managed with monotherapy, with seated systolic arterial pressure (SAP) between 140 and 180 mmHg and diastolic arterial pressure (DAP) between 90 and 110 mmHg, measured under standardized conditions. Exclusion Criteria: Subjects will be excluded if they have a documented history of cerebrovascular accident or coronary ischaemic disease, chronic respiratory pathology (including, but not limited to, advanced chronic obstructive pulmonary disease or restrictive interstitial lung disorders), primary or secondary hypothyroidism, or are currently receiving statin therapy. Individuals requiring antihypertensive polypharmacy (i.e., >1 pharmacological agent for blood pressure control), pregnant or lactating women, and those with habitual ethanol consumption exceeding 3 units/day will be ineligible. Additional exclusions encompass concomitant treatment with pharmacological agents possessing significant cytochrome P450–mediated metabolic interactions or other clinically relevant drug–drug interactions, including azole antifungals (itraconazole, ketoconazole), antiprotease agents, fibrates, vitamin K antagonists, non-dihydropyridine calcium channel blockers (diltiazem, verapamil), macrolide antibiotics (erythromycin, clarithromycin), and cyclosporin. Hypersensitivity to investigational drug constituents, modification of existing therapeutic regimens within the preceding 3 months, severe excessive daytime somnolence with potential occupational safety hazards, or employment in safety-sensitive positions will also constitute exclusion criteria.	51 severe OSA patients were randomized. Key demographics for the study population were age 54 ± 11 years, 21.6% female, and BMI 28.5 ± 4.5 kg/m^2^. In intention to treat analysis, mean PAT difference between atorvastatin and placebo groups was 0.008 (−0.29; 0.28), *p* = 0.979. Total and LDL cholesterol significantly improved with atorvastatin. Systolic BP significantly decreased with atorvastatin (mean difference: −6.34 mmHg (−12.68; −0.01), *p* = 0.050) whereas carotid atherosclerosis and PWV were unchanged compared to the placebo group.	hsCRP, LTE4, 11-DHTXB2	NCT00669695	The following paper references the clinical study showing a direct link to trial’s findings [88]. Study was terminated as the interim analysis was performed without efficient results. Statin treatment can be studied for a longer duration and with a larger sample size for more efficient data.
Atorvastatin	TB-IRS+ Immune Reconstitution Inflammatory Syndrome + Immune Reconstitution Syndrome	2 + 3	7 days	Inclusion Criteria Adults aged ≥ 18 years. Confirmed HIV-1 infection by enzyme-linked immunosorbent assay (ELISA) or rapid HIV diagnostic test. Clinical diagnosis of paradoxical tuberculosis–associated immune reconstitution inflammatory syndrome (TB-IRIS). Currently receiving appropriate antiretroviral therapy (ART) and anti-tuberculosis (anti-TB) treatment. Ability to provide written informed consent. Exclusion Criteria Inability to tolerate or ingest oral medications. Concomitant treatment with chemotherapeutic agents, immunosuppressants, systemic corticosteroids, non-steroidal anti-inflammatory drugs (NSAIDs), or statins. Inability or unwillingness to attend scheduled clinical follow-up visits. Documented hypersensitivity or allergic reaction to NSAIDs, statins, or corticosteroids. Hepatic transaminases (ALT/AST) > 2 × upper limit of normal (ULN). History of myositis or myopathy. Clinical suspicion of anti-TB treatment failure. Current systemic azole antifungal therapy. Presence of serious comorbidities or opportunistic co-infections that, in the investigator’s judgement, could interfere with study participation. Minimal or subclinical IRIS manifestations. Pregnancy or lactation. Ongoing HIV therapy containing a protease inhibitor. Additional Exclusion Criteria for Randomization Arm A Life-threatening TB-IRIS manifestations. Uncontrolled diabetes mellitus. Renal impairment with glomerular filtration rate (GFR) < 60 mL/min. Uncontrolled congestive heart failure. History of bleeding diathesis or platelet count < 100,000/µL. Significant gastrointestinal bleeding or peptic ulcer disease. Prior systemic corticosteroid therapy for the current TB episode exceeding 48 h in duration. Pregnancy at the time of screening or randomization.	NRP	hsCRP	NCT01442428	The trial was terminated prior to enrolment owing to unforeseen natural disaster–related disruptions. Specifically, the 2011 Thailand flooding resulted in the destruction of the Good Manufacturing Practice (GMP)–compliant pharmacy facility designated for investigational product handling and storage. This event precipitated substantial operational delays, compounded by associated project timeline overruns and regulatory compliance obstacles, ultimately rendering continuation of the study infeasible.
Simvastatin	Cystic Fibrosis + Systemic Inflammation	1 + 2	12 weeks	Inclusion Criteria Adults aged ≥ 19 years with a confirmed diagnosis of cystic fibrosis (CF), established by characteristic clinical manifestations in conjunction with genotypic confirmation of class I or II CFTR mutations. Ability to provide written informed consent. Clinically stable at the time of enrollment, with no evidence of acute pulmonary exacerbation. Capacity and willingness to adhere to all study-related procedures and follow-up assessments. Exclusion Criteria Hypersensitivity or prior adverse reaction to simvastatin. Laboratory abnormalities at screening: ○ Aspartate aminotransferase (AST) or alanine aminotransferase (ALT) > 1.5 × upper limit of normal (ULN). ○ Creatine kinase (CK) > 1.5 × ULN. ○ Estimated glomerular filtration rate (eGFR) < 40 mL/min/1.73 m^2^. Receipt within the specified pre-enrollment intervals of: ○ Systemic or inhaled antibiotics. ○ High-dose ibuprofen therapy. ○ Dornase alfa, hypertonic saline, or aerosolized antimicrobial agents. Concomitant use of medications with clinically significant pharmacokinetic or pharmacodynamic interactions with simvastatin. Current or recent use of systemic corticosteroids or investigational agents. Evidence of major organ dysfunction or history of lung transplantation. Pregnancy or lactation, or unwillingness to employ reliable contraception for the duration of the study. Chronic administration of protocol-prohibited medications. Chronic airway colonization or active infection with *Burkholderia cepacia* complex.	NRP	IL-6, TNF, IL-1β, LPS, LBP, sCD14, EndoCAB, SP-D, CCL-18	NCT01092572	Study was withdrawn due to lack of funding.
Simvastatin	COPD	3	Week 12	Inclusion Criteria Adults aged 40 to 80 years with a confirmed diagnosis of stable chronic obstructive pulmonary disease (COPD), defined according to GOLD criteria. History of tobacco smoking, with current status as an ex-smoker. Post-bronchodilator forced expiratory volume in 1 s (FEV_1_) < 80% of the predicted value, measured according to ATS/ERS spirometry standards. Exclusion Criteria Presence of severe comorbidities, including but not limited to: ○ Advanced-stage malignancy (any histologic type). ○ Extensive pulmonary tuberculosis with residual parenchymal involvement exceeding one-third of total lung volume. ○ History of pneumonectomy or pneumoconiosis. ○ Left-sided cardiac failure or cardiomyopathy with left ventricular ejection fraction < 45%. ○ Any clinically significant cardiovascular disease (ischemic, valvular, or arrhythmic). ○ Diabetes mellitus requiring insulin therapy. ○ Hypercholesterolemia requiring pharmacologic intervention. ○ Chronic inflammatory disorders, including asthma, rheumatoid arthritis, pulmonary fibrosis, or autoimmune diseases. Pharmacologic exclusions: receipt of systemic corticosteroids, non-steroidal anti-inflammatory drugs, or statins within 3 months prior to enrollment.	NRP	Systemic Inflammation	NCT02070133	Short study duration yielded with a very small sample size of only 18 participants.
Simvastatin	COPD	4	6 weeks	Inclusion Criteria: Male or female patients aged 45–80 years with confirmed COPD (FEV1 30–80% predicted, FEV1/FVC < 0.7, salbutamol reversibility < 12%), supportive smoking history, able to attend regular clinic appointments, and willing to comply with study requirements. Females of childbearing potential must have a negative pregnancy test at screening and use contraception. Exclusion Criteria: Known hypersensitivity to statins or related medications, clinically significant liver function abnormality, hypercholesterolemia (≥6.5 mmol/L), pregnancy or breastfeeding without contraception, conditions deemed detrimental to the study by the investigator, recent exacerbation, significant hypoxia, lactose intolerance, specific medical therapies or conditions as listed.	Total cholesterol dropped in the active group. There was no significant change in aortic PWV between the active group and the placebo group (−0.7 m/s, *p* = 0.24). In those with aortic stiffness > 10 m/s (*n* = 22), aortic PWV improved in the active group compared with the placebo group (−2.8 m/s, *p* = 0.03). Neither systemic nor airway inflammatory markers changed	MMP-9, hsCRP, NO	NCT01151306 [89]	Duration of the study was short with a sample size of 70.
Atorvastatin	Atrial Fibrillation + Arrhythmia + Inflammation+ Endothelial Dysfunction	4	3 months	Inclusion Criteria Adults aged ≥ 18 years scheduled to undergo a clinically indicated left atrial catheter ablation for the management of atrial fibrillation (paroxysmal, persistent, or long-standing persistent), as determined by the treating electrophysiologist. Exclusion Criteria Active malignancy (histologically or clinically confirmed) at the time of screening. Diagnosed systemic inflammatory disorder (e.g., rheumatoid arthritis, systemic lupus erythematosus, vasculitis). Recent major medical event within the preceding 30 days, including: ○ Surgical intervention (elective or emergency). ○ Significant traumatic injury. ○ Acute myocardial infarction (STEMI or NSTEMI). Known contraindication to HMG-CoA reductase inhibitor (statin) therapy, including documented hypersensitivity or severe prior adverse reaction. Hepatic enzyme elevation (alanine aminotransferase [ALT] or aspartate aminotransferase [AST]) > 2 × the upper limit of normal at baseline screening. Current use of any statin, niacin, or fibrate therapy at the time of randomization.	No Results Posted	CRP	NCT00579098	N/A
Pravastatin	Schizophrenia + Schizoaffective disorders	4	12 weeks	Inclusion Criteria Male or female participants aged 18–68 years with a DSM-IV-TR (or DSM-5) diagnosis of schizophrenia (any subtype), schizoaffective disorder (any subtype), or schizophreniform disorder, confirmed by structured clinical interview. Documented sustained adherence to prescribed outpatient pharmacotherapy, including antipsychotic agents, as verified by clinical records, treating psychiatrist confirmation, or pharmacy refill history. Exclusion Criteria Inability or unwillingness to provide written informed consent in accordance with institutional review board (IRB) standards. Current substance use disorder or alcohol abuse (as defined by DSM criteria) within the preceding 6 months. Presence of clinically significant comorbid medical conditions, including: ○ Severe cardiovascular pathology (e.g., unstable angina, decompensated heart failure, recent myocardial infarction). ○ Renal insufficiency (serum creatinine > 1.5 mg/dL). ○ Severe hepatic impairment or active hepatocellular disease. ○ Anemia (hemoglobin < 11.0 g/dL). ○ History of severe traumatic brain injury. ○ Untreated primary myopathies or other clinically significant neuromuscular disorders. Psychiatric instability (e.g., hospitalization for psychosis or suicidality within the prior 3 months). Pregnancy, lactation, or unwillingness/inability to use highly effective contraception throughout study participation. Concomitant use of any prohibited pharmacological agents, including: ○ Anti-inflammatory drugs (e.g., daily aspirin, ibuprofen). ○ Thiazide diuretics. ○ Appetite suppressants or other weight loss agents. ○ Herbal supplements (e.g., St. John’s Wort). ○ Insulin therapy. ○ Colchicine. ○ Azole antifungals. ○ Macrolide antibiotics. ○ HIV protease inhibitors. Untreated thyroid disease. Known hypersensitivity to pravastatin or any of its excipients.	Increase in mean change in CRP was reported compared to placebo, but significance was not reported	CRP	NCT01082588 [90]	Duration of the study was short with a sample size of only 60 participants.
Lovastatin	Lung inflammation	1	24 h	Inclusion Criteria: Healthy individuals aged 19–44 years, any race or ethnicity, with FEV1 and FVC > 80% of predicted and oxygen saturation > 97% on room air. Must be able to lie supine in a PET scanner for ~2.5 h and fast for 6 h. Exclusion Criteria: Pregnancy, lactation, actively menstruating at randomization, history of tobacco or illicit drug use in the past year, current prescription medication use, increased risk of radiation exposure, participation in another investigational drug study, known allergies to trimethoprim/sulfamethoxazole, amoxicillin, bronchoscopy drugs, lovastatin, or rhAPC, fasting glucose > 150 mg/dL. Additional exclusions for rhAPC and lovastatin use include recent internal bleeding, hemorrhagic stroke, severe trauma, anticoagulant or thrombolytic therapy, bleeding disorders, liver disease, certain medications, and specific lab abnormalities.	There was a statistically significant decrease in K(i) in the lovastatin-treated group that was not seen in the placebo-treated group, suggesting attenuation of inflammation by lovastatin treatment despite a small decrease in BAL total leukocyte and neutrophil counts that was not statistically significant. No significant decrease in K(i) was observed in the rhAPC-treated group, correlating with a lack of change in BAL parameters and indicating no significant anti-inflammatory effect with rhAPC.	Change in net influx constant (Kᵢ)—a quantitative index of [^18^F]fluorodeoxyglucose ([^18^F]FDG) uptake—in the right lung at 24 h post–lipopolysaccharide (LPS) instillation, as determined by Patlak graphical analysis of dynamic positron emission tomography (PET) imaging data.	NCT00741013 [91]	The study has a very short duration and has a very small sample size of 22 participants.
Atorvastatin	Atrial Fibrillation	3	12 months	Inclusion Criteria: Patients aged ≥ 18 years with a clinical diagnosis of atrial fibrillation/flutter (ECG documented) and able to swallow pill form of drug. Exclusion Criteria: Patients < 18 years, enrolled in another trial, with paroxysmal atrial fibrillation, hemodynamic instability, recent atrial fibrillation ablation, contraindications for anticoagulation, severe valvular heart disease, single lead ICD, unstable angina, NYHA Class IV heart failure, hyperthyroidism, uncontrolled hypertension, limited life expectancy (<1 year), recent statin use, coronary artery disease requiring statin therapy, implanted arrhythmia management devices, no telephone access, illicit drug or alcohol abuse, atorvastatin hypersensitivity, pregnancy, sexually active females not on contraception, nursing mothers, chronic liver disease, severe renal disease, inflammatory muscle disease, CK > 3 times ULN, or concurrent use of cyclosporine, fibrates, or high-dose niacin.	Although no significant effect was seen on oxidative stress, 2 of 4 inflammatory markers, IL-6 (adjusted OR: 0.59, 95% CI: 0.35–0.97, *p* = 0.04) and hs-CRP (adjusted OR: 0.59, 95% CI: 0.37–0.95, *p* = 0.03) were significantly lowered with atorvastatin. Cholesterol levels significantly decreased with atorvastatin (*p* = 0.03).	Serum oxidative stress markers (ratios of oxidized to reduced glutathione and cysteine, derivatives of reactive oxygen species, isoprostanes) and inflammatory markers (high-sensitivity C- reactive protein [hs-CRP], interleukin-6 [IL-6], interleukin-1β[IL-1β], tumour necrosis factor α [TNFα])	NCT00252967 [92]	Study was terminated due to insufficient power to show therapy difference at interim analysis [92].
Atorvastatin	Arterial Occlusive Disease + Intermittent Claudication + Insulin Resistance	2 + 3	1 h	Inclusion Criteria Eligible participants must meet all of the following: Age: 40 to 85 years, inclusive. Clinical Diagnosis: Documented symptomatic intermittent claudication of at least 6 months’ duration, attributable to peripheral artery disease. Ankle–Brachial Index (ABI): Resting ABI ≤ 0.90 measured in the index limb under standardized conditions. Functional Limitation: Maximal treadmill walking time (graded protocol) between 1 and 20 min at baseline testing. Exercise-Induced Hemodynamic Response: ≥20% reduction in ABI post-treadmill exercise compared with resting value. Lipid-Lowering Drug Status: If previously on statin therapy, completion of a minimum 4-week washout period prior to initial physiological and biochemical assessments. Exclusion Criteria Participants will be excluded if any of the following apply: Recent Major Cardiovascular Events: Myocardial infarction or coronary artery bypass grafting within the preceding 6 months. Recent Peripheral Intervention: Lower extremity revascularization procedure within the preceding 6 months. Recent Cerebrovascular Events: Transient ischemic attack or ischemic stroke within the preceding 6 months. Pregnancy: Current pregnancy or intention to become pregnant during the study period. Uncontrolled Hypertension: Resting systolic blood pressure > 180 mmHg and/or diastolic blood pressure > 100 mmHg, despite medical therapy. Renal Dysfunction: Serum creatinine > 2.5 mg/dL. Hepatic Dysfunction: Serum aspartate aminotransferase (AST) or alanine aminotransferase (ALT) > 3 × upper limit of normal (ULN). Skeletal Muscle Injury/Disorder: Serum creatine kinase > 5 × ULN. Drug Hypersensitivity: Documented allergy or intolerance to HMG-CoA reductase inhibitors (statins). Diabetes: Insulin-requiring type 2 diabetes mellitus. Prohibited Concomitant Medication: Current use of thiazolidinediones.	Compared with healthy subjects, PAD subjects had higher levels of circulating TNF-α (*p* < 0.0001), CRP (*p* = 0.003), sICAM (*p* < 0.0001), and IL-6 (*p* < 0.0001). Expression of both IL-6 (*p* = 0.024) and CD36 (*p* = 0.018) was greater in PAD subjects than in healthy subjects. Among subjects with PAD, higher gene expression of TNF-α was associated inversely with MWT (*p* = 0.01). MWT was also associated inversely with greater levels of circulating TNF-α (*p* = 0.028), CRP (*p* = 0.024), IL-6 (*p* = 0.03), and sICAM (*p* = 0.018).	NA	NCT00153166 [93]	The study was conducted for a very short duration of time.
Simvastatin	Atrial Fibrillation	2	6 months	Inclusion Criteria: Patients aged 18 years and older with paroxysmal atrial fibrillation (PAF) (≥3 episodes each > 15 min in length) over 6 months, on stable antiarrhythmic drug therapy, and with a life expectancy > 1 year. Exclusion Criteria: Patients with PAF due to a reversible cause, chronic inflammatory conditions, other medical conditions requiring statin therapy, on amiodarone or verapamil, elevated CK or ALT, life expectancy < 1 year, TAVN ablation, geographic isolation, or inability to give informed consent.	NRP	CRP, oxidative stress levels	NCT00321802	Small sample size of only 40 participants
Fluvastatin	Ageing, Inflammation	2	24 months	Inclusion Criteria: Patients aged 60–75 years, physically active less than 3 times a week, and able to provide written informed consent. Exclusion Criteria: Patients with heart disease requiring treatment, treatment with beta-receptor antagonists, statins, immunosuppressive drugs, or anti-inflammatory drugs, underlying hematological disease, alcohol or drug abuse, diabetes mellitus, recent study participation (within 30 days), known intolerance to the active agent, active liver disease, unexplained persistent elevation of serum transaminases or cholestasis, existing myopathy, pregnancy or nursing, absence of an ophthalmological examination within 12 months prior to inclusion, or known cataract.	NRP	Inflammatory parameters	NCT01045512	The study was terminated due to insufficient recruitment of participants.
Rosuvastatin	HIV Infection + Cardiovascular disease	2 + 3	3 months	Inclusion Criteria Adults (≥18 years) with confirmed HIV-1 infection, receiving stable combination antiretroviral therapy (cART) for ≥6 months prior to enrolment. Documented plasma HIV-1 RNA < 50 copies/mL within the screening window. Karnofsky Performance Status (KPS) score > 70 at screening. Laboratory parameters meeting all of the following thresholds: ○ Absolute neutrophil count (ANC) > 750 cells/mm^3^. ○ Hemoglobin ≥ 8.0 g/dL. ○ Platelet count ≥ 50,000/mm^3^. ○ Alanine aminotransferase (ALT) and aspartate aminotransferase (AST) < 2.5 × the upper limit of normal (ULN). ○ Fasting plasma glucose < 126 mg/dL. ○ Thyroid-stimulating hormone (TSH) < 3.0 mIU/L. ○ High-density lipoprotein cholesterol (HDL-C) < 50 mg/dL for men or < 55 mg/dL for women. ○ Low-density lipoprotein cholesterol (LDL-C) ≤ 130 mg/dL. ○ Estimated creatinine clearance (e.g., Cockcroft–Gault) > 50 mL/min. Willingness to be assigned to rosuvastatin therapy or to an observational control arm for a minimum of 3 months. For women of reproductive potential: agreement to use effective contraception during the intervention period. Exclusion Criteria Prior cardiovascular events (including myocardial infarction, stroke, or revascularisation procedures). Acute illness or active AIDS-defining opportunistic infection within 30 days prior to screening. Significant chronic comorbidities including, but not limited to, diabetes mellitus, clinically relevant autoimmune disease, or endocrinopathies. Positive serology for hepatitis B surface antigen or hepatitis C antibody. Evidence of hepatic decompensation or clinical liver failure. Systemic glucocorticoid therapy within the preceding 30 days. Current use of any lipid-lowering pharmacotherapy. Receipt of an HIV vaccine or other investigational agents within the protocol-defined washout period. Pregnancy or lactation. Active malignancy within the past 5 years (excluding non-melanoma skin cancer). Severe, uncontrolled hypertension. Current use of postmenopausal hormone replacement therapy. Known hypersensitivity to rosuvastatin or any excipients in its formulation. Active substance use disorder (drug or alcohol dependence) likely to impair adherence. Any acute or chronic medical condition deemed by the investigator to compromise study participation or data integrity. Concurrent use of lopinavir/ritonavir.	NRP	Primary and Secondary Biomarker Endpoints High-Sensitivity C-Reactive Protein (hsCRP): Quantitative serum concentrations determined via high-sensitivity immunoassay as a systemic inflammatory marker. Mitochondrial-Specific Oxidative DNA Damage: Measurement of mitochondrial DNA–localized 8-oxo-2′-deoxyguanosine (mt-specific 8-oxo-dG) via liquid chromatography–tandem mass spectrometry (LC-MS/MS) or equivalent high-specificity detection methodology. Oxidative Phosphorylation (OXPHOS) Bioenergetic Enzyme Activity: ○ Complex I (NADH:ubiquinone oxidoreductase) activity, assessed spectrophotometrically in isolated mitochondria or enriched fractions from peripheral blood mononuclear cells (PBMCs). ○ Complex IV (cytochrome c oxidase) activity, determined via polarographic or spectrophotometric methods under standardized conditions. T Lymphocyte Activation Markers: ○ CD38 expression quantified as mean fluorescence intensity (MFI) and/or percentage of CD3^+^ T cells expressing CD38. ○ CD69 expression on CD3^+^ T cells, measured as MFI and/or frequency via multiparameter flow cytometry. Monocyte Activation Markers: ○ CD16 (FcγRIII) expression on CD14^+^ monocytes, quantified by MFI/percentage positive cells. ○ CD69 expression on CD14^+^ monocytes, determined analogously by multiparameter flow cytometry.	NCT00986999	Study was terminated and no results were obtained due to poor enrollment of participants.
Atorvastatin	Renal Insufficiency (Graft Donor Kidney)	3	12 months	Inclusion Criteria Individuals formally accepted as living kidney donors following standard donor evaluation protocols. Voluntary enrolment with provision of written informed consent prior to any study-specific procedures. Demonstrated adherence to the atorvastatin regimen, defined as ≥85% compliance with prescribed dosing during the study treatment period. Exclusion Criteria Documented hypersensitivity or allergic reaction to atorvastatin or any excipient in the formulation. Active tobacco use at the time of screening, irrespective of frequency or duration.	NRP	CRP, IL-6, TNF-α	NCT02355704	Small sample of 48 participants was used.
Atorvastatin	Atherosclerotic Carotid Disease + Atheroma + Atherosclerosis	NA	12 weeks	Inclusion Criteria Presence of carotid atherosclerotic plaque demonstrated as positive on Sinerem^®^-enhanced magnetic resonance imaging (MRI), with interpretation and confirmation performed by a consultant neuroradiologist. Either *statin-naïve* at screening or receiving a stable regimen of an approved statin at a fixed dose for a minimum of four weeks prior to the screening visit. Exclusion Criteria Clinical requirement for any non-statin lipid-lowering pharmacotherapy (e.g., ezetimibe, fibrates, PCSK9 inhibitors). Documented history of statin intolerance. Chronic viral hepatitis (B or C) or laboratory/clinical evidence of hepatic dysfunction. Renal impairment, defined as serum creatinine concentration exceeding 2.5 mg/dL. History of myopathy or any inflammatory myopathic disorder. Known hypersensitivity or allergic reaction to dextran or iron salts. Contraindications to MRI scanning, including implanted ferromagnetic devices or severe claustrophobia unamenable to sedation. Scheduled carotid endarterectomy or any carotid intervention within 10 weeks of the screening visit. Doppler ultrasonographic evidence of carotid luminal stenosis < 40%.	Twenty patients completed 12 weeks of treatment in each group. A significant reduction from baseline in USPIO-defined inflammation was observed in the 80 mg group at both 6 weeks (DeltaSI 0.13; *p* = 0.0003) and at 12 weeks (DeltaSI 0.20; *p* < 0.0001). No difference was observed with the low-dose regimen. The 80-mg atorvastatin dose significantly reduced total cholesterol by 15% (*p* = 0.0003) and low-density lipoprotein cholesterol by 29% (*p* = 0.0001) at 12 weeks.	USPIO-enhanced MRI-defined inflammation	NCT00368589	The following paper is linked to the clinical trial [94]. Short duration of study and a small sample size of 47
Rosuvastatin	Carotid Artery Plaque + Ankylosing Spondylitis + Rheumatoid Arthritis	NA	18 months	Inclusion Criteria: Men and women aged 35–80 with RA, AS, or other inflammatory arthritis forms, having cholesterol plaques in the carotid artery as shown by ultrasound, and who have given informed consent. Exclusion Criteria: Concomitant statin treatment, chronic irregular heart rhythm (e.g., atrial fibrillation), contraindications to statins (e.g., liver disease, statin-induced myopathy, hypersensitivity), raised creatinine, pregnancy or breastfeeding, lack of contraceptives in fertile women, cyclosporine treatment, interaction-prone medications, uncontrolled hypothyroidism, low creatinine clearance, secondary hyperlipidemia, uncontrolled diabetes, severe heart failure, gastrointestinal diseases affecting absorption, cancer, severe psychiatric illness, life-threatening arrhythmias, alcohol abuse, and participation in other studies.	No significant changes in inflammatory markers	ESR, CRP	NCT01389388 [95,96,97,98,99]	N/A
Atorvastatin	Type 2 Diabetes + Hypercholesterolemia	4	30 days	Inclusion Criteria Hypercholesterolemic cohort: Individuals of white racial background, male or female, with clinically established *polygenic hypercholesterolemia* defined by low-density lipoprotein cholesterol (LDL-C) concentrations exceeding 160 mg/dL, capable of providing written informed consent. Type 2 Diabetes Mellitus (T2DM) cohort: Individuals of white racial background, male or female, meeting the American Diabetes Association diagnostic criteria for T2DM, maintained on acetylsalicylic acid (ASA) 100 mg/day for a minimum of 30 consecutive days prior to enrollment, and capable of providing written informed consent. Exclusion Criteria Hypercholesterolemic cohort: Clinical or biochemical evidence of hepatic insufficiency; advanced renal pathology; concomitant diabetes mellitus; arterial hypertension; prior history or current clinical evidence of myocardial infarction or other atherothrombotic disorders; diagnosed autoimmune disease; active or historical malignancy; recent or ongoing infectious processes; pharmacologic exposure to nonsteroidal anti-inflammatory drugs (NSAIDs), lipid metabolism–modulating agents, or vitamin supplementation within the exclusionary window. T2DM cohort: Acute vascular events within the preceding 3 months; diagnosis of type 1 diabetes mellitus; serum creatinine concentration > 2.5 mg/dL; active infection or malignant disease; clinically significant cardiac arrhythmia or congestive heart failure; pharmacologic exposure to NSAIDs, vitamin supplementation, or non-ASA antiplatelet agents (e.g., clopidogrel) within 30 days prior to enrollment.	The atorvastatin-assigned group showed a significant and progressive reduction in urinary isoprostanes and serum Nox2, along with inhibition of platelet recruitment, platelet isoprostanes, Nox2, Rac1, p47(phox), and protein kinase C, starting 2 h after administration. Platelet phospholipase A(2) and thromboxane A(2) significantly decreased, and vasodilator-stimulated phosphoprotein and nitric oxide increased after 24 h. Low-density lipoprotein cholesterol decreased significantly after 72 h and further declined after 7 days. No changes were observed in the Mediterranean diet group. In vitro experiments demonstrated that atorvastatin dose-dependently inhibited platelet Nox2 and phospholipase A(2) activation, along with inhibition of platelet recruitment, platelet isoprostanes, and thromboxane A(2), and increased vasodilator-stimulated phosphoprotein and nitric oxide.	Oxidative stress, as assessed by serum Nox2 and urinary isoprostanes, and platelet activation, as assessed by platelet recruitment, platelet isoprostanes, and thromboxane A(2), platelet Nox2, Rac1, p47(phox), protein kinase C, vasodilator-stimulated phosphoprotein, nitric oxide, and phospholipase A(2)	NCT01322711 [100]	Brief study period, hence needs further research and follow up. Has a small sample size of only 60 participants.
Simvastatin	Atherosclerosis	NA	3 months	Inclusion Criteria Adults aged 30–80 years. Evidence of carotid atherosclerosis detected by duplex ultrasonography, or vascular ^18F-fluorodeoxyglucose (FDG) uptake identified incidentally during FDG-PET performed for cancer screening. Exclusion Criteria Active inflammatory disorders. Dyslipidemia currently managed with lipid-lowering pharmacotherapy. Uncontrolled diabetes mellitus. Diagnosis of vasculitis. Symptomatic coronary artery disease or cerebrovascular disease. Known systemic pathology, including but not limited to: ○ Chronic hepatic disease. ○ Chronic renal disease. ○ Hematopoietic disorders. ○ Malignant neoplasms.	^18F^-fluorodeoxyglucose positron emission tomography (FDG-PET) identified 117 FDG-avid atheromatous plaques in the simvastatin-treated cohort and 123 in the dietary-intervention cohort at baseline. Pharmacologic intervention with simvastatin, but not dietary modification alone, elicited a statistically significant attenuation of plaque FDG uptake, reflected by a reduction in standardized uptake values (SUVs) (*p* < 0.01). Simvastatin therapy achieved a 30% decrement in low-density lipoprotein cholesterol (LDL-C) (*p* < 0.01) and a 15% increment in high-density lipoprotein cholesterol (HDL-C) (*p* < 0.01), whereas no significant modulation of LDL-C or HDL-C was observed in the diet-only group. Within the simvastatin arm, the magnitude of SUV reduction exhibited a robust positive correlation with HDL-C elevation (*p* < 0.01), but no statistically significant association with the degree of LDL-C reduction.	hsCRP, plaque inflammation	NCT00114504 [101,102]	The following papers have referenced the trial. The study has a small sample size of only 43 participants.
Simvastatin	Rhinosinusitis	2	60 days	Inclusion Criteria Male and female participants aged 18–65 years. Diagnosis of chronic rhinosinusitis (CRS), with or without nasal polyposis, persisting for ≥ 6 months. Scheduled for functional endoscopic sinus surgery (bilateral total ethmoidectomy). Documented failure of conventional post-surgical medical therapy. Exclusion Criteria Diagnosis of cystic fibrosis. Presence of primary or acquired immunodeficiency disorders. Diagnosis of diabetes mellitus. Current anticoagulant therapy or known coagulation/bleeding disorders. Use of systemic corticosteroids or antibiotics within 30 days prior to enrolment. History of sinonasal surgery within the preceding 6 months. Contraindications to statin therapy, including but not limited to: ○ Pregnancy or lactation. ○ Excessive alcohol consumption. ○ Chronic liver disease or severe renal impairment. ○ Myopathy or known hypersensitivity to statins. ○ Elevated serum transaminases above the upper limit of normal.	NRP	IL6, IL8, IL10, TNF	NCT01771198	The study was terminated due to lack of efficacy with one month of treatment.
Atorvastatin, Simvastatin, Pravastatin, Fluvastatin, Lovastatin	Carotid Atherosclerosis	Withdrawn	6 months	Inclusion Criteria: Adult patients (>21 years old) with diagnosed coronary artery disease or suspected cerebrovascular accident. Patients either having their statin dose or LDL lowering therapy increased to a high dose or maintained at a standard dose. Exclusion Criteria: Severe claustrophobia, implanted ferromagnetic materials unsuitable for MRI, anticipated carotid stenting or surgery within 6 months, inability to lie flat for 1 h, need for supplemental oxygen, pregnancy, and inability to follow up for 6 months.	NRP	Inflammatory Markers	NCT00388843	No results published with the study being withdrawn.
Atorvastatin	Vascular Disease	2	12 weeks	Inclusion Criteria: Adults aged 18–75 years with angiographically, ultrasonographically, or otherwise clinically documented atherosclerotic cardiovascular disease, presenting with fasting low-density lipoprotein cholesterol (LDL-C) concentrations between 70 and 130 mg/dL despite ongoing therapy with a stable regimen of low- to moderate-intensity statin for a protocol-defined pre-enrolment period. Eligible participants must exhibit a body mass index (BMI) within the range of 18–37 kg/m^2^, possess the ability to swallow oral solid dosage forms, and demonstrate adequate tolerance for all study-related interventions, including invasive or imaging-based procedures, administration of iodinated contrast agents, and trial pharmacotherapies. Exclusion Criteria: Documented statin intolerance or contraindication to continued statin therapy; clinically significant renal impairment, defined as a serum creatinine concentration > 1.5 mg/dL; known chronic viral hepatitis (e.g., hepatitis B or C) or biochemical/laboratory evidence of hepatic dysfunction; history of major infectious illness necessitating hospitalization or parenteral antimicrobial therapy within 2 months prior to initiation of study treatment.	Treatment with the p38 mitogen-activated protein kinase inhibitor BMS-582949 failed to elicit a statistically significant attenuation of arterial inflammation when compared with placebo, as evidenced by the change in target-to-background ratio (ΔTBR index: 0.10; 95% confidence interval [CI]: −0.11 to 0.30; *p* = 0.34) and in active slices (ΔTBR_(AS): −0.01; 95% CI: −0.31 to 0.28; *p* = 0.93). Similarly, there was no significant effect on high-sensitivity C-reactive protein (hs-CRP) concentrations (median percentage change [interquartile range, IQR]: 33.83% [153.91] for BMS-582949 vs. 16.71% [133.45] for placebo; *p* = 0.61). By contrast, statin intensification was associated with a statistically significant reduction in systemic inflammation, as reflected by hs-CRP (median % change [IQR]: −17.44% [54.68] vs. 16.71% [133.45] for placebo; *p* = 0.04), and a concomitant reduction in arterial inflammation within active slices (ΔTBR_(AS) = −0.24; 95% CI: −0.46 to −0.01; *p* = 0.04).	hsCRP, MMP9, IL-6, TNF-α	NCT00570752 [103]	Short study duration and small sample size of 72.
Pravastatin	HIV-1 Infection	Withdrawn	24 weeks	Inclusion Criteria: Adults aged > 18 years with confirmed *HIV-1* infection, demonstrating willingness to defer initiation of antiretroviral therapy (ART) and/or lipid-lowering therapy with statins for the study duration. Participants must present with a peripheral CD4^+^ T-lymphocyte count > 500 cells/mm^3^ and have no prior ART exposure exceeding 10 cumulative days, with the exception of short-course regimens administered for post-exposure prophylaxis, prevention of mother-to-child transmission, or other protocol-permitted circumstances. No lipid-lowering pharmacotherapy within 60 days and no ART within 30 days of enrolment are permitted. Eligibility requires capacity to provide written informed consent, absence of exclusionary medical or neuropsychiatric conditions, and fulfilment of protocol-defined laboratory thresholds for haematologic, hepatic, and renal function. Cardiovascular risk must meet one of the following criteria: (i) Framingham Risk Score (FRS) ≥ 10%, or (ii) FRS ≥ 6% in the context of high-sensitivity C-reactive protein (hsCRP) > 3.0 mg/L. Participants must have no exclusionary *HIV-1* resistance mutations, must complete a pre-entry flow-mediated dilation (FMD) assessment, and—if female and of reproductive potential—must have a negative serum or urine pregnancy test and commit to effective contraception. All candidates must demonstrate capacity to complete the neuropsychological test battery. Exclusion Criteria: Pregnancy or lactation; documented hypersensitivity or intolerance to study drugs; established cardiovascular disease; uncontrolled type II diabetes mellitus; hepatic cirrhosis; chronic systemic inflammatory or autoimmune disorders; recent (within protocol-defined intervals) major infection, severe illness, or trauma necessitating hospitalization; febrile illness or acute intercurrent disease at screening; New York Heart Association (NYHA) class III/IV congestive heart failure; uncontrolled hypertension; untreated hypothyroidism; active malignancy; active CNS infection, neoplasm, or other neurological disorder impacting cognition; failure to maintain ≥ 8 h of fasting and abstinence from tobacco use or exercise prior to protocol assessments; anticipated initiation/cessation of smoking or major dietary/exercise modifications during the trial; active substance use disorder; psychiatric illness likely to impair adherence; exposure to hormonal anabolic agents or immunomodulators within 60 days; receipt of routine prophylactic or therapeutic vaccinations within 7 days of study procedures; high-dose supplementation with antioxidant vitamins E or C; systemic glucocorticoid therapy above physiologic replacement doses; concurrent hepatitis C pharmacotherapy; or investigational agents administered within 90 days prior to enrolment.	NRP	IL-6, hsCRP	NCT01515813	The study was withdrawn as the trial is under revision.
Atorvastatin	Septic Shock	NA	28 days	Inclusion Criteria: Clinical disease of septic shock, aged 18 years and above, admitted to ICU. Exclusion Criteria: Previous statin-induced myopathy or hypersensitivity reaction, liver transaminases greater than 2.5 times the normal limit, chronic liver disease, pregnant or lactating mothers.	Seventy-three septic shock patients with 36 and 37 included in the atorvastatin and placebo group, respectively. Both groups were equally matched. Twenty-eight-day mortality, event-free days, lipid profile, and adverse effects were also not significantly different between groups. Reduced levels of IL-1, IL-6, TNF-α, IFN, and CRP were observed in the atorvastatin group. Also observed were significant day-wise changes in inflammatory biomarkers.	IL-1, IL-6, TNF-α, IFN-γ, and CRP	NCT02681653 [104]	Short duration of study with only 40 enrollments.
Simvastatin	Asthma	3	8 weeks	Inclusion Criteria: Patients aged 18–70 years old with mild-to-moderate persistent asthmatics, FEV1 ≥ 50% of predicted. Exclusion Criteria: Previous history of renal or liver disease, serum creatinine > 2 mg/dL, pregnancy or lactation, allergic to statins or have developed myositis, asthma exacerbation requiring oral corticosteroids in the past 3 months, treated with immunosuppressive agents, unwilling to cooperate with the study.	NRP	Sputum eosinophils, phosphorylated p38 MAPK	NCT01266434	Short study duration with an enrollment of only 44 participants.
Simvastatin	Heart Failure	1	3 years	Inclusion Criteria: Adults aged 18 to 85 years, symptomatic heart failure (NYHA class I to III), left ventricular ejection fraction < 0.40, and ability to give written informed consent. Exclusion Criteria: Pregnant or lactating women, heart failure due to specific conditions (e.g., active myocarditis, congenital heart disease), NYHA class IV symptoms, current or previous statin treatment, plasma LDL-C > 130 mg/dL with certain conditions (e.g., ischemic cardiomyopathy), progressive systemic diseases, uncorrected endocrine disorders, significant renal or hepatic disease, and inability or unwillingness to cooperate with the study.	NRP	Monocyte TF	NCT00769210	The study had a small sample size of only 12 enrollments.
Rosuvastatin	Sepsis + Acute Lung Injury	3	28 Days	Inclusion Criteria: Patients fulfilling the diagnostic parameters for systemic inflammatory response syndrome (SIRS)—operationalised by predefined thresholds for leukocyte count, core body temperature, and heart rate—concomitant with clinically suspected or microbiologically confirmed infection, and meeting the Berlin-derived definitional elements for acute lung injury (ALI), namely: (i) acute-onset hypoxaemia, (ii) radiographically confirmed bilateral pulmonary infiltrates, (iii) requirement for positive-pressure ventilatory support, and (iv) absence of haemodynamic evidence of left atrial hypertension. All ALI-defining parameters must manifest within a contiguous 24 h interval. Eligibility further requires enrolment within 48 h of ALI onset and ≤7 days from the initiation of invasive mechanical ventilation. Exclusion Criteria: Inability to secure informed consent; age < 18 years; duration of invasive mechanical ventilation exceeding 7 days; >48 h elapsed since fulfilment of ALI diagnostic criteria; designation for comfort measures only; inability to absorb enteral pharmacotherapy; prior statin exposure within the preceding washout interval; documented statin hypersensitivity or intolerance; attending physician–mandated statin therapy; elevated creatine kinase (CK), alanine aminotransferase (ALT), or aspartate aminotransferase (AST) beyond upper reference thresholds; untreated hypothyroidism; pregnancy or lactation; concurrent use of contraindicated pharmacologic agents; advanced chronic liver disease; moribund clinical status; chronic respiratory failure requiring long-term ventilatory support; home mechanical ventilation outside the context of obstructive sleep apnoea; diffuse alveolar hemorrhage; severe thermal injury; interstitial lung disease necessitating domiciliary oxygen therapy; unwillingness to adhere to the ARDS Network mechanical ventilation protocol; severe structural or ischaemic cardiac pathology; recent myocardial infarction; recent intracranial haemorrhage; or hyperpyrexia exceeding 40.3 °C.	No results pertaining to changes in inflammatory markers.	CRP	NCT00979121 [105,106,107,108]	The study was terminated and also had a short duration
Rosuvastatin vs. Atorvastatin	Hyperlipidemia	2 + 3	8 weeks	Inclusion Criteria: Adults aged 20–75 years, of either sex, with fasting low-density lipoprotein cholesterol (LDL-C) concentrations between 160 and 190 mg/dL and fasting triglyceride concentrations between 200 and 499 mg/dL at screening. Exclusion Criteria: Age < 20 years or >75 years; current use of lipid-lowering pharmacotherapy; concomitant intake of omega-3 fatty acid preparations or garlic-derived nutraceuticals; documented hypersensitivity to statin-class agents; concurrent use of systemic anti-inflammatory drugs (including corticosteroids or nonsteroidal anti-inflammatory drugs); intake of antioxidant vitamins A, C, or E; evidence of renal or hepatic impairment; pregnancy or lactation; active serious infection; or presence of a terminal illness.	NRP	Hs-CRP	NCT02979704	Small sample size with only 90 enrollments.
Simvastatin	Sickle Cell Disease	1 + 2	3 months	Inclusion Criteria: Patients aged ≥ 10 years with a confirmed diagnosis of sickle cell disease (HbSS genotype or S/β^0^-thalassemia), who have experienced ≥ 3 vaso-occlusive pain episodes within the 12 months preceding enrolment, and with a body mass ≥ 30 kg. Exclusion Criteria: Creatine kinase (CK) concentration exceeding the upper normal limit (UNL); total cholesterol < 90 mg/dL or triglycerides < 30 mg/dL; renal dysfunction defined as serum creatinine > 1.5 × UNL; hepatic dysfunction defined as alanine aminotransferase (ALT) > 2 × UNL; receipt of any pharmacologic agents with known metabolic interactions with statins within the preceding 30 days; hospitalization for vaso-occlusive pain within the preceding 30 days; receipt of red blood cell transfusion within the preceding 30 days; current pregnancy or lactation; musculoskeletal disorders associated with elevated CK; history of substance misuse; chronic pain of non–sickle cell etiology; or significant cognitive or neurological impairment precluding the use of smartphone technology or completion of electronic pain diaries.	Significant decrease in plasma high sensitivity C-reactive protein level after treatment with simvastatin	Hs-CRP	NCT01702246 [109]	Small sample size with only 24 enrollment.
Atorvastatin	Asthma + COPD + Smoking	2	8 weeks	Inclusion Criteria: Adults aged 18–60 years with a confirmed clinical diagnosis of asthma of >1 year’s duration, currently symptomatic, and with a cumulative smoking exposure exceeding 5 pack-years. Eligible participants should be receiving only short-acting β_2_-adrenergic agonists as bronchodilator therapy; other asthma-related pharmacotherapies may be tapered or discontinued if the patient demonstrates clinical stability. Exclusion Criteria: Never-smokers or former smokers; current statin therapy; unstable or poorly controlled asthma; documented hypersensitivity or intolerance to statins; history of statin-associated myopathy or myositis; or concurrent use of pharmacological agents with known clinically significant interactions with statins.	At 4 weeks, there was no improvement in the atorvastatin group compared to the placebo group in morning peak expiratory flow [−10.67 L/min, 95% CI −38.70 to 17.37, *p* = 0.449], but there was an improvement with atorvastatin in asthma quality of life score [0.52, 95% CI 0.17 to 0.87 *p* = 0.005]. There was no significant improvement with atorvastatin and inhaled beclometasone compared to inhaled beclometasone alone in outcome measures at 8 weeks.	LB4, MPO, IL-6, IL-8, IL-10, hsCRP, sputum inflammatory cells	NCT00463827 [110]	Short duration of study with sample size of 71.
Atorvastatin	Bronchiectasis + Pseudomonas aeruginosa	4	7.5 months	Inclusion Criteria: Patients aged 18–80 years with an established radiological diagnosis of bronchiectasis (CT of the chest) and colonized with Pseudomonas Aeruginosa. Patients must be able to give informed consent. Exclusion Criteria: Current smokers or ex-smokers of less than 1 year with a smoking history of more than 15 pack years, cystic fibrosis, active allergic bronchopulmonary aspergillosis, active tuberculosis, poorly controlled asthma, pregnancy or breastfeeding, known allergy to statins, active malignancy, chronic liver disease, established cardiovascular or cerebrovascular disease, or statin use in the last year.	Twenty-seven patients completed the study. Atorvastatin did not significantly improve the primary end point of cough as measured by the Leicester Cough Questionnaire (mean difference, 1.92; 95% CI for difference, −0.57–4.41; *p* = 0.12). However, atorvastatin treatment resulted in an improved St. Georges Respiratory Questionnaire (−5.62 points; *p* = 0.016) and reduced serum levels of CXCL8 (*p* = 0.04), tumour necrosis factor (*p* = 0.01), and intercellular adhesion molecule 1 (*p* = 0.04). There was a trend toward improvement in serum C-reactive protein and serum neutrophil counts (*p* = 0.07 and *p* = 0.06, respectively). We demonstrated in vitro that atorvastatin 10 μM reduced formyl-methionyl-leucyl phenylalanine-induced upregulation of CD11b expression and changes in calcium flux, reflecting an ability to decrease neutrophil activation.	Sputum neutrophil numbers and apoptosis; neutrophil activation in the airway measured by sputum myeloperoxidase, free elastase activity, and CXCL8; systemic inflammation measured by WBC count, CRP, and erythrocyte sedimentation rate; other markers of systemic inflammation, including concentrations of IL-1b, IL-6, CXCL8, IL-10, IL-12p70, TNF-α, and ICAM-1	NCT01299194 [111]	Small sample size of only 32 participants.
Atorvastatin	Complications of Renal Dialysis	Withdrawn	24 weeks	Inclusion Criteria: Adults aged 18–110 years, clinically stable on recombinant human erythropoietin (Epogen^®^) with maintained hemoglobin concentrations between 9.5 and 11.5 g/dL for ≥3 consecutive weeks prior to enrolment, no statin exposure within the preceding 3 months, and demonstrable adherence to prescribed haemodialysis regimens and concomitant medications. Exclusion Criteria: Documented history of myocardial infarction, cerebrovascular accident, or clinically significant peripheral or coronary vascular disease; hospitalization within 15 days prior to screening; major haemorrhagic events within the preceding 15 days (including traumatic, gastrointestinal, genitourinary, or menorrhagic bleeding); clinically evident hepatic dysfunction; active malignant neoplasm; or known haematologic pathology.	No Results Posted	Ferritin, CRP	NCT02764736	Study was withdrawn as the enrollment did not progress as anticipated by the researchers.
Simvastatin	Perioperative Inflammatory Response	4	72 h	Inclusion Criteria: Patients aged 18–80 years scheduled for elective major spine surgery (multilevel (2–6 level) open thoracic or lumbar spine surgery with instrumentation). Exclusion Criteria: Pregnancy; lactating females; oral or parenteral corticosteroid use in the past 30 days; elevation of AST or ALT > 3× normal; elevation of creatinine kinase > 2× normal; previous adverse drug reaction to any medication in the statin class; current use of fibrates, niacin, itraconazole, ketoconazole, macrolide antibiotics, HIV protease inhibitors, and/or nefazodone; active liver disease; current statin use; use of certain anti-inflammatory medications within the last 30 days and post-operative period.	Results were reported without significance level.	CRP, IL-6, TNF-α	NCT00656292	Study duration is short with a sample size of only 61 participants.
Atorvastatin	Metabolic Syndrome	1 + 2	6 weeks	Inclusion Criteria: Female participants aged 18–75 years meeting diagnostic criteria for metabolic syndrome, operationalised as central adiposity (abdominal circumference > 35 inches), hypertriglyceridemia (>150 mg/dL), reduced high-density lipoprotein cholesterol (HDL-C < 50 mg/dL), elevated blood pressure (>130/85 mmHg), and impaired fasting glycaemia (fasting plasma glucose > 100 mg/dL). Exclusion Criteria: Current pregnancy or intent to conceive within the ensuing 6–12 months; ongoing therapy with lipid-lowering agents; presence of obstructive hepatobiliary pathology or advanced hepatic insufficiency; established diabetes mellitus, clinically manifest cardiovascular disease, hypothyroidism, active infectious processes, or malignancy; major surgical intervention within the preceding months; eligibility for statin therapy based on low-density lipoprotein cholesterol thresholds, composite cardiovascular risk factors, or Framingham risk score; documented hypersensitivity or idiosyncratic reaction to statins; and unexplained creatine kinase elevation > 3 × the upper limit of normal.	At 6 weeks post-randomisation, the atorvastatin arm demonstrated pronounced and statistically significant decrements in total cholesterol, LDL-C, triglycerides, ApoB, and the ApoB/ApoA1 ratio compared with placebo, with these lipid-lowering effects persisting unabated through the 12-week follow-up. Glycaemic indices (fasting plasma glucose), HDL-C, hs-CRP, serum leptin, ApoA1, ICAM-1, and platelet activity metrics exhibited no significant between-group divergence. Intriguingly, VCAM-1 concentrations increased significantly within the atorvastatin cohort at both interim and terminal assessments, representing a paradoxical pro-adhesive signal in the context of otherwise favourable lipid modulation. Anthropometric indices (waist circumference) and haemodynamic parameters (systolic and diastolic blood pressure) remained stable across intervention arms, suggesting the observed biochemical shifts were independent of changes in adiposity or vascular tone.	Hs-CRP, sICAM, sVCAM, PAI-1, MPO	NCT01785615 [112]	Study was performed for a short duration.
Simvastatin	Septic Shock	2 + 3	24 h	Inclusion Criteria: Adults aged > 18 years presenting with hypotension necessitating vasopressor support and clinical suspicion of infection. Exclusion Criteria: Pregnancy; hepatic failure defined by alanine aminotransferase (ALT) or aspartate aminotransferase (AST) concentrations exceeding 120 U/L; rhabdomyolysis with creatine phosphokinase (CPK) > 3× the upper limit of normal; designation for comfort measures only; chronic liver disease including cirrhosis; concurrent administration of cyclosporine, digoxin, or any statin therapy; and inability to receive enteral medications via oral or nasogastric tube routes.	In this cohort, 28 plasma specimens derived from 14 individuals meeting enrolment criteria underwent quantitative biochemical profiling. Circulating coenzyme Q10 (CoQ10) concentrations were profoundly diminished relative to physiologically normative values, with a median level of 0.49 μmol/L (interquartile range [IQR], 0.26–0.62), in stark contrast to healthy reference participants (0.95 ± 0.29 μmol/L; *p* < 0.0001). Temporal trajectory analysis demonstrated no statistically significant modulatory effect of statin administration on systemic CoQ10 abundance over the observation interval (*p* = 0.13). Bivariate correlation modelling revealed that CoQ10 concentrations exhibited statistically robust associations with a spectrum of endothelial activation and pro-/anti-inflammatory mediators, including vascular cell adhesion molecule-1 (VCAM-1; *r*^2^ = 0.20, *p* = 0.008), tumour necrosis factor-α (TNF-α; *r*^2^ = 0.28, *p* = 0.004), interleukin-8 (IL-8; *r*^2^ = 0.21, *p* = 0.015), interleukin-10 (IL-10; *r*^2^ = 0.18, *p* = 0.025), E-selectin (*r*^2^ = 0.17, *p* = 0.03), interleukin-1 receptor antagonist (IL-1ra; *r*^2^ = 0.21, *p* = 0.014), interleukin-6 (IL-6; *r*^2^ = 0.17, *p* = 0.029), and interleukin-2 (IL-2; *r*^2^ = 0.23, *p* = 0.009). Following multivariable adjustment for low-density lipoprotein cholesterol, inverse associations of biological and statistical significance persisted for VCAM-1 (*r*^2^ = 0.24, *p* = 0.01) and IL-10 (*r*^2^ = 0.24, *p* = 0.02), thereby implicating CoQ10 insufficiency in the modulation of both vascular adhesion molecule expression and counter-regulatory anti-inflammatory signalling in this patient population.	VCAM, TNF-α, IL-8, IL-10, E-selectin, IL-1ra, IL-6, IL-2	NCT00676897 [113]	Small sample size of only 18 participants out of the expected 60.
Atorvastatin	Hip Fracture + Myocardial Ischemia + Inflammation	2	2 days	Inclusion Criteria: Patients aged ≥ 65 years with a hip fracture or scheduled for elective hip or knee arthroplasty, and with an anticipated life expectancy > 3 months. Exclusion Criteria: Pathological hip fracture secondary to cancer; current or recent statin use; prior statin intolerance; acute myocardial infarction or unstable angina; history of myocardial infarction, acute coronary syndrome, angina, coronary or arterial revascularisation, peripheral arterial disease, stroke, or transient ischaemic attack; muscle disorders; significant hepatic disease or alanine aminotransferase (ALT) > 3× the upper limit of normal; severe renal impairment (creatinine clearance < 30 mL/min); treatment with HIV or Hepatitis C protease inhibitors, erythromycin, clarithromycin, niacin, or azole antifungals; and pregnancy, planned pregnancy, or breastfeeding.	From an initial screening cohort of 556 subjects, 22 individuals met the stringent eligibility criteria and were subsequently enrolled—comprising 4 cases of fragility hip fracture, 11 candidates for elective total hip arthroplasty, and 7 for total knee arthroplasty—of whom 2 participants later withdrew consent. Baseline preoperative assessment revealed that 80% of the study population exhibited quantifiable circulating concentrations of high-sensitivity cardiac troponin I (hs-cTnI) exceeding 1.1 pg/mL, indicative of subclinical myocardial injury. A perioperative increment in hs-cTnI of ≥10 pg/mL, fulfilling criteria for clinically relevant biomarker elevation, was documented in 20% of participants, with no evidence of pharmacological attenuation by perioperative atorvastatin exposure. Biomarker profiling demonstrated a postoperative surge in high-sensitivity C-reactive protein (hs-CRP) in 95% (19/20) of evaluable subjects, paralleled by a universal escalation in plasma interleukin-6 (IL-6) concentrations; neither inflammatory axis was modulated by statin therapy. On postoperative day two, IL-6 concentrations exhibited a statistically significant positive monotonic association with hs-cTnI levels (Spearman’s ρ = 0.59, *p* = 0.02), suggesting potential mechanistic interplay between systemic inflammatory activation and myocardial injury. Participant accrual was substantially constrained by the high baseline prevalence of antecedent statin therapy within the screened surgical population and by the disproportionately elevated rate of exclusion criteria fulfilment among patients presenting with hip fractures.	hs-CRP, IL-1β, IL-2, IL-2r, IL-4, IL-5, IL-6, IL-8, IL-10, IL-12, IL-13, IL-17, TNFα, IFNγ, sCD-40L	NCT02197065 [114]	Short duration of study and a small size of only 20 participants of the estimated 30.
Atorvastatin	Vascular Disease	NA	10 Months	Inclusion Criteria: Adults aged ≥ 21 years, not currently receiving statins or other lipid-lowering agents, with no prior history of statin use, and presenting with at least two of the following cardiovascular risk factors: Age (men ≥ 45 years, women ≥ 55 years) Hypertension Cigarette smoking Coronary heart disease (CHD) equivalents Prior stroke or transient ischemic attack Family history of premature CHD Total cholesterol ≥ 240 mg/dL and/or LDL cholesterol ≥ 160 mg/dL Low HDL cholesterol (<40 mg/dL) Exclusion Criteria: Current or prior statin use; immediate clinical indication for statin therapy; use of investigational drugs; chronic or active hepatic or muscular disease; alcohol abuse; prior myocardial infarction; heart failure; uncontrolled arrhythmias; clinically significant hematological or biochemical abnormalities; history of cancer within the recent period; pregnancy or breastfeeding; or concomitant use of medications known to interact with statins (e.g., CYP3A4 inhibitors, nicotinic acid, gemfibrozil).	No Results Posted	Gene expression of inflammatory markers	NCT00293748	N/A
Simvastatin	Heart Disease	NA	12 months	Inclusion Criteria: Adults (>18 years) with documented atherosclerosis in at least one vascular territory, defined as: Moderate aortic atherosclerosis (>3.9 mm) on transesophageal echocardiography, Moderate coronary artery disease (>50% stenosis in at least one coronary artery) on cardiac catheterization, 50% carotid stenosis on ultrasound, or Clinically documented peripheral vascular disease. Exclusion Criteria: Current statin therapy equivalent to or exceeding 80 mg simvastatin; presence of pacemaker, automated implantable cardioverter-defibrillator (AICD), or aneurysm clips; abnormal nasopharyngeal anatomy; active peptic ulcer disease; severe dysphagia; baseline liver transaminases or serum creatinine > 2× the upper limit of normal; decompensated congestive heart failure; or inability to provide informed consent.	Significant differences were observed between the low-dose and high-dose statin therapy in reduction in low-density lipoprotein cholesterol (LDL-c) (10 mg/dL, *p* = 0.001), total cholesterol (16.2 mg/dL, *p* < 0.001), vessel wall area (19.0 mm^2^, *p* < 0.001) and volume (343.4 mm^3^, *p* < 0.001), as well as increase in lumen area (54.4 mm^2^, *p* < 0.001) and volume (1038 mm^3^, *p* < 0.001). LDL-c lowering was significantly associated with aortic wall area and volume reduction in both groups.	CRP, IL-6, TNF-α	NCT00125060 [115]	Small sample size of 72 participants.
Simvastatin	Severe Asthma	2	12 weeks	Inclusion Criteria: Statin-naïve adults aged > 18 years with severe asthma as defined by ATS; receiving inhaled corticosteroids (ICS) and long-acting β_2_-agonists (LABA); confirmed allergic asthma (serum IgE > 100 kU/L, positive RAST panel, and/or peripheral blood absolute eosinophil count ≥ 700/mm^3^); and clinically stable for at least 4 weeks. Exclusion Criteria: Baseline FEV_1_ < 30% predicted; current smokers or former smokers with >5 pack-years smoking history; pregnancy; lactation; women of childbearing potential actively attempting to conceive; nasal or sinus surgery or trauma within the past 3 months; ischemic heart disease; liver disease; and concurrent use of medications such as amiodarone, verapamil, diltiazem, gemfibrozil, cyclosporine, antifungal azoles, or danazol.	No Results Posted	Th2 gene expression, IL-13, eotaxin-1, eotaxin-2, eotaxin-3, STAT 6, NO	NCT02433535	The study was performed for a short duration before it was withdrawn.
Simvastatin	Cystic Fibrosis	NA	15 days	Inclusion Criteria: Adults aged 18–50 years, in good general health as determined by medical history; able to comprehend and sign the informed consent form; able to comply with study requirements; and willing to use an acceptable method of birth control. Exclusion Criteria: History of diabetes requiring insulin therapy; use of NSAIDs or corticosteroids (except for nasal steroid preparations); current use of statin medications; active gingival disease; presence of any chronic inflammatory condition compromising immune function; and pregnancy or intention to become pregnant.	Polymorphonuclear leukocytes (PMN) count decreased with simvastatin	Oral mucosal PMNs	NCT00531882	Short study duration and has a small sample size of only 25 participants.
Atorvastatin	HIV + Atherosclerosis	NA	1 year	Inclusion Criteria: Men and women aged 18–60 years with a prior diagnosis of HIV infection; evidence of subclinical coronary artery disease (plaque on coronary CTA in the absence of cardiac symptoms or events, and TBR > 1.6 on PET imaging); on stable antiretroviral therapy for >6 months without regimen changes; and LDL-cholesterol between 70 and 130 mg/dL. Exclusion Criteria: History of acute coronary syndrome; contraindications to statin therapy; current statin use; serum AST or ALT > 2× upper limit of normal; renal disease or serum creatinine > 1.5 mg/dL; infectious illness within the preceding 3 months; contraindications to beta-blocker or nitroglycerin use; body weight > 300 lbs; prior allergic reactions to iodine-containing contrast agents; significant radiation exposure within the past year; planned procedures involving substantial radiation exposure; pregnancy or breastfeeding; and coronary artery luminal narrowing > 70% on coronary CTA.	The study was conducted from 13 November 2009 to 13 January 2014. A total of 19 patients were assigned to atorvastatin and 21 to placebo. 37 (93%) of 40 participants completed the study, with equivalent discontinuation rates in both groups. Baseline characteristics were similar between groups. After 12 months, change in FDG-PET uptake of the most diseased segment of the aorta was not different between atorvastatin and placebo, but technically adequate results comparing longitudinal changes in identical regions could be assessed in only 21 patients (atorvastatin Δ −0.03, 95% CI −0.17 to 0.12, vs. placebo Δ −0.06, −0.25 to 0.13; *p* = 0.77). Change in plaque could be assessed in all 37 people completing the study. Atorvastatin reduced non-calcified coronary plaque volume relative to placebo: median change −19.4% (IQR −39.2 to 9.3) versus 20.4% (−7.1 to 94.4; *p* = 0.009, n = 37). The number of high-risk plaques was significantly reduced in the atorvastatin group compared with the placebo group: change in number of low attenuation plaques −0.2 (95% CI −0.6 to 0.2) versus 0.4 (0.0, 0.7; *p* = 0.03; n = 37); and change in number of positively remodelled plaques −0.2 (−0.4 to 0.1) versus 0.4 (−0.1 to 0.8; *p* = 0.04; n = 37). Direct LDL-cholesterol (−1.00 mmol/L, 95% CI −1.38 to 0.61 vs. 0.30 mmol/L, 0.04 to 0.55, *p* < 0.0001) and lipoprotein-associated phospholipase A2 (−52.2 ng/mL, 95% CI −70.4 to −34.0, vs. −13.3 ng/mL, −32.8 to 6.2; *p* = 0.005; n = 37) decreased significantly with atorvastatin relative to placebo. Statin therapy was well tolerated, with a low incidence of clinical adverse events.	Coronary and Aortic Plaque Inflammation, CRP, IL-6	NCT00965185 [116,117]	Small sample size of 40 participants.
Atorvastatin	Myocardial Infarction + Inflammation + Acute Coronary Syndrome + Reperfusion injury	1	8 h	Inclusion Criteria: Males aged 18–40 years. Exclusion Criteria: Females, unwillingness to participate, inability to communicate or understand study instructions, participation in another ongoing trial, any chronic or acute disease within 30 days of inclusion, any regular or temporary medication within 15 days of inclusion, and current smokers.	NRP	IL-6, CRP, TNFR1, TNFR2, Fas, Fas ligand, MMP-2, TIMP-2, sP-selectin, PF-4, β-thromboglobulin	NCT02286544	The study is mainly for testing for salmonella vaccine. Study performed for a short duration and sample size of only 36 participants.
Rosuvastatin	Coronary Artery Disease	2	12 weeks	Inclusion Criteria: Adults > 18 years, fluent in English or Spanish, willing to participate, and clinically stable. Scheduled for cardiac catheterization and PCI with intent to stent; willing to receive high-dose cholesterol-lowering therapy for the study duration; able to provide signed informed consent. Women of childbearing potential must agree to use acceptable contraception. Presence of a proposed non-culprit YELLOW study lesion with maximum 4 mm LCBI ≥ 150. Exclusion Criteria: Acute myocardial infarction (ST-segment elevation, new Q waves, or non-ST-segment elevation with CK-MB > 5× upper normal limit within 72 h); cardiogenic shock; requirement for coronary artery bypass graft surgery; platelet count < 100,000 cells/mm^3^; comorbidities limiting life expectancy to ≤ 1 year; concurrent enrolment in another investigational study; liver disease; serum creatinine > 2.0 mg/dL; pregnancy or planned pregnancy during the trial; heart transplant recipients or candidates; active autoimmune disease; and breastfeeding women.	Baseline OCT minimal fibrous cap thickness (FCT) was 100.9 ± 41.7 μm, which increased to 108.6 ± 39.6 μm at follow-up, and baseline CEC was 0.81 ± 0.14, which increased at follow-up to 0.84 ± 0.14 (*p* = 0.003). Thin-cap fibroatheroma prevalence decreased from 20.0% to 7.1% (*p* = 0.003). Changes in FCT were independently associated with CEC increase by multivariate analysis (β: 0.30; *p* = 0.01). PBMC microarray analysis detected 117 genes that were differentially expressed at follow-up compared to baseline, including genes playing key roles in cholesterol synthesis (SQLE), regulation of fatty acids unsaturation (FADS1), cellular cholesterol uptake (LDLR), efflux (ABCA1 and ABCG1), and inflammation (DHCR24). Weighted coexpression network analysis revealed unique clusters of genes associated with favourable FCT and CEC changes.	hsCRP	NCT01837823 [118]	Study was performed for a short duration.
Simvastatin	Dyslipidemia	NA	8 weeks	Inclusion Criteria: Patients aged 18 years or older, LDL cholesterol concentration between 160 and 190 mg/dL in patients with less than 2 cardiovascular risk factors, and LDL concentration between 130 and 160 mg/dL in patients with 2 or more cardiovascular risk factors (defined as age ≥ 45 years in men and ≥55 years in women, smoking habit, hypertension ≥ 140/90 mmHg, diabetes mellitus, HDL cholesterol ≤ 40 mg/dL, and family history of cardiovascular disease). Exclusion Criteria: Triglyceride concentration > 400 mg/dL, diabetes mellitus, kidney, liver, or thyroid disease.	Hs-CRP, ROS, ICAM-1 and E-selectin significantly decreased. IL-6 and TNF-α increased but *p* > 0.05. VCAM-1 decreased but *p* > 0.05	hsCRP, IL-6, TNF-α, ROS, VCAM-1, ICAM-1, E-selectin	NCT02304926	The study first assigned Simvastatin and Ezetimibe separately and after 4 weeks both groups were administered a combined therapy of both the drugs. Short study duration with sample size of 42 participants.
Atorvastatin	Myocardial Infarction (perioperative complications) + Myocardial Ischemia+ Inflammation	NA	48 h	Inclusion Criteria: Adults aged > 45 years undergoing elective high-risk surgery as defined by the POISE criteria. Exclusion Criteria: Absence of informed consent; contraindications to statin therapy (including liver insufficiency, cirrhosis, active muscular disorders, myopathy, or prior adverse reaction to statins); pregnancy; concurrent enrolment in another conflicting study; prior enrolment in STAR VaS; current statin use.	Fifty-six participants completed the 30-day follow-up. The mean (standard deviation) changes in CRP levels from baseline at 48 h in Groups AA, PA, and PP were 141.0 (72.4), 153.5 (42.2), and 111.2 (84.6), respectively. The mean differences (95% confidence interval) at 48 h for AA vs. PA, AA vs. PP, and PA vs. PP were: −20.1 (−81.2 to 41.1), 22.7 (−31.7 to 77.2), and 42.8 (−20.0 to 105.7), respectively, adjusting for baseline CRP, type of procedure, presence of coronary artery disease, use of medications, and for multiple comparisons using Tukey’s method.	CRP	NCT00967434 [119]	Short study duration with sample size of 60 participants.
Atorvastatin	Chronic Kidney Disease	3	12 weeks	Inclusion Criteria Patients with vitamin D–treated chronic kidney disease (CKD) undergoing haemodialysis for ≥3 months; presence of tunnelled permanent catheters for ≥6 months prior to enrolment; stable Kt/V > 45 litres; receiving atorvastatin therapy; no infectious or inflammatory episodes for ≥8 weeks; two consecutive measurements showing parathyroid hormone (PTH) < 400 pg/mL, serum calcium < 10.2 mg/dL, and serum phosphorus < 7.0 mg/dL. Exclusion Criteria Age < 18 years; pregnancy; hospitalization within the preceding 4 weeks; current use of immunosuppressive agents.	No Results Posted	IL-2, IL-4, IL-5, IL-6, IL-10, IL-13, TNF-β, CD3, CD4, CD8, CD19, CD25, CD56, CD69, CD95, COX-2, iNOS, PGE2, FGF23,	NCT01820767	Atorvastatin is used as a comparator. Study has a small sample size of 31 participants.
Rosuvastatin	HIV + Cardiovascular Disease	4	6 months	Inclusion Criteria Participants aged 40–90 years; documented HIV infection; on antiretroviral therapy (ART) for >1 year with undetectable viral load; CD4 count > 350 cells/μL; Framingham risk score between 10–20%. Exclusion Criteria Uncontrolled diabetes mellitus or hypertension; known coronary artery disease; chronic renal failure; total cholesterol > 5.8 mmol/L; LDL cholesterol > 4.0 mmol/L; current statin therapy for baseline dyslipidemia; pregnancy or lactation; untreated hepatitis B or C; inflammatory or autoimmune disorders; baseline regional perfusion abnormalities on stress myocardial contrast echocardiography (MCE).	NRP	Vascular inflammation (TBRmax)	NCT02234492	Small sample size of 35 participants from the original estimated 82.
Simvastatin	Alzheimer’s Disease	2	9 months	Inclusion Criteria: Ages 35–69 with a parent having Alzheimer’s disease. Exclusion Criteria: Current use of cholesterol-lowering medication, active liver disease, history of adverse reaction to statins, contraindication to lumbar puncture, elevated lab values, use of medications known to interact with statins, history of dementia, currently pregnant or planning to become pregnant, excessive grapefruit juice consumption, involvement in another investigational drug study	Reduction in hs-CRP but not significant	Hs-CRP	NCT00486044	The non-significant results may be due to the short duration of trial.
Atorvastatin	HIV Dementia	4	12 weeks	Inclusion Criteria: Living with HIV-1 infection, were on stable HIV treatment with controlled viral load, sufficient CD4 T-cell count, willing to comply with trial, elevated hs-CRP levels. Exclusion Criteria: Use of other lipid-lowering drugs, drugs that interact with the statin, pregnant, beast-feeding, active drug abuse, severe illness, allergic to statins, abnormal blood count.	No significant change in inflammatory markers	MCP-1, sCD14, sCD163, CD16	NCT01263938	The non-significant results may be due to small sample size (5 participants).
Atorvastatin	Type 1 Diabetes Mellitus + Hypercholesterolemia	3	6 months	Inclusion Criteria Project 1: Type 1 diabetes mellitus (T1DM) diagnosed for >1 year; any HbA1C level; stable insulin therapy; ages 10–20 years; both sexes; BMI < 85th percentile; fasting LDL-C > 100 mg/dL; normal thyroid function. Projects 2 and 3: T1DM diagnosed for >3 years; HbA1C > 8%; stable insulin therapy; ages 12–20 years; both sexes; BMI < 85th percentile; fasting LDL-C > 100 mg/dL; normal thyroid function. Exclusion Criteria *(applies to Projects 1, 2, and 3)* Severe dyslipidemia (LDL-C > 160 mg/dL, triglycerides > 400 mg/dL); smoking; pregnancy; current use of anti-inflammatory, immunomodulatory, lipid-lowering, or additional antidiabetic medications; hypertension and/or microalbuminuria (permitted only with balanced randomisation and standardized treatment).	No significant change in hsCRP	hsCRP	NCT01236365	The non-significant results maybe due to the short duration of trial.
Rosuvastatin	Acute Coronary Syndrome	4	6 months	Inclusion Criteria: Adults aged ≥ 19 years with acute coronary syndrome or carotid artery disease (20–50% stenosis) and at least one ^18F^-FDG–avid lesion (target-to-background ratio [TBR] ≥ 1.6) in the carotid artery confirmed by PET/CT imaging; provision of written informed consent. Exclusion Criteria: History of carotid endarterectomy or stenting; planned major surgery within the next 6 months; use of statin or ezetimibe therapy within the previous 4 weeks; chronic illness requiring steroid therapy; end-stage renal disease; chronic liver disease; malignancy within the past 3 years; pregnancy or breastfeeding; or life expectancy < 2 years.	NRP	hsCRP	NCT04056169	High dose rosuvastatin was compared with a group that was given low rosuvastatin and ezetimibe.
Simvastatin/Atorvastatin/Rosuvastatin	Coronary Heart Disease + Dyslipidemia	NA	36 Months	Inclusion Criteria: Male or female patients aged ≥ 18 years with high cardiovascular risk and otherwise good overall health. Exclusion Criteria: History of liver, kidney, pancreatic, or gallbladder disease; acute coronary syndrome within one month prior to enrolment; pregnancy; presence of inflammatory disease; or current treatment with medications known to affect triglyceride metabolism or concentration.	NRP	hsCRP	NCT02163044 [120]	N/A
Simvastatin	Metabolic Syndrome	6 weeks	4	Participants enrolled in the study were men and women aged 21 years or older who had a confirmed diagnosis of metabolic syndrome. This diagnosis was established based on the presence of at least three out of five clinical features: abdominal obesity, triglyceride levels greater than 150 mg/dL, HDL cholesterol below 40 mg/dL for men or below 50 mg/dL for women, blood pressure exceeding 130/85 mm Hg, and fasting glucose levels above 100 mg/dL. Subjects were excluded if they had a history of bleeding disorders, drug or alcohol abuse, a prothrombin time more than 1.5 times the control value, a platelet count below 100,000/mm^3^, a hematocrit level under 25%, or a creatinine level above 4.0 mg/dL. Additional exclusion criteria included recent or planned surgery or angioplasty, any history of gastrointestinal or other bleeding events, drug-induced medical conditions, recent trauma, active malignancy, rheumatic or autoimmune diseases, coronary artery disease, prior stroke, participation in another drug trial within the preceding month, treatment with intravenous platelet glycoprotein IIb/IIIa inhibitors or thienopyridines within the past six months, and use of statins or aspirin within the previous four weeks.	Results posted but not analyzed	CRP, IL-6	NCT00819403	Short duration of study with a small sample size (15 participants).
Rosuvastatin	Preeclampsia	2	NA	Inclusion Criteria: Women aged 20–35 years with a singleton, non-anomalous pregnancy; normal lipid profile; normal liver transaminase levels; white blood cell count between 4–11 × 10^3^/mm^3^; and C-reactive protein (CRP) < 3 mg/L. Exclusion Criteria: Refusal to participate; history of cardiac, respiratory, renal, neurological, or endocrine disorders; contraindications to statin therapy; concurrent treatment with fibrates, niacin, cyclosporine, clarithromycin, or erythromycin; inability to tolerate oral medications due to severe nausea or vomiting; multifetal gestation or intrauterine fetal demise; presence of fetal abnormalities; or requirement for emergency surgery.	NRP (Unknown Status)	CRP, IL-6	NCT04303806	N/A
Atorvastatin	Intracranial Aneurysm	2	6 months	Inclusion Criteria: Adults aged 18 or over, male or non-pregnant female, with a saccular UIA ≥ 3 mm identified on imaging and wall enhancement of the aneurysm by MRI VWI, and able to understand the trial and sign informed consent. Exclusion Criteria: Patients with MRI contraindications, planned aneurysm treatment within 6 months, taking anti-inflammatory drugs, dyslipidemia, severely impaired liver or renal functions, recurrent aneurysm retreatment, pregnant or lactating, with malignant diseases or poor compliance.	NRP	CRP, TNF-α, IL-1β, IL-6	NCT04149483	N/A
Atorvastatin	Ageing-related Inflammation in HIV	4	72 Weeks	Inclusion Criteria: Adults aged 45 years diagnosed with HIV-1 infection, receiving a specified antiretroviral therapy (ART) regimen for a minimum of 3 months, with documented undetectable plasma HIV-1 RNA levels for at least 12 consecutive months, and willing to provide voluntary written informed consent. Exclusion Criteria: History of virological failure to integrase inhibitor–based regimens; documented resistance mutations to integrase or nucleoside reverse transcriptase inhibitors (NRTIs); presence of systemic comorbid conditions (including hepatitis B or C co-infection, acute infections, or malignancy); current treatment with anti-inflammatory, anticoagulant, or antiplatelet agents; or use of statin therapy within the preceding 6 months.	Plasma inflammatory markers remained unchanged. Furthermore, no major change in T cells. However, there was a small decrease in number of CD38+ CD8 T cells and a small rise in the number of CD28-CD57+ T cells	IL-6, CRP	NCT02577042 [121,122]	One group had Raltegravir + atorvastatin.
Atorvastatin	HIV-1 Dementia	4	12 Weeks	Inclusion Criteria: Adults aged 18 or older with chronic HIV-1 on stable HAART, HIV viral load < 200 copies/mL for over 6 months, nadir CD4 count < 350 and current CD4 count > 100 cells/ul, hs-CRP > 2 mg/L, Karnofsky score ≥ 80, willing to use contraception, comply with study evaluations, and if female, undergo monthly pregnancy testing. Exclusion Criteria: Use of lipid-lowering or anti-inflammatory drugs, pregnancy or breastfeeding, active drug or alcohol abuse, allergy to Atorvastatin, history of myositis or rhabdomyolysis, recent serious illness or infection, elevated liver enzymes or renal insufficiency, certain blood count abnormalities, HCV infection, severe heart failure, active IV drug use, coronary artery disease, or allergy to Lidocaine for LP sub-study.	No significant difference in CD16, MCP1, CD163, CCR2, sCD14, CD38 and sCD163. Significant difference in hsCRP	CD16, MCP1, CD163, CCR2, sCD14, hsCRP, CD38, sCD163	NCT01600170	Further research needs to be carried out regarding why only hs-CRP results were significant.
Atorvastatin	Healthy	NA	7 days	Inclusion Criteria: Male or female, aged 18–50, fluent in English, BMI 18–30, willing and able to give informed consent, not on regular medications (except contraceptive pill). Exclusion Criteria: Regular medications (except contraceptive pill), significant psychiatric illness, alcohol or substance misuse disorder, significant hepatic or neurological conditions, hypersensitivity to atorvastatin, pregnant or breastfeeding, women not using contraception, recent participation in similar studies or studies involving medication.	NRP	hs-CRP	NCT03966859	Although the study was assessing emotional processing, they also assessed inflammatory markers.
Atorvastatin + Rosuvastatin	Type 2 Diabetes	4	6 months	Inclusion Criteria: Adults diagnosed with type II diabetes mellitus and documented hypercholesterolaemia. Exclusion Criteria: History or presence of hepatic impairment; renal insufficiency; coronary artery disease; other metabolic disorders; type I diabetes mellitus; autoimmune diseases; active or prior malignancy; current or recent infection; use of anti-inflammatory medications; major surgery within the preceding months; recent weight reduction or modification in antihypertensive therapy; prior or ongoing treatment with lipid-lowering agents; or any known contraindication to statin therapy.	NRP	Hs-CRP	NCT03784703	N/A
Simvastatin	Sickle Cell Disease	2 + 3	10 months	Inclusion Criteria: Children and adolescents aged 5 to 15 years presenting with a complete clinical manifestation of sickle cell disease, accompanied by an acute painful crisis. Exclusion Criteria: Presence of any other chronic medical condition; age less than 3 years or greater than 18 years; documented hepatic disease; renal impairment; or a diagnosis of diabetes mellitus.	NRP	hsCRP	NCT04301336	Study is comparing Simvastatin with other treatments.
Pravastatin	Postmenopausal osteoporosis	4	3 days	Inclusion Criteria: Chinese Han ethnic postmenopausal women with bone mineral density values less than 2.5 SD below the normal adult mean, willing to participate. Exclusion Criteria: Prior treatment with bisphosphonates, recent infections, evidence or history of cancer, contraindications to zoledronic acid or pravastatin, current use of certain medications (aminoglycosides, diuretics, thalidomide, fibrates, immunosuppressives, niacin), severe liver or renal insufficiency, and any condition precluding study participation.	NRP (Unknown Status)	CRP, IFN-γ, IL-6	NCT04719481	N/A
Atorvastatin, Rosuvastatin Simvastatin	Atherosclerosis	NA (Unknown Status)	8 weeks	Inclusion Criteria: Patients over 40 years old with high- or very-high-risk CVD and LDL-Cholesterol ≤ 4.0 mmol/L (high risk) or ≤3.5 mmol/L (very high risk). Must have a stable CV disease history, stable statin therapy, and patients not taking Ezetimibe. Exclusion Criteria: Uncontrolled hypertension, significant hypertriglyceridemia, pregnancy, hypersensitivity to study drugs, significant liver function test abnormalities, recent acute CV events, chronic inflammatory conditions, severe renal impairment, recent malignancy, substance abuse, recent use of systemic corticosteroids, inability to provide informed consent, or medications causing significant drug interactions with study medications.	NRP	hsCRP, IL-2, IL-6, ox-LDL	NCT03355027	Patients were given either Alirocumab and statin or Ezetimibe and statin
Atorvastatin	HIV-1	2	44 Weeks	Inclusion Criteria: HIV-1 infected individuals aged 18 or older on a stable ART regimen including a boosted PI for at least 6 months, with an undetectable HIV-1 RNA level (<40 copies/mL) and stable laboratory values within specified ranges. Participants must consent to contraception if of reproductive potential and agree to study requirements. Exclusion Criteria: Individuals with malignancy (excluding non-melanoma skin cancer), coronary artery disease, chronic hepatitis B or C, significant inflammatory conditions, pregnancy or breastfeeding, prior intolerance to statins, recent use of lipid-lowering or immunosuppressant therapies, heavy alcohol use, known coagulopathy, recent infections, or serious illness/trauma within 4 weeks prior to entry.	No significant change in IL-6. Other results were reported without significance level.	IL-6, MCP-1, CD40L, sCD14, sCD163,	NCT01351025	NA
Atorvastatin, Rosuvastatin	Heart Failure	4	6 months	Inclusion Criteria: Adults aged 18–65 with left ventricular ejection fraction < 40% and NYHA class II-III heart failure, optimized on standard CHF treatment for at least 1 month, and able to provide written informed consent. Exclusion Criteria: Hypersensitivity to statins, NYHA class IV, serum creatinine > 3 mg/dL, significant liver disease, current use of enzyme inducers/inhibitors, malignancy or chemotherapy, pregnancy or lactation, recent trial participation, uncontrolled diabetes or hypertension, or HIV/HBV/HCV infection.	NRP (Unknown Status)	hsCRP, IL-6	NCT05072054	NA
Rosuvastatin	Valvular Cardiac surgery	3	3 months	Inclusion Criteria: Adults undergoing single or multiple valve repairs/replacements, Bentall procedure (without other aortic procedures), with or without MAZE procedure for atrial fibrillation. Exclusion Criteria: Age under 18, urgent/emergency surgery, inability to consent, current or recent statin use, chronic anti-inflammatory drug use, hypersensitivity to rosuvastatin, active liver disease, pregnancy or nursing, drug interactions, severe renal impairment (creatinine clearance < 30 mL/min/1.73 m^2^), known myopathy or inflammatory disorders, HIV.	NRP	Inflammatory markers	NCT01425398	NA
Atorvastatin	Type 2 Diabetes, Hyperlipidemia, Hypertension	4	5 weeks	Inclusion Criteria: Adults aged 19–74 with type 2 diabetes, hyperlipidemia, stage I hypertension, and adequately controlled hemoglobin levels, who have not used medications for hyperlipidemia and hypertension in the past 3 months and have provided written consent. Exclusion Criteria: Patients contraindicated for Rovelito, pregnant/nursing women, significant blood pressure discrepancies, recent use of specific medications (e.g., ARBs, ACE inhibitors, statins), previous antidiabetic use, intolerance to study medications, genetic angioedema, past adverse reactions to statins, CPK > 5× ULN, secondary hypertension, poorly controlled hypothyroidism, severe cardiac issues within 6 months, renal or hepatic disease, pancreatitis, major gastrointestinal surgeries, chronic inflammatory conditions, autoimmune diseases, recent substance abuse, history of malignant tumours within 5 years, recent investigational product use, and other factors deemed by the investigator.	NRP	ICAM-1, IL-6, CRP,	NCT02842359	Study investigates combination with Ibersartan but compares with atorvastatin alone. Duration of the trial is short with only 11 participants actually enrolled from the original estimated 84 participants.
Simvastatin	Depression	1 + 2	7 Days	Inclusion Criteria: Adults aged 18–50, fluent in English, BMI between 18 and 30, willing and able to give informed consent, and not taking any regular medications except contraceptive pills. Exclusion Criteria: Current use of regular medications (except contraceptive pills), significant psychiatric illness, alcohol or substance misuse disorder, significant hepatic or neurological conditions, history of hemorrhagic stroke or lacunar infarct, known hyperglycemia/pre-diabetes, hypersensitivity to simvastatin or sucrose, pregnant or breastfeeding women, women of childbearing potential not using appropriate contraception, recent participation in studies using similar tasks or medications.	Seven-day simvastatin 20 mg treatment, compared to placebo, did not lead to any statistically significant changes on the majority of the emotional processing, reward learning, and verbal memory outcomes measured, nor it did for most of the questionnaires administered and hs-CRP levels.	CRP	NCT04652089 [123]	Short study duration with a sample size of 53 participants.
Rosuvastatin	Ulcerative Colitis	2 + 3	8 Weeks	Inclusion Criteria: Adults aged 18–70, Bact2 enterotyped one month prior to study, willingness to participate and sign informed consent (in Dutch), access to a −20 °C freezer. UC patients must be in remission or have mild to moderate active ulcerative colitis (Mayo score 4–10) with stable medication (8 weeks) and a Mayo endoscopic sub-score of 2–3 at week 0. Healthy Bact2 participants must have no physician-diagnosed diseases or disorders. Exclusion Criteria: Prior or ongoing use of statins, history of gastrointestinal surgery (except appendectomy), active liver disease, lactose intolerance, pre-diabetes, hereditary muscular disorders, alcohol abuse. UC patients: conditions causing profound immunosuppression, diagnosis of Crohn’s disease or indeterminate colitis, hypothyroidism, diabetes mellitus, severe renal impairment (creatinine clearance < 30 mL/min), myopathy, recent antibiotic use (past four months), steroid dependency requiring > 16 mg Medrol two weeks before week 0. Healthy Bact2 participants: family history of autoimmune chronic inflammatory diseases.	Recruiting	Hs-CRP	NCT04883840	No results published as the trial is still recruiting participants.
Atorvastatin	Non-ST Segment Elevation Acute Coronary Syndrome	2	30 Days	Inclusion Criteria: Adults (≥18 years) with non-ST elevation acute coronary syndrome who are statin-naive. Exclusion Criteria: Prior statin or colchicine therapy, known allergy to colchicine or statins, current treatment with potent CYP3A4 or P-glycoprotein inhibitors, previous or planned immunosuppressive therapy, active malignancy, severe kidney disease (creatinine > 3 mg/dL or dialysis), severe liver disease (ALT and/or AST > double normal values with total bilirubin > double normal values or altered coagulation), severe heart failure (NYHA class ≥ 3 or cardiogenic shock), severe acute or chronic gastrointestinal disease, pregnancy or lactation, current COVID-19 or other infectious disease, refusal of consent.	Recruiting	hsCRP	NCT05250596	Study is investigating colchicine + atorvastatin.
Simvastatin	Chronic Obstructive Pulmonary Disease + Inflammation	1	9 Weeks	Inclusion Criteria: Medically optimized COPD patients aged 40–79 years with serum CRP levels > 3 mg/L. Exclusion Criteria: Current smoker, COPD exacerbation in the last 2 months, active hepatic or severe renal dysfunction, connective tissue disease, chronic inflammatory disease, malignancy, acute illness, leukocytosis (>10,000 white blood cells), thrombocytosis (>450,000 platelets), recent history of myocardial infarction or angina in the last 6 months, pregnancy.	NRP	CRP	NCT00655993	Small sample size of 30 participants from the original estimated 40.
Atorvastatin	Chronic Chagas Disease	2	12 Months	Inclusion Criteria: Adults aged 18–50 years, weighing more than 40 kg, with confirmed T. cruzi infection and positive qPCR, normal lab values (except GGT > 2 × ULN), negative serum pregnancy test for women of reproductive age using effective contraception, and ability to comply with follow-up tests and visits. Exclusion Criteria: Signs of digestive form or chronic cardiac Chagas Disease stage II or higher, acute or chronic health conditions (including acute infections, HIV, diabetes, liver/kidney disease, hypothyroidism), family history of muscle disorders, pre-existing heart disease unrelated to Chagas, contraindications or hypersensitivity to study drugs, prior treatment for Chagas or with statins, concomitant use of antimicrobial agents or CYP3A4 modifiers, history of alcohol/drug abuse, inability to take oral medication, familial short QT syndrome, abnormal lab values (specific parameters), pregnancy or breastfeeding, refusal to use contraception during treatment.	Recruiting	TNF-α, IFN-γ, IL-10, IL-1β, IL-4, IL-17A, ICAM-1, VCAM-1, E-Selectin.	NCT04984616	NA
Atorvastatin + Rosuvastatin	Acute Coronary Syndrome	4	4 Weeks	Inclusion Criteria: Patients aged 18 and older, diagnosed with acute coronary syndrome, not previously taking statins, and who provide written informed consent. Exclusion Criteria: Patients on lipid-lowering drugs, with statin hypersensitivity or contraindications, undergoing surgical management, planned for coronary revascularization, pregnant or lactating women, and those with infections causing elevated inflammatory markers.	NRP	Hs-CRP	NCT06053983	Short study duration.
Simvastatin	Atherosclerosis + Hypercholesterolemia + Inflammation	4	6 weeks	Inclusion Criteria: Adults aged 18 to 75 years with obesity, defined as a body mass index (BMI) of ≥30 kg/m^2^, and low-density lipoprotein (LDL) cholesterol levels of ≥100 mg/dL. Eligible participants must not be taking vitamin or antioxidant supplements at the time of enrolment. Exclusion Criteria: Ongoing treatment with anti-hyperlipidaemic agents; serum triglyceride concentration > 500 mg/dL; history of myocardial infarction, percutaneous coronary intervention (angioplasty or stent placement), or coronary artery bypass graft surgery within the preceding six months; chronic administration of non-steroidal anti-inflammatory drugs (NSAIDs) or systemic corticosteroids; clinically significant hepatic disease or renal impairment; documented history of drug or alcohol misuse; concurrent participation in another clinical trial; receipt of an investigational medicinal product within 30 days prior to screening; active tobacco use; pregnancy; premenopausal women not employing reliable contraception; and anemia, defined as hemoglobin < 12 g/dL.	NRP	IL-1β, TNF-α, MMP-9	NCT04638400	The study was terminated due to lack of recruitment with the sample size being only 10.
Atorvastatin + Rosuvastatin	Clonal Cytopenia of Undetermined Significance, Myelodysplastic Syndromes	2	3 Months	Inclusion Criteria: Adults aged 18 and older diagnosed with CCUS (presence of somatic mutations with VAF ≥ 2% and unexplained persistent cytopenia in at least one lineage for at least 6 months) or lower-risk MDS (IPSS-R score ≤ 3.5 with at least one mutation with VAF ≥ 2%). Patients must be transfusion-independent and able to provide informed consent. Exclusion Criteria: CCUS patients with cytogenetic change alone, recent use of disease-modifying therapy (within the last 3 months), prior statin use within 1 year, history of other malignancies (unless treatment was completed > 2 years ago), use of investigational agents within 5 half-lives of the agent, allergic reactions to statins, uncontrolled intercurrent illness, pregnant or breastfeeding women, and patients with HIV or HCV on active treatment.	Recruiting	Hs-CTRP	NCT05483010	NA
Atorvastatin	Ulcerative Colitis	2	6 Months	Inclusion Criteria: Adults aged 18 and older, both male and female, with a negative pregnancy test and effective contraception. Exclusion Criteria: Breastfeeding, significant liver and kidney function abnormalities, colorectal cancer, severe UC, use of rectal or systemic steroids, immunosuppressives, or biological therapies.	NRP (Recruiting)	IL-6, MPO	NCT05561062	NA
Atorvastatin	Ulcerative Colitis	2	6 Months	Inclusion Criteria: Adults aged 18 to 75 years, both male and female, with a negative pregnancy test and effective contraception. Exclusion Criteria: Breastfeeding, significant liver and kidney function abnormalities, colorectal cancer, severe UC, use of rectal or systemic steroids, immunosuppressives, or biological therapies.	NRP (Recruiting)	IL-18	NCT05567068	NA
Atorvastatin	Breast Cancer	2	2 Years	Inclusion Criteria: Adults aged 18 years and older with a diagnosis of TNBC (including triple-negative inflammatory breast cancer), stage IIB or III disease, histological confirmation of breast carcinoma, residual disease post-neoadjuvant chemotherapy, ECOG performance status of 0–1, within 3 months from definitive surgery, and willing to take a statin for at least two years. Exclusion Criteria: Incomplete recovery from prior therapies, other active malignancies (except certain skin cancers or in situ cervical cancer), psychiatric or substance abuse disorders, recent statin therapy, use of other anti-lipidemic agents, hypersensitivity to statins, active liver disease, pregnancy or breastfeeding, distant metastasis, recent myocardial infarction, unstable cardiac conditions, and chronic steroid use.	NRP	ESR, CRP, IL-6	NCT03872388	Participants that were not eligible to receive atorvastatin will be enrolled into non-statin observation group with/without capecitabine treatment. Very small sample size of only 6 participants of the estimated 80.
Simvastatin	Obesity	NA	6 Weeks	Inclusion Criteria: Adults between 18 and 65 years of age with a body mass index (BMI) exceeding 30 kg/m^2^ and low-density lipoprotein (LDL) cholesterol levels greater than 100 mg/dL. Participants must not be receiving vitamin or antioxidant supplementation and must be willing to provide written informed consent. Exclusion Criteria: Current or recent use of lipid-lowering agents; serum triglyceride levels exceeding 500 mg/dL; history of myocardial infarction, percutaneous coronary intervention (angioplasty with or without stent placement), or coronary artery bypass grafting within the recent past; chronic administration of non-steroidal anti-inflammatory drugs (NSAIDs) or corticosteroids; clinically significant hepatic disease or renal impairment; history of drug or alcohol misuse; concurrent enrolment in another clinical trial; administration of any investigational product within the preceding 30 days; active tobacco smoking; pregnancy; premenopausal women not using reliable contraception; and anemia defined by hemoglobin concentration below 12 g/dL.	Results were reported without significance level	CD16, IL-1β	NCT01420328	N/A
Rosuvastatin	Coronary Artery Disease	4	1 Year	Inclusion Criteria: Males and females aged 40–75 years, capable and willing to provide informed consent, with high CAD PRS and subclinical atherosclerosis (plaque visible on CCTA causing < 70% luminal stenosis). Exclusion Criteria: History of cardiovascular disease, liver disease, significant liver enzyme abnormalities, eGFR < 60 mL/min/1.73 m^2^, significant allergic reactions to iodinated contrast, colchicine, or statins, current use of LDL cholesterol-lowering or anti-inflammatory medications, need for potent CY2P inhibitors, pregnancy or breastfeeding, BMI ≥ 40 kg/m^2^, inability to provide informed consent, or inability to hold breath for 10 s.	NRP (Recruiting)	CRP, IL-1β, IL-6	NCT05850091	N/A
Pitavastatin	HIV + Cardiovascular Disease	3	96 Months	Inclusion Criteria: HIV-1 infected individuals on combination ART for at least 180 days, with a CD4+ cell count > 100 cells/mm^3^, and acceptable screening labs, including specific LDL cholesterol and triglyceride levels based on ASCVD risk score. Exclusion Criteria: Clinical ASCVD, diabetes with LDL ≥ 70 mg/dL, ASCVD risk score > 15%, active cancer within 12 months (exceptions apply), known decompensated cirrhosis, recent myositis or myopathy, untreated symptomatic thyroid disease, severe allergy to statins, use of specific immunosuppressants, erythromycin, colchicine, or rifampin, use of statin drugs, gemfibrozil, or PCSK9 inhibitors within 90 days, recent serious illness or trauma, current pregnancy or breastfeeding, and conditions interfering with study completion.	NRP	CRP, Lp-PLA2, sCD163	NCT02344290	N/A

**Table 4:** Clinical evidence regarding statins’ effects on key inflammatory markers and cancer-related outcomes.

Statin Type and Dosage (If Specified)	Patient Population/Disease Context	Inflammatory Marker(s) Assessed	Observed Effect on Inflammatory Marker(s)	Cancer Outcome(s) Assessed	Observed Effect on Cancer Outcome(s)	Key Study/Evidence Type	Reference	Translational/Clinical Implication
Atorvastatin Simvastatin Rosuvastatin Fluvastatin Pravastatin Pitavastatin	Chronic Diseases	hsCRP, IL-6, TNF-α	Significant reduction (MD for CRP: −1.58 mg/L; IL-6: −0.24 ng/dL; TNF-α: −0.74 ng/dL)	N/A	N/A	Meta-analysis of RCTs	[224]	Statins exert broad anti-inflammatory effects across various chronic conditions.
Atorvastatin Simvastatin Pravastatin Rosuvastatin Fluvastatin Lovastatin Pitavastatin	Various (observational studies)	N/A	N/A	Cancer Incidence	Reduced incidence in 10/18 types, but evidence strength is low (*p* < 0.05 in some meta-analyses)	Umbrella Review (incl. observational)	[225]	Observational data suggests some incidence reduction, but this is not confirmed by higher-level evidence.
Atorvastatin Cerivastatin Fluvastatin Lovastatin Pravastatin Simvastatin	Various (RCTs)	N/A	N/A	Cancer Incidence, Cancer Deaths	No reduction (OR, 1.02 for incidence; OR, 1.01 for deaths)	Meta-analysis of RCTs	[226]	Randomized controlled trials do not support statins for primary cancer prevention.
Atorvastatin Cerivastatin Fluvastatin Lovastatin Pitavastatin Pravastatin Rosuvastatin Simvastatin	Advanced-stage Cancer Patients	N/A	N/A	Overall Survival, Cancer-Specific Survival, Progression-Free Survival	26% decreased risk for OS/CSS (HR 0.74); 24% for PFS (HR 0.76)	Meta-analysis of RCTs and Observational Studies	[227]	Statins may improve survival in advanced cancers, suggesting a potential adjunctive role.
Rosuvastatin 20 mg/day	Healthy with elevated hsCRP (≥2 mg/L), low LDL-C	hsCRP	Significant 37% reduction	Major Cardiovascular Events, All-Cause Mortality	44% reduction in CV events, 20% in all-cause mortality	JUPITER Trial (RCT)	[228]	Confirms hsCRP as a cardiovascular risk marker; Rosuvastatin demonstrates potent anti-inflammatory and cardiovascular protective effects.
Simvastatin 40 mg/day	Dyslipidemia or CHD	CRP	Most effective for lowering CRP (WMD = −4.07, 95% CI [−6.52, −1.77])	N/A	N/A	Network Meta-analysis (RCTs)	[229]	Simvastatin 40 mg is a strong option for C-reactive protein reduction in cardiovascular contexts.
Atorvastatin 80 mg/day	Dyslipidemia or CHD	CRP	Best long-term effect on CRP	N/A	N/A	Network Meta-analysis (RCTs)	[229]	Atorvastatin 80 mg is suitable for sustained C-reactive protein reduction.
Atorvastatin	Chronic Diseases (e.g., RA, Crohn’s)	IL-6, TNF-α	Most significant reduction (MD for IL-6: −5.39 ng/dL; TNF-α: −3.32 ng/dL)	N/A	N/A	Meta-analysis of RCTs	[224]	Atorvastatin is particularly effective for reducing Interleukin-6 and Tumour Necrosis Factor-alpha.
Fluvastatin 80 mg	Acute Coronary Syndrome (ACS)	CRP, IL-6	No significant difference on Day 2 and Day 30	Major Adverse Cardiovascular Events	Reduced 1-year occurrence (OR 0.40, 95% CI 0.17–0.95)	FACS Trial (RCT)	[230]	Anti-inflammatory effects may be less pronounced or slower to manifest in acute settings; cardiovascular benefits are still observed.
Fluvastatin	Chronic Diseases	CRP	Most pronounced impact (MD = −7.36 mg/L)	N/A	N/A	Meta-analysis of RCTs	[224]	Fluvastatin is potent for C-reactive protein reduction in chronic inflammatory conditions.
Simvastatin 40 mg + Chemotherapy (XP)	Advanced Gastric Cancer	N/A	N/A	Progression-Free Survival	No significant increase (median PFS 5.2 vs. 4.63 months; HR 0.930, *p* = 0.642)	Phase III RCT	[231]	Simvastatin 40 mg did not improve progression-free survival in this specific context.
Simvastatin 40 mg + Gefitinib	Advanced NSCLC (wild-type EGFR nonadenocarcinomas)	N/A	N/A	Response Rate, Progression-Free Survival	Higher RR (40% vs. 0%), longer PFS (3.6 M vs. 1.7 M) in subgroup	Phase II RCT	[232]	Potential benefit in specific non-small cell lung cancer subgroups, suggesting a personalized approach.
Simvastatin 40 mg + Afatinib	Advanced Nonadenocarcinomatous NSCLC	N/A	N/A	Response Rate, Progression-Free Survival, Overall Survival	No significant differences (RR 5.7% vs. 9.4%; PFS 1.0 vs. 3.6 months; OS 10.0 vs. 7.0 months)	Phase II RCT	[233]	Simvastatin dose may have been insufficient for anti-cancer effect in this setting.
Simvastatin 40 mg + Chemotherapy (Carboplatin/Vinorelbine)	Metastatic Breast Cancer	Baseline hsCRP	Elevated baseline hsCRP (>10 mg/L) associated with shorter OS	Objective Response Rate, Overall Survival	No beneficial increase in tumour sensitivity or OS (HR 1.16, *p* = 0.57)	RCT (double-blind, placebo-controlled)	[234]	Safe profile but no direct anti-tumour benefit; baseline hsCRP is a strong prognostic marker in this population.
Pitavastatin	Chronic Inflammation (skin, pancreas)	IL-33	Suppresses IL-33 expression by blocking TBK1-IRF3 pathway	Chronic Pancreatitis, Pancreatic Cancer	Linked to significantly reduced risk	Preclinical and Epidemiological Data	[235]	A novel mechanism for cancer prevention in inflammation-driven cancers, warrants further investigation.

**Table 5:** Statins exhibiting negative or non-significant results in different studies.

Statin	Type of Study	Disease	Statin’s Mode of Action	Reference Number
Simvastatin	Clinical Trial	COPD	Regular administration of 40 mg Simvastatin had no significant effects on the exacerbation rates in high risk-exacerbation COPD patients.	[430]
Simvastatin	Review	COPD	A large RCT reviewed showed no reduction in exacerbations for patients with COPD with no cardiovascular indication for statin treatment.	[431]
Atorvastatin	Clinical trial	Colorectal Cancer (CRC)	The therapeutic intervention did not yield significant reductions in (rectal aberrant crypt foci ACF) number when compared with control in a sample of patients with an increased risk for sporadic CRC	[432]
Atorvastatin	Clinical trial	Chronic Bronchitis (exposed to sulphur mustard gas)	Patients with chronic bronchitis exposed to sulphur mustard gas showed no significant reduction in levels of pro-inflammatory markers when treated with atorvastatin including interleukin-6 (IL-6) and tumour-necrosis factor α (TNF-α)	[433]
Atorvastatin	In vivo study (animal trial)	COPD	In mice treated with atorvastatin, no significant reduction in alveolar diameter or leukocyte count was observed, indication that the anti-inflammatory effects of atorvastatin in mice with elastase- induced emphysema may not be negligible.	[434]
Rosuvastatin	Clinical trial	COPD + Chronic Heart failure (HF)	The study demonstrated that 10 mg rosuvastatin was not effective in all cause, cardiovascular, non-cardiovascular death rates and hospitalization rates.	[84]
Rosuvastatin	Meta-analysis	Osteoarthritis (OA)	Sub-group analysis showed that rosuvastatin was associated with a higher risk of hip, knee or hand osteoarthritis.	[435]
Rosuvastatin	In vivo study	Systemic lupus erythematosus (SLE)	The study observed that while serum LDL-cholesterol was reduced in SLE mice, the serum inflammatory cytokines, proteinuria and clinical manifestation remained unchanged, indicating rosuvastatin is not efficient in treating SLE.	[436]
Rosuvastatin	Case–control study	Pancreatitis	The study found an increased risk of acute pancreatitis with rosuvastatin use.	[437]
Rosuvastatin	Clinical trial	Major Depressive disorder (MDD)	There was no improvement in Major depressive disorder (MDD) with rosuvastatin.	[438]

**Table 6:** Statins exhibiting negative or non-significant results when co-administered with different medications in different studies.

Statin	Type of Study	Disease	Statin’s Mode of Action	Reference Number
Simvastatin + Sarilumab	Clinical Trial	Rheumatoid arthritis (RA)	Sarilumab antagonizing IL-6 allows for overactivity of CYP450 (specifically CYP3A4), suppression of simvastatin levels and potential reduced efficiency of the drug.	[439]
Simvastatin + Narrowband UBD phototherapy	Clinical Trial	Psoriasis	No significant difference was observed in improvement of psoriasis in treatment group and placebo group.	[440]
Atorvastatin + Epigallocatechin-3-gallate (EGCG)	In vivo study (animal trial)	Colorectal Cancer (CRC)	No carcinogenic-inhibitory activity was observed in the mice treated with the combination of 0.1% EGCG and 60 ppm ATST. Some of the treated group even demonstrated enhanced tumour proliferation.	[441]
Atorvastatin + Inhaled corticosteroid + long-acting β-agonist	Clinical trial	Chronic obstructive pulmonary disease (COPD)	In the case of patients treated with 40 mg of atorvastatin no reduction in serum C-reactive protein and total antioxidant capacity was observed.	[442]
Simvastatin + Rosuvastatin + Atorvastatin	Meta-analysis	Cancer	Statin therapy for a duration of 5 years showed no significant evidence on incident or mortality on any type of cancer.	[443]
Simvastatin + Atorvastatin	Observational study (prospective cohort study)	Colorectal Cancer (CRC)	In this cohort of colorectal cancer patients, statin use was not associated with improved overall cancer-specific or recurrence-free survival.	[444]
Simvastatin + Rosuvastatin + Atorvastatin	RCT	Colorectal Cancer (CRC)	When reviewing meta-analysis on observational study, a mild risk reduction was observed. However, when reviewing the Randomized control trials focusing on statin use and colorectal cancer, no association was found. Overall, inconsistent results from different studies were observed, hence further research needs to be conducted.	[445]
Simvastatin + Rosuvastatin + Atorvastatin	Review	Chronic obstructive pulmonary disease (COPD)	Found that statins reduced level of inflammation in people with COPD, but this did not result in any clear improvements in exacerbations, mortality, functional capacity, quality of life, or lung function	[446]
Simvastatin + Rosuvastatin + Atorvastatin	Meta-analysis	Rheumatoid arthritis (RA)	Interleukin- 6 (IL6) and swelling of the joint (SJ) levels were not significantly reduced in rheumatoid arteritis patients with statin therapy.	[447]
